# In Vivo Imaging with Genetically Encoded Redox Biosensors

**DOI:** 10.3390/ijms21218164

**Published:** 2020-10-31

**Authors:** Alexander I. Kostyuk, Anastasiya S. Panova, Aleksandra D. Kokova, Daria A. Kotova, Dmitry I. Maltsev, Oleg V. Podgorny, Vsevolod V. Belousov, Dmitry S. Bilan

**Affiliations:** 1 Shemyakin-Ovchinnikov Institute of Bioorganic Chemistry, 117997 Moscow, Russia; alexander.kostyuk@inbox.ru (A.I.K.); panova_a@ymail.com (A.S.P.); ms.demidovich@inbox.ru (A.D.K.); kotovadaria95@gmail.com (D.A.K.); mal-dima@yandex.ru (D.I.M.); olegpodgorny@inbox.ru (O.V.P.); belousov@fccps.ru (V.V.B.); 2 Center for Precision Genome Editing and Genetic Technologies for Biomedicine, Pirogov Russian National Research Medical University, 117997 Moscow, Russia; 3 Federal Center for Cerebrovascular Pathology and Stroke, 117997 Moscow, Russia; 4 Institute for Cardiovascular Physiology, Georg August University Göttingen, D-37073 Göttingen, Germany

**Keywords:** fluorescent proteins, genetically encoded sensors, glutathione (GSH), hydrogen peroxide (H_2_O_2_), in vivo imaging, mycothiol (MSH), NADH, NADPH, reactive oxygen species (ROS), redox biology

## Abstract

Redox reactions are of high fundamental and practical interest since they are involved in both normal physiology and the pathogenesis of various diseases. However, this area of research has always been a relatively problematic field in the context of analytical approaches, mostly because of the unstable nature of the compounds that are measured. Genetically encoded sensors allow for the registration of highly reactive molecules in real-time mode and, therefore, they began a new era in redox biology. Their strongest points manifest most brightly in in vivo experiments and pave the way for the non-invasive investigation of biochemical pathways that proceed in organisms from different systematic groups. In the first part of the review, we briefly describe the redox sensors that were used in vivo as well as summarize the model systems to which they were applied. Next, we thoroughly discuss the biological results obtained in these studies in regard to animals, plants, as well as unicellular eukaryotes and prokaryotes. We hope that this work reflects the amazing power of this technology and can serve as a useful guide for biologists and chemists who work in the field of redox processes.

## 1. Introduction

Life as we know it is a constant flow of energy and matter that “permeates” every organism on Earth. Every second, multiple reaction ns proceed in all living systems, and crosstalk between these reactions generates the amazing complexity reflected in the incredible variety of morphological and anatomical structures, as well as in their ability to readjust in response to external factors. If one looks closely, the chemical events during which molecules exchange reductive equivalents in the form of electrons or hydride anions become noticeable. Some of these processes are “superstars” in the field of redox biology since they are well described in high school textbooks and in the lectures of science popularizers. For example the molecular machinery inside chloroplasts of green plants captures visible light to utilize its energy for the oxidation of water and the corresponding reduction of NADP^+^ [1] while O_2_, a byproduct of photosynthesis, serves as the final substrate for the mitochondrial electron transport chain (ETC) in the process of oxidative phosphorylation [2]. However, a keen eye will discover that the overwhelming presence of redox reactions extends far beyond these classical examples. Various specialized redox couples exist in cells, including NAD(H) [3], NADP(H) [4], glutathione [5], and thioredoxins [6] among others, each with its own specialization and chemical properties. In recent years, increased attention has been paid to reactive oxygen species (ROS), highly reactive compounds with dual roles [7]. In some situations, organisms produce them on purpose to confront pathogens [8] or to induce signal transduction [9], but at the same time a misbalance between ROS generation and the capacity of antioxidant systems can result in severe diseases [10]. It seems that the electron flows “wrap” most, if not all chemical pathways in cells, therefore influencing life at each “step” from the birth to death. 

What instruments do we have in our arsenal to decipher redox processes? Implementation of traditional analytical methods in this field faces significant practical drawbacks. Compounds like H_2_O_2_, NO, or ONOO^−^ are too reactive to be purified and measured directly, and other redox active metabolites can be oxidized by oxygen, which leads to inaccurate results. Moreover, the implementation of different sample preparation protocols and reagents with unequal selectivity introduces additional inconsistency. Modern omic approaches allow gigabytes of data to be obtained, however they capture the picture at a single moment of time and scaling is extremely labor-intensive. At the same time, imaging provides a unique opportunity to follow fast chemical events in cells, therefore revealing the dynamic nature of life. Various low molecular weight dyes that change optical properties have been developed, but they often lack the desired selectivity and reversibility. In addition, the process of dye loading is invasive and may disturb metabolism. There are different strategies for dye targeting to subcellular compartments, but they come with significant difficulties. 

Genetically encoded fluorescent indicators (GEFIs) began a new era in redox biology research. As protein molecules with auto-catalytically formed chromophores, they provide multiple advantages:

1. This technology is not invasive and does not introduce artifacts caused by sample preparation. A classic example in this area is measurements of glutathione redox potential (*E_GSH_*) using redox active fluorescent proteins (FPs). In contrast to traditional approaches, these sensors revealed that the cellular glutathione pool is highly reducing, and reaches a reduced/oxidized glutathione (GSH/GSSG) ratio of between 50,000:1 and 500,000:1 [11].
2. GEFIs can be targeted to different subcellular compartments via the host sorting machinery. There are studies where sensors were expressed in the matrix [12,13,14,15,16,17,18,19,20,21,22,23,24,25,26,27,28,29,30] and in the intermembrane space [31,32,33] of mitochondria (IMS), in the endoplasmic reticulum (ER) [34,35,36,37,38,39,40,41,42,43], peroxisomes [12,19,24,44], apicoplasts [16,45] and the nucleus [46]. Since many redox processes proceed in specific parts of the cell, whole cell approaches often lack sufficient sensitivity to record pronounced but localized events. Moreover, even the traditional analytical methods that take subcellular heterogeneity into account can suffer from unequal behavior of different organelles during sample preparation.
3. In a cell population, the GEFIs signals reflect the state of each individual member of the group rather than the averaged data. This property paves the way for implementation of sorting algorithms [47,48,49] and allows visualization of heterogeneity in the neighboring regions [49,50,51,52].
4. The genes coding for GEFIs can be placed under the control of specific promoters. Therefore, it is possible to express these instruments in only the desired subsets of cells. Some examples include keratinocytes [53,54], pancreatic beta cells [55], muscle cells [22,23,56], neutrophils [57], heart tissue [58] and neurons [59,60,61,62].
5. Modern genetic engineering approaches allow transgenic organisms to be created, which provides both signal stability and reproducibility of results. This approach has already been implemented in many traditional model organisms: *Mus musculus* [20,21,53,54,59,60,62], *Drosophila melanogaster* [61], *Danio rerio* [57,58,63,64,65,66,67,68,69], *Caenorhabditis elegans* [22,23,50,56,70], *Xenopus laevis* [71,72], *Pasmodium falciparum* [17,73], and *Corynebacterium glutamicum* [74] among others.
6. Experiments with GEFIs can be extended for recording several parameters simultaneously during multiparameter imaging [69,75,76]. This is of great importance since correct interpretation of data on fast biochemical events collected in separate series can be quite difficult [77].
7. Finally, GEFIs are compatible with low molecular weight chemical dyes and many other experimental approaches, therefore, facilitating study design.

All the references provided above represent a small portion of the existing research. Generally, the field of implementation of redox sensors is so large that it cannot be described in a single review or maybe even a single book. Therefore, in this paper, we focus only on studies in which redox GEFIs were used with in vivo imaging models, this is where their strongest points manifest most brightly. We hope that this work reflects the power and the beauty of the technology and can become a useful guide for redox biologists.

## 2. A Short Overview of Redox GEFIs Applied In Vivo

Many reviews describing the diversity of existing biosensors and their applications have been written [11,78,79,80,81,82,83,84] and in this section we summarize information about GEFIs that were used in vivo.

Today, the most widely used in vivo redox probes belong to the redox-sensitive green FP (roGFP) and HyPer families. The roGFP family includes redox-sensitive proteins, more or less selective to *E_GSH_* [85,86,87,88] and probes that are relatively specific to other thiols [52,89,90] or H_2_O_2_ [91,92], which were developed by fusing roGFPs with different adapter proteins. The first roGFP variants were developed in 2004 by introducing redox-active cysteine residues to the GFP β-barrel surface in spatial proximity to the chromophore [85] (a similar design was applied for the first time to yellow FP (YFP) by Winther et al. in 2001, giving the redox-sensitive YFP (rxYFP) probe [93]). Thus, a number of roGFPs were developed, and roGFP1 and roGFP2 gained the most widespread popularity in further research. roGFP1 and roGFP2 demonstrate a ratiometric signal of relatively high amplitude, moreover, the roGFP1 signal is not pH dependent [85].

Two special improvements in the roGFP1 have subsequently been reported, namely, the roGFP1-Rx [86] and roGFP1-iX [87] groups, with the former group consisting of variants with improved response rate, and the latter group demonstrating less negative midpoint redox potentials, allowing for the use of roGFP1-iN in relatively oxidizing subcellular compartments, such as the ER and Golgi apparatus. In 2008, an important optimization of roGFP2 was published: a fusion protein consisting of human glutaredoxin 1 (Grx1) and roGFP2 showed a significantly higher response rate and selectivity to glutathione [88]. The idea behind the probe was as follows: human Grx1 specifically catalyzes the transfer of oxidative equivalents between the glutathione redox pair and roGFP2, thus, the spatial proximity of the enzyme and the sensor molecule leads to a more rapid and specific equilibration. The obtained probe, Grx1-roGFP2, allowed ratiometric pH-independent (pH 5.5–8.5) measurements and became one of the commonly applied in vivo redox biosensors. In a similar way to Grx1-roGFP2, probes to assess the redox potential of *Mycobacterium tuberculosis* mycothiol (Mrx1-roGFP2 [52]), *Staphylococcus aureus* bacillithiol (Brx-roGFP2 [89]), and trypanosome trypanothione (Tpx-roGFP2 [90]), were developed.

The roGFP-based probes specific to H_2_O_2_, roGFP2-Orp1 [91] and roGFP2-Tsa2ΔC_R_ (and the ΔC_R_ΔC_P_ variant) [92], have been described and used in vivo. RoGFP2-Orp1 [91] was designed as a fusion protein consisting of roGFP2 and Orp1 peroxiredoxin. Orp1 was found to efficiently mediate electron flow between H_2_O_2_ and roGFP2, creating a special redox relay when placed in close proximity to the target protein. The indicator allows ratiometric measurements and is widely used in different model organisms for H_2_O_2_ registration. Recently, an ultrasensitive probe with a similar design idea has been reported [92]. The yeast peroxiredoxin Tsa2 was fused to roGFP2 to mediate the oxidation of the latter. The fundamental difference is that Tsa2 is a 2-Cys peroxiredoxin, which are the most efficient thiol peroxidases and react with H_2_O_2_ 100 times faster than Orp1. The indicators roGFP2-Tsa2ΔC_R_ and ΔC_R_ΔC_P_ variant, enable the detection of endogenous basal H_2_O_2_ levels, which may give new insights into H_2_O_2_ biology. However, since the probes were developed based on the yeast platform, some optimization may be needed for their use in other model organisms.

In 2017, some limitations for in vivo application of the Grx1-roGFP2 and roGFP2-Orp1 probes were observed by Müller at al. [94]. It was found that micromolar amounts of hypochlorous acid and polysulfides contribute to roGFP2-based probe responses in vitro. Peroxynitrite may also influence the probes redox state, especially for roGFP2-Orp1. The required concentrations of HOCl, for instance, can be produced during the phagocyte respiratory burst. These facts imply that these indicators should be used carefully in physiological conditions, pathological conditions, or in specific cell types, where high amounts of the aforementioned oxidants are generated [94].

In addition to roGFP family probes, there are indicators constructed in a similar way. Among these are oxidation-sensitive Oba-Q reporters [95], the fluorescence of which is drastically quenched upon oxidation, as reported by Sugiura et al. The Oba-Q indicators are based on blue-shifted FPs, so multiparameter imaging is possible using Oba-Q in combination with biosensors of different colors. Two red probes, redox sensitive red FP (rxRFP) [96] and Grx1-roCherry [97] have been applied in vivo. RxRFP was based on circularly permuted (cp) red FP cp-mApple with cysteine residues introduced on its mutated N- and C-termini [96]. Grx1-roCherry was constructed as a fusion protein consisting of human Grx1 and the redox-sensitive red FP roCherry [97]. Both probes are redox-sensitive, and Grx1-roCherry demonstrates a sufficient response rate and relative specificity to glutathione redox potential due to the Grx1 domain. Red probes are preferable in some conditions because, firstly, their excitation and emission light is less toxic for living objects, gives less autofluorescence, and penetrates tissues better, and secondly, red indicators allow for multiparameter imaging when used in combination with blue and green indicators.

A novel redox-sensitive probe, which was based on the Japanese eel FP UnaG [98] has been reported by Hu et al. [99]. Like other redox indicators, redox-sensitive UnaG (roUnaG) contains a pair of cysteines on the β-barrel surface, which form a disulfide bond and change the chromophore spectral properties. Interestingly, UnaG emits light only upon binding of bilirubin, which acts as an external chromophore. This makes it possible to use roUnaG to monitor the redox state in hypoxic conditions, which may be a good alternative to GFP-based proteins because the GFP chromophore requires the molecular oxygen for maturation. However, there may be some limitations due to the necessity of bilirubin introduction.

The HyPer family is a group of H_2_O_2_ sensing probes [66,100,101,102,103]. The first HyPer was designed as a chimera consisting of a regulatory domain of the *Escherichia coli* H_2_O_2_ sensing transcriptional factor OxyR (OxyR-RD) and cpYFP, integrated into a flexible region of the former [100]. In the primary structure of OxyR-RD there are two key cysteine residues, which form a disulfide bond under H_2_O_2_-mediated oxidation, with consequent dramatic conformational changes in the protein structure. The conformational rearrangement triggers a change in the cpYFP chromophore microenvironment and, accordingly, fluorescence intensity. Two upgrades of HyPer, namely HyPer-2 [101] and HyPer-3 [66], with improved maximum response amplitude and kinetic properties were subsequently reported. In 2020, an ultrasensitive pH-stable probe HyPer7, characterized by significantly enhanced brightness, kinetics, and sensitivity was described by Pak et al. [102]. The fundamental difference of HyPer7 from previous HyPers is that its sensory domain is represented by *Neisseria meningitidis* OxyR-RD, which presumably has a higher sensitivity to H_2_O_2_ than its *E. coli* homolog, although this fact has not been known a priori. The red probe HyPerRed [103], which is based on cp-mApple, also has also been applied in vivo. Except for HyPer7, all HyPers respond to pH alterations, so experiments with the H_2_O_2_-insensitive HyPers-C199S, or SypHers, are needed to control for possible artifacts. In HyPers-C199S one of the two key cysteines is replaced with serine, which makes the probe unresponsive to H_2_O_2_, retaining the same pH dependency of fluorescence ratio. HyPer-3, HyPer7, and HyPerRed could be used in combinations to study several redox processes simultaneously. While the application of HyPerRed with either green variant allows real-time imaging in different organelles, HyPer7 and HyPer-3 allow H_2_O_2_ dynamics to be monitored in conditions where different sensitivity of the indicator is needed.

Using HyPer, HyPer-2, HyPer-3, and HyPer7 as an example, one can see how the gradual improvement of the biosensor makes it more and more optimal for its use in vivo. HyPer-2 differs from HyPer in its doubled response amplitude, which improves the target signal-to-noise ratio in living systems, especially at low H_2_O_2_ concentrations. HyPer-3 retained the high amplitude and showed faster oxidation/reduction dynamics, which made it possible to track changes in H_2_O_2_ levels more quickly. HyPer7 is rather brighter compared to its predecessors, which is an important improvement because it significantly facilitates its visualization in tissues. Moreover, the pH stability of the probe allows its use without pH-control, and this is rather important in animal models, because the more complex the organism, the less likely it is to make good control for the experiment since different individuals differ more from each other.

TriPer, a probe that reports H_2_O_2_ dynamics in the thiol-oxidizing environment of the ER, was developed by Melo et al. [104]. The original HyPers are fully oxidized in such conditions, and the solution found by the authors was as follows: the third cysteine was introduced into the OxyR-RD moiety, which initially contains two redox active cysteines that form a disulfide bond under oxidation. The introduction of the third cysteine may permit rearrangement of the disulfide bonding pattern and preserve a fraction of the indicator in its reduced form even in oxidizing conditions. Another H_2_O_2_-sensing probe, NeonOxIrr, has been published recently [105]. NeonOxIrr is characterized by a slow reduction rate and allows H_2_O_2_ registration ex vivo in fixed samples.

Besides H_2_O_2_ and glutathione redox state sensing probes, sensors for NADH/NAD^+^ registration are commonly used, as this ratio serves as a metabolic and redox marker. Most representatives of this group are based on the bacterial repressor protein Rex. In the ligand binding site of Rex, competition between NAD^+^ and NADH occurs, and when the NADH/NAD^+^ ratio changes, the protein undergoes dramatic conformational rearrangement [106]. Peredox was constructed by inserting the circularly permuted FP cpT-Sapphire between two *Thermus aquaticus* Rex (T-Rex) subunits with subsequent optimization and fusing mCherry to the construct for fluorescence signal normalization [107]. The probe had remarkably high affinity to NADH. This may be beneficial as Peredox senses minimal changes in NADH levels, but in some conditions with initially high NADH levels it could limit the application of the probe due to saturation. This could happen when using the indicator in mitochondrial matrix [107] or in bacteria [108] for instance. 

In a similar manner to Peredox, Frex and FrexH were developed by integration of cpYFP between one full-length and one truncated *Bacillus subtilis* Rex subunits, although, these probes are specific to NADH only [109]. These indicators have an intensiometric response and are different in affinity to NADH and as well as in the direction and amplitude of response, so one could use either of them in appropriate experimental conditions. RexYFP is an intensiometric NADH/NAD^+^ reporter, which was based on a single T-Rex subunit with cpYFP integrated between DNA- and ligand-binding T-Rex domains [110]. Thus, the RexYFP protein molecule is smaller than that of Peredox and Frex, which may facilitate its use when targeting to different subcellular compartments. The affinity of RexYFP to NADH (K’ = 180 nM) allows its implementation both in cytoplasm and mitochondria. The indicator also demonstrates low affinity to NADPH (K’ = 6.2 µM), which should not interfere in most experiments. However, since the NADP(H) pool is more reduced than NAD(H) pool in the cytoplasm, sensitivity to NADPH could alter the signal under certain conditions. Another probe, SoNar, was also constructed by inserting cpYFP into a T-Rex monomer but in this case the fluorescent core was placed in the surface loop of the nucleotide-binding domain, while the DNA-binding domain was truncated [111]. The distinct feature of SoNar is the remarkably high amplitude of its ratiometric response (~15-fold) that makes its use in vivo rather preferable. Later, SoNar specificity was altered by rational mutagenesis and iNaps, which are NADPH sensors with different affinities, were reported [75]. Like SoNar, iNaps are characterized by ratiometricity of the fluorescent signal and high response amplitudes. Recently, an NAD^+^/AXP reporter was developed by Zou et al. [112] where AXP refers to the total pool of ATP and ADP. FiNad was obtained by optimizing one of the versions that preceded SoNar via random mutagenesis of short amino acid linkers between T-Rex and cpYFP. Then, mCherry was fused to the sensor to achieve a ratiometric fluorescence response. The authors suggest that FiNad actually reports NAD^+^ level shifts since the total physiological AXP pool is generally maintained in homeostasis.

Along with redox-sensitive proteins and families of indicators specific to H_2_O_2_ and NAD(H), there are several probes that are specific to other redox parameters. Indicators for R- and S-diastereomers of methionine sulfoxide were reported by Tarrago et al. [113]. Methionine sulfoxide is formed during oxidation of methionine by biological oxidants both under physiological and pathological conditions and may serve as redox biomarker. Each probe, MetSOx or MetROx, was constructed as a chimera consisting of *Saccharomyces cerevisiae* methionine sulfoxide reductase (MSRA or MSRB, specific to the diastereomer), its specific thioredoxin (Trx1 or Trx3) and cpYFP located between them. The following mechanism of action is expected for MetSOx and MetROx: under the influence of methionine sulfoxide, two redox active cysteines of MSR form a disulfide bond, which is subsequently reduced by a corresponding thioredoxin (Trx). During the process of reduction, a new disulfide is formed between Trx and methionine sulfoxide reductase, and this is accompanied by conformational changes that trigger a shift in the fluorescence signal. A probe that enables a visualization of organic hydroperoxides in living systems was developed by Simen Zhao et al. [114]. In OHSer, cpVenus was placed in the most conformationally mobile region of the bacterial transcriptional factor OhrR, which is specific to organic hydroperoxides. Thus, the conformational change that occurs in the OhrR moiety after its interaction with organic hydroperoxides leads to a change in the spatial structure of cpVenus and the fluorescence signal.

Besides single FP-based biosensors, there are a number of Förster resonance energy transfer (FRET)-based redox-sensitive probes used in vivo. Redoxfluor, reported by Yano et al., has been used to monitor the redox states of peroxisomes [44]. The biosensor molecule consists of Cerulean and Citrine FPs and a tandem repeat of part of the yeast transcriptional factor Yap1 between them. The Yap1 part is capable of undergoing conformational rearrangement during oxidation-reduction events represented by internal disulfide bond formation, and this results in a FRET response of the probe. A specific NADP^+^-sensing FRET-based probe, NADPsor [115], was designed by inserting the NADP^+^-reporter element, ketopantoate reductase, between the FRET pair cyan FP and YFP. When binding to NADP^+^, ketopantoate reductase changes its conformation from relatively closed to open, so FPs move away from each other and the FRET between them weakens. A probe for measurements of the thioredoxin redox state in chloroplasts, CROST [116], has been recently published by Sugiura et al. CROST contains the mTurquoise/cp-mVenus pair and partial sequence from CP12, a redox-sensitive regulatory and thioredoxin-targeted protein in chloroplasts, between FPs. As in other fluorescent biosensors, the reduction of internal cysteines in the CP12-derived domain by thioredoxin leads to conformational alterations in the molecule, and corresponding changes in the FRET ratio.

The list of biosensors described in this section, as well as some of their key parameters and examples of their in vivo applications, can be found in Table 1 and Table 2.

## 3. Redox Biosensors in Animals

### 3.1. Embryogenesis, Development and Aging

It is well known that hydrogen peroxide not only damages cells during many pathological processes but also contributes to the redox signaling under normal conditions. Interestingly, H_2_O_2_ affects embryonic development throughout the process, starting from the zygote stage. It has recently been revealed by De Henau et al. that this molecule is important in zygote symmetry breaking [129]. The mitochondria were found to relocate during polarization and create dynamic and functional H_2_O_2_ gradients. Local H_2_O_2_ elevation produces a physiological signal that leads to symmetry breaking and establishment of polarity. The authors suggest that the role of mitochondrial H_2_O_2_ in symmetry breaking might be highly conservative throughout animals, relying on some evidence of mitochondria enrichment [265] and embryo sensitivity to H_2_O_2_ [266] in other species.

Han et al. investigated the involvement of mitochondrial H_2_O_2_ into the regulation of the early embryonic cell cyle in *X. laevis* [151]. The fertilization-induced Ca^2+^-wave was shown to be necessary and sufficient to produce mitochondrial ROS, the concentrations of which oscillated with the cell cycle. Inhibition of ROS production led to cell cycle-arrest, revealing a regulatory role of ROS. H_2_O_2_ was also shown to participate in some signaling pathways that govern morphogenesis. Until recently, relatively little was known about the spatio-temporal distribution of hydrogen peroxide and its function during embryonic development. Gauron et al. demonstrated that H_2_O_2_ levels increase markedly during morphogenesis, especially in somito- and organogenesis [140]. It was revealed that H_2_O_2_ affects axon growth and thus plays a role in neuronal network formation, at least at the tectum, which is associated with modulation of the Hedgehog signaling pathway.

Hydrogen peroxide does not only play a role in embryogenesis but also participates in the subsequent life events. By combining the quantitative proteomic technique and fluorescence imaging with HyPer, Knoefler et al. demonstrated that during development *C. elegans* undergoes high oxidant levels, and this might influence the lifespan [123]. The authors revealed that when worms reach the reproductive period, the H_2_O_2_ concentration rapidly drops and remains low until aging begins and H_2_O_2_ accumulation reoccurs. Moreover, the long-living daf-2 mutants showed a more significant decrease in steady-state peroxide levels upon transition to the mature state compared to the daf-16 short-lived ones, which failed to fully recover from late developmental oxidative stress. These findings suggest that the ability to persist oxidative stress might correlate with the later life, including lifespan prolongation. Later, Bazopoulou et al. confirmed the impact of early developmental ROS on the increase of stress resistance and corresponding lifespan prolongation in *C. elegans*. The interrelation of the mentioned events and a decrease of histone H3K4me3 levels was established [122]. The global H3K4me3 levels were found to be redox-sensitive and decrease with oxidative stress, which led improvment in stress-resistance. However, Back et al., the first group which used HyPer in *C. elegans*, did not find any changes in H_2_O_2_ levels during postembryonic development [120]. Instead, the authors observed a logarithmical increase in the GSH/GSSG ratio throughout larval growth that might be explained by a decrease in the number of differentiating cells, which are characterized by low GSH/GSSG reduction potential, and gametogenesis, which is also accompanied by glutathione pool reduction. The lack of coincidence in H_2_O_2_ and glutathione redox state dynamics may reflect that the peroxide is not directly or necessarily detoxified by GSH, which is confirmed by the study described below.

**Figure 1:**
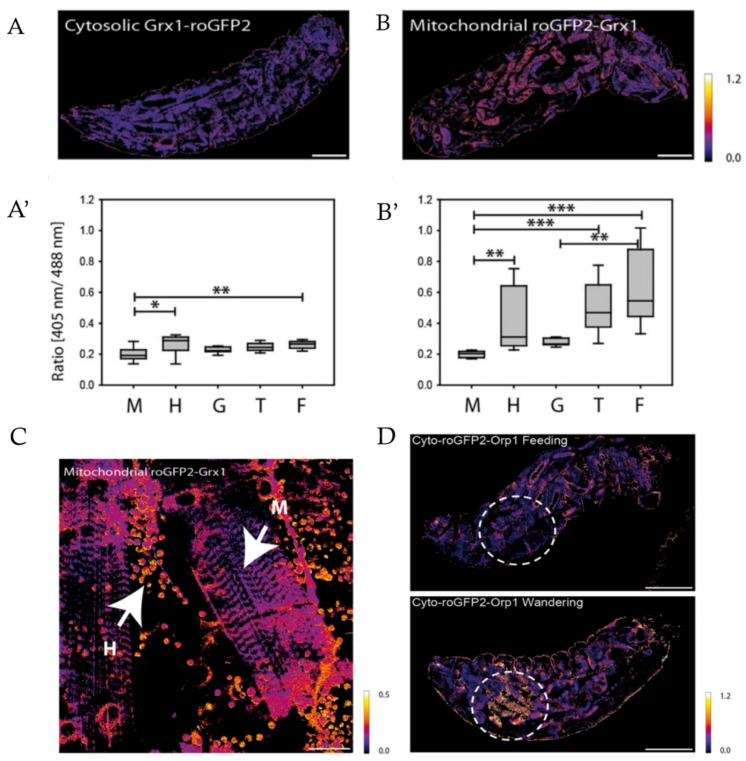
(**A**,**B**) Ratio images of whole intact larvae expressing cyto-Grx1-roGFP2 (**A**), mito-roGFP2-Grx1 (**B**). Probe responses in selected tissues were quantified in ten individual third-instar larvae for each biosensor (**A’**,**B’**). Boxes, lower/upper quartile; whiskers, 5th/95th percentile. * *p* < 0.05, ** *p* < 0.01, *** *p* < 0.001. M, muscle; H, hemocytes; G, gut; T, (Malpighian) tubules; F, fat. Scale bars, 600 µm. (**C**) A representative detailed image to highlight tissue-specific differences evident in mito-roGFP2-Grx1-expressing larvae. Arrows indicate hemocytes (H) and muscle tissue (M). Scale bar, 60 µm. (**D**) Feeding (top) and wandering (bottom) third-instar larvae expressing the cytosolic roGFP2-Orp1 probe. Scale bars, 600 µm. Reprinted by permission from Elsevier: Cell Metabolism [18], copyright 2011.

Albrecht et al. investigated both the H_2_O_2_ levels and GSH/GSSG ratio changes in living *D. melanogaster* larvae [18]. The glutathione redox state of third-instar larvae has been shown to be highly reduced in the cytoplasm with little variations within and between tissues. In contrast, in mitochondria, the GSH/GSSG ratio was clearly heterogeneous within and between different tissues. The authors also examined the redox-state fluctuations during larval development, in particular comparing feeding larvae and larvae that had just entered the wandering stage. The latter exhibited high cytosolic H_2_O_2_ production within a delimited patch of anterior adipose tissue, while minor changes were observed in mitochondrial H_2_O_2_ and no changes at all in GSH/GSSG ratio. These results allowed the authors to make the conclusion that differences in the redox state are distinct in terms of tissue type, subcellular compartment, and redox chemistry. The alterations in H_2_O_2_ concentrations and glutathione redox state were not necessarily consistent, which could be true for other molecules involved in the regulation of cell redox homeostasis, suggesting that to obtain a relatively full picture, ideally one needs to evaluate the dynamics of all redox species separately and specifically (Figure 1). 

The dependence of lifespan on the early ontogenetic events was also demonstrated by monitoring the redox-state changes in *C. elegans* under increasing growth temperature [121]. Worms cultivated at 25 °C during development and then transferred to 15 °C were found to show a higher lifespan compared to those constantly cultivated at 15 °C. It was revealed that, at 25 °C, the larvae and young adults had a more reduced glutathione pool and higher expression levels of Prxd-2, which is responsible for H_2_O_2_ detoxification. Moreover, the lifespan of prdx-2 mutants did not increase with changing temperatures. Henderson et al. suggested that the H_2_O_2_ level rose with increasing temperature, causing the activation of the oxidative stress response, Prdx-2 accumulation during development, and an adjustment to more reducing redox environments during development.

The interrelation between redox and Ca^2+^-signaling during *D. rerio* development was investigated by Yadav et al. [134]. The Chac1 enzyme, which is known to only degrade reduced glutathione, was found to act in the upstream activation of Ca^2+^-signaling. Chac1 expression in developing *D. rerio* (zebrafish) was largely restricted to the brain, heart and myotome, and the developmental defects in knockdown organisms were seen primarily in these organs. It is known that Ca^2+^ plays important roles in the development of the brain, heart and myotome, and the absence of an oxidizing environment as well as an almost complete lack of calcium transients were observed in these conditions. Thus, it was hypothesized that a Chac1-mediated GSH/GSSG decrease is required for Ca^2+^-signaling, which is essential for zebrafish development. The fact that alterations in *E_GSH_* were observed strictly in specific organs emphasizes the value of GEFIs utilization in live organisms.

Like developing worms, adult worms demonstrate some age-associated organismal redox-state variations. For instance, oxidative stress resistance as well as the morphology and density of neuronal mitochondria were showed to change in a three-phasic way according to the data by Morsci et al. [118]. These parameters increased in early adulthood and then high levels were maintained until late adulthood, when the parameters decreased. Interestingly, the dynamics of resistance to oxidative stress differed in long-living daf-2 mutants and WT worms. The daf-2 mutants showed constitutively higher resistance to acute oxidative shock and a slower age-related decline in resistance to oxidative stress. The authors attribute such dynamics to the larger size of middle-aged worm mitochondria, which implies more antioxidants and more anti-ROS capacity. In another study by Jiang et al., progressive mitochondrial fragmentation in neurons and body wall muscles of *C. elegans* has been reported as a hallmark of aging [119]. By using roGFP, mitochondria in old neurons were found to be more oxidized compared to young neurons, confirming that these organelles deteriorate during aging.

An interesting study, in which the interrelation between cytosolic and ER redox state throughout the lifespan of *C. elegans* was explored, was performed by Kirstein et al. [34]. During development, the redox state of both mentioned compartments was subject to profound fluctuations. The ER was more oxidized on the first day of larvae life, after which it shifted to slightly more reducing conditions. The second peak of oxidation was reached in young adults, followed by subsequent reduction. The cytoplasm redox state was found to change in the opposing manner: while the ER became more reduced, the cytosol became more oxidized. From these data, and on the basis of other experiments with proteotoxic challenges, it was concluded that redox homeostasis in the ER and cytosol is coupled.

**Figure 2:**
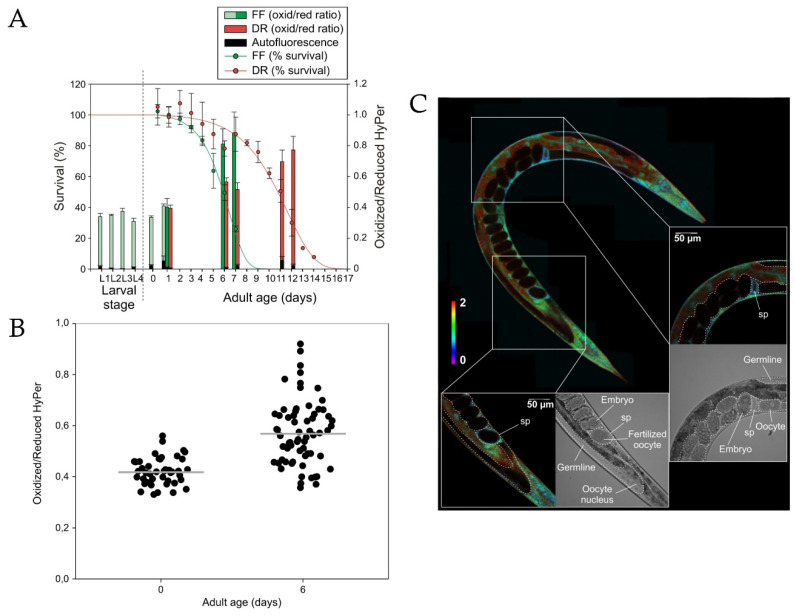
(**A**) Survival (scatter plot with Gompertz fit) and H_2_O_2_ levels (stacked bars) during development and aging. Diet-restricted (DR) worms live 76% longer than fully fed (FF) HyPer transgenic worms (*p* < 0.0001; log rank test). H_2_O_2_ levels did not change significantly during postembryonic development (L1–day 1; light green bars; slope = 0.0004 ± 0.006; *p* = 0.94; linear mixed model (LMM)). H_2_O_2_ levels increase significantly with age in FF (dark green bars; slope = 0.083 ± 0.028; *p* = 0.0002; LMM) and DR HyPer worms (red bars; slope = 0.030 ± 0.009; *p* = 0.005). Although FF and DR worms have similar H_2_O_2_ levels at the first day of adulthood (*p* = 0.78), DR significantly attenuated the age-related increase in H_2_O_2_ (*p* = 0.016). The portion of autofluorescence in the total ratio value is indicated with black bars. Error bars represent standard error of the mean (SEM). (**B**) Confocal analysis of young and old HyPer worms. Dot plot of mean individual H_2_O_2_ levels of young (day 0) and old (day 6) adults scanned with the same laser settings and quantified over the whole worm body; the population mean is indicated by a horizontal line. H_2_O_2_ levels, averaged over the population, significantly increase with age. Day 0, 0.42 ± 0.008; day 6, 0.57 ± 0.016; *p* < 0.01 (paired t test). (**C**) Spatial patterns of GSSG/2GSH ratios in young (first day of adulthood) Grx1-roGFP2 transgenic adults. Intensity-normalized ratio false-colored image of one Z-level of 3D-stitched worms. The anterior and posterior spermathecae (sp) show low GSSG/2GSH ratios. Calibration bar indicates the ratio of 525-nm emission after excitation at 405 nm vs. 488 nm. Reprinted by permission from Elsevier: Free Radical Biology & Medicine [120], copyright 2011.

In models of aging, cellular redox state has also been examined using GEFIs. Back et al. demonstrated that in *C. elegans*, H_2_O_2_ levels increased with age, most likely due to diminished detoxification rather than increased peroxide production [120]. Dietary restriction without malnutrition, which is considered a way to prolong the lifespan, was found to delay the increase in H_2_O_2_ concentration with age. Keeping in mind the study by Schulz et al. that investigated the correlation between dietary restriction, ROS formation and detoxification, and increased oxidative stress resistance [18], it could be suggested that increased ROS concentrations were better detoxified by a more pronounced antioxidant response. In young adult worms, local regions with high H_2_O_2_ concentrations and high GSH/GSSG ratios were found, which are probably related to their function in osmoregulation, cuticle formation, and fertilization. Despite the overall redox state in old worm individuals being more oxidized than in young individuals, the authors could not find distinct H_2_O_2_ patterns in old worms, which could indicate that during aging peroxide concentrations increase globally rather than locally. Due to the lack of fixed spatiotemporal patterns of H_2_O_2_, it was suggested that individual worms could show different aging rates in different tissues (Figure 2).

In contrast to the study by Back et al., Albrecht et al., who investigated H_2_O_2_ and GSH/GSSG dynamics in *D. melanogaster*, revealed that pro-oxidative changes during aging are restricted to particular tissues [18]. Midgut enterocytes showed pronounced elevation of cytosolic H_2_O_2_ concentration during aging despite the change in cytosolic GSH/GSSG being much less distinct. It was also found that increased lifespan in females was accompanied by an increase rather than a decrease in H_2_O_2_ concentrations, which is not consistent with the oxidative damage theory of aging. Because H_2_O_2_ formation could be causally connected to some biological function like innate immune response, driven by increased bacterial load in the gut with age, the authors suggest the increased H_2_O_2_ levels could be an epiphenomenon rather than a cause of the aging process.

Cabreiro et al. carried out a study in which lifespan extension by superoxide dismutase overexpression in *C. elegans* was revealed, but, unexpectedly, this was not caused by O_2_^•−^ elimination and enhanced antioxidant defense [124]. Sod-1 overexpression increased protein oxidation and steady-state levels of H_2_O_2_, but by using HyPer, it was shown that an increase in H_2_O_2_ concentration does not influence lifespan. Instead, evidence was found that unfolded protein response (UPR) activation under sod-1 overexpression has a positive effect on longevity. This work, in which the complex effects and unexpected consequences of antioxidant gene overexpression are discussed, underlines importance of careful interpretation of the results of such studies.

Ewald et al. recently found that, along with mitochondrial ROS activation of the stress response and promotion of longevity, the NADPH-oxidase (NOX) derived ROS also leads to the cell protection [126]. The authors identified a mechanism in *C. elegans* for the regulation of the NOX-derived ROS production and initiation of a transcriptional response which led to an adaptation similar to that caused by mitochondrial ROS.

Redox GEFIs were also used in other studies in which some interesting phenomena connected to development and aging were explored. For instance, roGFP2-Orp1 was used in a study by De Henau et al., in which the globin GLB-12 was observed to produce O_2_^•−^ from molecular oxygen in the somatic gonad of *C. elegans* [128]. The generated O_2_^•−^ signal was then modulated by intra- and extracellular superoxide dismutase, as a result of which a transmembrane H_2_O_2_ gradient was created that served as a redox signal that regulated reproduction, including germ cell apoptosis. The authors hypothesize that the restricted tissue and subcellular location of GLB-12 served as a spatial determinant for downstream signals. This is in line with the idea that ROS signal specificity can be reached by tightly regulated spatiotemporal expression of ROS generators in close proximity to their targets. HyPer was used in an investigation of a mechanism of proteostasis control during aging through mRNA processing body-mediated regulation of protein synthesis in the *C. elegans* soma [125]. In this study, the results obtained using a biosensor, combined with other results, indicated that mRNA decapping modulated aging and stress responses through a multilayered mechanism relying on the activity of certain stress response regulators.

### 3.2. Inflammation

ROS play a crucial role in inflammatory responses as they have antimicrobial properties and are produced by phagocytic cells to directly kill pathogens [267], recent evidence also confirms that ROS act as second messenger molecules involved in various inflammatory signaling pathways [268]. However, overproduction of ROS may lead to oxidative stress and tissue damage [269]. The first inflammation model using redox GEFIs was a tail fin amputation model in *D. rerio* larvae established by Niethammer et al. [141]. Later, this model gained popularity as a method for testing newly developed redox biosensors in vivo [66,75,102]. 

Niethammer et al. demonstrated that wounding zebrafish larvae induced production of hydrogen peroxide, which was detected by the HyPer probe. H_2_O_2_ concentration increased immediately after injury and peaked at approximately 20 min post-amputation. It was previously thought that inflammatory ROS originated from leukocyte oxidative burst, however, in this model it was revealed that leukocytes were recruited to the wound after an increase in H_2_O_2_ concentration [141]. As an alternative, enzymes of the NOX family, known to generate superoxide or H_2_O_2_ [270], were considered as probable candidates for ROS production [141]. As a result, Duox enzyme, a member of this family that is expressed in epithelial cells, was identified as the main source of ROS. Pharmacological inhibition or knockdown of Duox resulted in impaired leukocyte recruitment to the wound indicating that H_2_O_2_ acts as a chemotactic signal for leukocytes [141]. Importance of NOX enzymes activity for neutrophil recruitment was demonstrated in several other studies, for example, in the zebrafish heart regeneration model [58] and in *X. laevis* tadpole tail regeneration [71].

Further research revealed that activation of the Src-family kinase Lyn was essential for neutrophil recruitment to the wound. The authors argued that Lyn acted downstream of ROS: redox-active cysteine C466 of Lyn could be directly oxidized by inflammatory induced H_2_O_2_ thus promoting enzyme activation [271]. However, Jelcic et al. provided results that question the possibility of direct sensing of H_2_O_2_ by neutrophils via redox-sensitive proteins like Lyn kinase. The authors claim that redox-regulated proteins are less abundant in cells and less sensitive to ROS than antioxidant enzymes and thus would react with ROS only if the antioxidant system is exhausted. The authors estimated H_2_O_2_ concentration in the wounded fin of HyPer expressing fish and, according to their data, H_2_O_2_ concentrations sufficient to overwhelm the antioxidant capacity of cells were found within approximately 30 μm from the injury site whereas the neutrophils usually localize in blood vessels at a distance of 100–300 ϻm. Thus, direct sensing of H_2_O_2_ by redox-sensitive proteins other than antioxidant enzymes is not likely. Nevertheless, it would be of particular interest to measure the reactivity of Lyn kinase towards H_2_O_2_ and to compare it with that of the antioxidant enzymes [65].

Enyedi et al. discovered a mechanism of leukocyte recruitment after wounding by osmotic sensing. Zebrafish live in freshwater which is hypotonic to their interstitial fluids and cells. The authors carried out tail fin amputation in an isotonic solution unusual for zebrafish and observed that in these conditions attraction of neutrophils to the wound was significantly decreased although the dynamics of H_2_O_2_ production did not change. These results suggest that hypotonicity was sensed by the fish and is essential for leukocyte recruitment [68]. Cell swelling that occurs in a hypotonic solution activates a variety of proteins, including cytosolic lipase cPLA_2_ which is responsible for the release of arachidonic acid (AA), a well-known inflammation mediator [272]. Inhibition or knockdown of cPLA_2_ led to impaired neutrophil recruitment that confirmed its role in the hypotonically-induced inflammatory reaction. Likewise, the authors defined the neutrophil G-protein coupled receptor OXE-R that senses AA and its derivatives [68]. Thus, two major factors contribute to leukocyte recruitment to the injury site in zebrafish larvae: production of H_2_O_2_ by Duox and release of AA and its derivatives induced by exposure to hypotonic solution [68,141].

**Figure 3:**
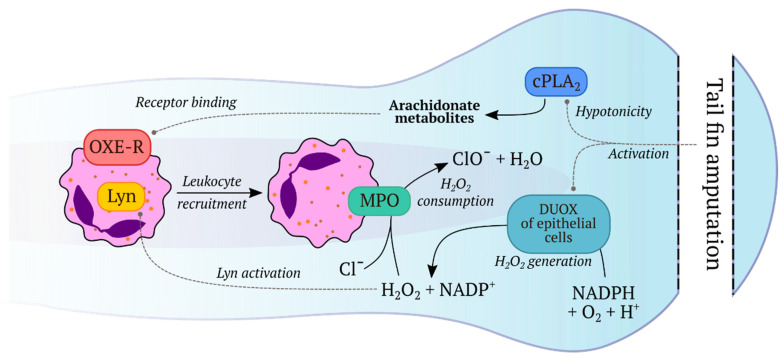
Inflammatory response in zebrafish larvae. Tail fin amputation leads to H_2_O_2_ production and release of arachidonate metabolite in the injured tissue. These compounds are sensed by leukocytes and play an essential role in their recruitment to the wound. H_2_O_2_ signal is dampened by arriving neutrophils as H_2_O_2_ is consumed in a reaction catalyzed by myeloperoxidase (MPO) [57,68,141,271].

The mechanism of ROS production after tail fin amputation in zebrafish was defined by Niethammer et al. [141], yet, it was unclear how H_2_O_2_ concentration decreases in the wounded area during the inflammatory response. Using HyPer expressing fish, Pase et al. showed that in mutants with inactive myeloperoxidase (MPO) in neutrophils, the H_2_O_2_ gradient spread further into the tail and did not decline over time in comparison to WT zebrafish. This phenotype could be rescued by populating mutant fish with normal neutrophils at the blastula stage. The authors concluded that the neutrophil enzyme MPO which converts H_2_O_2_ into hypochlorous acid is essential for H_2_O_2_ degradation [57]. The summary of the described processes is represented in Figure 3.

Deng et al. compared two inflammatory models, bacterial infection and tail fin amputation in *D. rerio*. According to their data, injection of the *Pseudomonas aeruginosa* PAK strain into the inner ear of larvae led to neutrophil accumulation in the inflammatory sites, though the HyPer signal did not significantly change. In larvae that were exposed to both bacterial infection and tail fin amputation, inhibition of Duox activity impaired neutrophil recruitment to the tail but not to the inner ear. This result suggests that during bacterial infection leukocytes are recruited by a mechanism independent of H_2_O_2_ production [142].

Recovery from injury requires not only leukocyte recruitment but also rapid wound closure. Gault et al. described the mechanism of osmotically induced wound closure in *D. rerio* larvae. It was demonstrated by microscopic observation that wound closure is inhibited in an isotonic solution. Additionally, HyPer expressing fish were subjected to tail fin amputation in isotonic medium and after wound-induced H_2_O_2_ diminished to the basal level, fish were transferred to isotonic or hypotonic medium supplemented with H_2_O_2_. In agreement with the previous observation, the barrier function of the epithelium was restored in hypotonic solution while in isotonic medium the fish tail remained permeable to H_2_O_2_ for several hours. Using the luciferin/luciferase system, the authors determined that isotonicity induced ATP release thus promoting basal epidermal cell migration and wound closure [67]. Thus, hypotonicity plays two major roles during mechanical injury in zebrafish: it facilitates rapid wound closure and is essential for leukocyte recruitment [67,68].

In another study, Xu et al. provided evidence that mitochondrial ROS were involved in wound closure in the *C. elegans* model. The authors used cpYFP to visualize mitochondrial superoxide flashes (mito-flashes) after wounding. Just after the manipulation, mito-flashes were suppressed for 70–100 s, then their amplitude and frequency significantly increased for several minutes [127]. It is important to note, there are debates about the nature of mito-flashes detected with cpYFP due to the doubts about cpYFP selectivity towards superoxide and the possible contribution of pH changes in its signal [273]. However, in study of Xu et al. pH was monitored with pHluorine and the fluorescence of the probe did not exhibit flash-like fluctuations. Treatment with pro-oxidants and mutations that elevate mitochondrial ROS facilitated wound closure in contrast to antioxidant treatment. The authors argued that a small GTPase RHO-1 is a potential target that senses mitochondrial ROS [127]. 

### 3.3. Regeneration

In two regeneration models, *D. rerio* partial ventricular resection of the heart [58] and *X. laevis* tadpole tail amputation, it was demonstrated that hydrogen peroxide serves as a signaling molecule and promotes tissue regeneration. In both cases, transgenic HyPer expressing animals were used [71,72]. By pharmacological or morpholino oligonucleotide inhibition, it was confirmed that H_2_O_2_ was produced by enzymes of the NOX family and accumulation of H_2_O_2_ preceded leukocyte recruitment similarly to the tail fin amputation model [58,71,141]. However, the temporal dynamic of H_2_O_2_ accumulation during regeneration was different. Amputation of the fin induced a rapid production of H_2_O_2_ that declined approximately 20 min post-amputation [141]. During regeneration the increase of H_2_O_2_ concentration occurred slower and remained elevated for several days until regeneration was almost complete: for the tadpole tail it took approximately seven days [71] and for the zebrafish heart—almost 30 days [58].

Additionally, the interplay between ROS and several signaling pathways during regeneration was revealed. ROS production during tadpole tail regeneration induced activation of Wnt/β-catenin signaling [71]. In another study, the authors detected hypoxia in regenerating tissue which promoted stabilization of the HIF-1ɑ transcription factor and hypoxia-induced signaling. The authors claimed that ROS accumulation did not contribute to the stabilization of HIF-1ɑ in their model [72]. During zebrafish heart regeneration, the activity of the MAP kinase cascade was prolonged due to destabilization of the redox-sensitive phosphatase Dusp6, a negative regulator of this pathway, by H_2_O_2_ [58].

Two other studies were devoted to liver tissue regeneration after partial hepatectomy in mice [146,147]. Under normal conditions, the liver maintains the ability to restore its volume after injury [274] though several health conditions may lead to impairment of this process. For example, regeneration of the liver is suppressed in aged mice [146] and mice with hepatic steatosis (fatty liver) [147]. Using the roGFP probe the authors detected oxidative stress in the liver of these animals after hepatectomy [146,147]. These mice also suffered from increased hepatic tissue injury by apoptosis [146] or necrosis [147] and insufficient activation of pro-survival Akt signaling [146,147]. The authors identified the misregulated genes in both cases, overexpression of p66Shc in aged mice [146] and down-regulation of p62/SQSTM1 in mice with steatotic liver [147] contributed to impaired liver regeneration. In these two models, ROS caused oxidative stress which inhibited the regeneration process [146,147].

ROS also serve as instructive signals for regeneration of nervous tissue. For instance, H_2_O_2_ is a beneficial signal that promotes peripheral axon growth and regeneration [275]. A recent study has reported how H_2_O_2_ interacts with peripheral nerves to regulate wound healing [63]. By using of the denervation strategy and multiple approaches for recording H_2_O_2_ levels in regenerating zebrafish fins, including live in vivo imaging of the HyPer sensor [100] expressed ubiquitously, two positive feedback loops were identified by which regenerating axons govern H_2_O_2_ levels that in turn evoke activation of Sonic Hedgehog signaling. The functioning of these feedback loops was found to be important for coordination of the regeneration process [63].

### 3.4. Neuroscience

The brain is the most metabolically active organ in the body. Although the human brain occupies, on average, only 2% of the body weight, it uses approximately 20% of the body’s energy [276,277]. A substantial portion of the energy used in the brain is associated with the firing of nerve cells. Neurons are the major consumers of energy in the brain. It is estimated that neurons use approximately 75%–80% of energy produced in the brain [278]. The energy supply in neurons relies mainly on mitochondrial oxidative phosphorylation. Neurons utilize their energy primarily for maintaining the resting membrane potential, for restoring ionic gradients across the plasma membrane following firing, and for supporting synthesis and recycling of neurotransmitters [277]. Even under normal conditions (no exogenous stimulation or a pathological process), brain activity requires a substantial energy supply and, therefore, is accompanied by a high rate of oxidative metabolism and, as a consequence, the unavoidable generation of intracellular ROS [277,279,280].

For a long time, ROS were thought to be noxious by-products that oxidize a variety of biomolecules, causing damage, and therefore the disruption of functional integrity of cellular components. However, it is becoming increasingly clear that ROS, primarily H_2_O_2_, and related redox signaling contribute to multiple physiological processes in both the central nervous system (CNS) and the peripheral nervous system (PNS). ROS and redox signaling are currently considered to play an essential integrative role in synaptic transmission, axonal guidance, brain development, peripheral nerve regeneration, nociceptive signal transmission, and the functioning and maintenance of adult neural stem cells [281,282,283,284,285,286,287,288,289,290]. For instance, pharmacological modulation of neuronal redox state by oxidizers or reducers alters glutamatergic and GABAergic responses to stimulation [291,292,293,294] and rearranges the actin cytoskeleton in neuronal growth cones [295]. Loss-of-function mutants of various enzymes involved in redox regulation in the CNS have impaired brain development in various species, causing multiple structural and functional abnormalities [296,297,298]. Disruption of redox signaling in peripheral neurons via the deletion of cyclic GMP-dependent protein kinase 1, a redox-dependent enzyme, impairs navigation of regenerating sciatic nerve axons after injury causing an enhancement of pain sensitivity in mice [282]. Knockout of NOX2, a ROS generating enzyme in the brain, decreases the number of proliferating neural progenitors and newborn neurons in the mouse hippocampus, showing the contribution of baseline ROS production to adult neurogenesis [299].

The brain is known to be highly vulnerable to perturbations in oxidative metabolism. This is primarily due to the high metabolic rate of the brain, the abundance of easily peroxidable polyunsaturated fatty acids within the membranes of nerve cells, the high content of iron that is known to catalyze ROS formation, and a relative deficit of antioxidant enzymes [300]. Oxidative stress and dysregulation of redox metabolism are currently thought to contribute to pathogenesis of cognitive aging, brain injury (trauma, stroke, radiation), neurodevelopmental (autism spectrum disorder, schizophrenia), psychiatric (bipolar disorder, major depression), and neurodegenerative (Alzheimer’s disease, Parkinson’s disease, Huntington’s disease, Rett syndrome, lateral amyotrophic sclerosis, multiple sclerosis) disorders [301,302,303,304,305,306,307,308,309,310,311,312,313,314,315,316,317]. 

For a long time, the contribution of ROS and redox signaling to physiologic and pathologic functioning of the CNS and the PNS was primarily studied using biochemical assays, morphological evaluation, and electrophysiological recordings. In general, studies in the field exploited the following strategies to address questions regarding the contribution of ROS and redox signaling to functioning of the CNS and the PNS under normal and pathological conditions. First, neural cell redox state is modulated by pharmacological compounds or by deletion or overexpression of the proteins and enzymes involved in redox signaling or energy metabolism followed by morphological evaluation and electrophysiological recordings [318]. Second, nervous tissue that is burdened either with a functional stress (evoked potentials, overstimulation, etc.) or with a pathological process (a model of neurological disorder) is biochemically assayed for oxidative stress markers (nitric oxide, hydrogen peroxide, lipid peroxidation, lactate dehydrogenase activity, ect.), levels of antioxidant enzymes (superoxide dismutase, glutathione reductase, glutathione peroxidase, etc.), and other endogenous redox molecules that counteract ROS (NADH/NAD^+^, NADPH/NADP^+^, GSH/GSSG, ect.). These approaches were extensively exploited in a variety of in vivo and in vitro experimental systems to address questions of how predetermined changes in neural cell redox state alter development, morphological organization, and functioning of the nervous tissue and what the contribution of ROS and redox signaling in brain functioning under normal and pathological conditions is. The major drawback of these approaches is a limited spatial and temporal resolution of the readouts. Therefore, they provide little information on how redox signaling is dynamically regulated by the functional activity of the CNS and PNS under normal and pathological conditions. Live redox imaging with GEFIs allows this gap in our knowledge regarding ROS and redox signaling in the brain to be filled.

GEFIs have been being widely utilized for live imaging redox state changes in neurons, glial cells, and neural progenitors in a variety of experimental in vivo (mice, flies, fish) and in vitro (cultured cells and brain slices) circumstances [62,319,320,321,322,323,324,325,326]. Several options exist to express redox GEFIs in neurons and glia. These are transfection, in utero electroporation, and viral transduction. Transgenic animals stably expressing redox GEFIs targeted to nervous cells have been created as well (Table 3) [59,60,61,62,63,64].

In vivo real-time imaging of neuronal firing, cell communication, cell signaling, and metabolism in the brain of most common model organisms, such as mice, zebrafish, and flies, is one of the fastest growing areas of neuroscience research. This is mainly due to the wide range of GEFIs available that illuminate multiple physiological processes in the living brain. Numerous papers have reported in vivo real-time imaging of neuronal firing [327], intracellular dynamics and release of neurotransmitters [328,329,330,331,332], turnover of the essential metabolites (glucose) [333], activity of second messenger systems (Ca^2+^, cAMP) [334,335], formation of ROS (H_2_O_2_) [150], and alterations in redox systems (NADH/NAD^+^, GSH/GSSG) [62,76]. Importantly, in vivo recording of physiological processes can be conducted in awake, freely behaving animals using custom-built or commercially available miniature microscopes and fiberscopes [332,333,335,336,337]. This approach enables a correlation between the behavior of an animal and functional activity of definite subpopulations of neurons and astrocytes to be found. Analysis of the correlation is crucial to understand how information is processed, stored, and retrieved in the brain and how the brain produces behavior and mental processes, adapts to the changing environment, and compensates its own disfunction when diseased.

Several recent studies have reported successful application of in vivo real-time imaging of redox GEFIs targeted to the CNS and the PNS to elucidate the contribution of ROS and redox signaling to energy metabolism, synaptic transmission, developmental and regeneration processes, and the pathogenesis of neurodegenerative disorders.

Brain function must quickly adapt to changing environmental circumstances to promote survival of the organism. Therefore, during brain excitation, neuronal energy metabolism must be readjusted to generate appropriate levels of energy. Energy supply during brain activation is one of key topics in neuroscience research. Recently, a hypothesis regarding the existence of an “astrocyte-neuron lactate shuttle” has undergone reevaluation [76]. It is well known that under normal conditions, the brain almost completely metabolizes glucose to CO_2_ to produce energy. When stimulated, the brain increases the consumption of glucose to a greater degree than the consumption of oxygen. This uncoupling of glucose and oxygen consumption was explained by a transient switch in energy metabolism to conversion of glucose to pyruvate and then to lactate. It was hypothesized that this switch occurs in astrocytes stimulated by synaptically released glutamate and that lactate produced and released by astrocytes serves as fuel for the energy metabolism of neighboring neurons. To evaluate this hypothesis, the Peredox sensor [107] sensitive to the NADH/NAD^+^ redox couple and the calcium sensor RCaMP1h [338] for monitoring neuronal activity were targeted either to granular cells of the hippocampal dentate gyrus for in vitro imaging in acute brain slices or to neurons of primary somatosensory cortex for in vivo imaging in awake mice [76]. Two-photon fluorescence lifetime imaging was used to record the occupancy of the Peredox sensor allowing for the direct measurement of the cytosolic NADH/NAD^+^ ratio. Both in vitro and in vivo examinations revealed that the neuronal NADH/NAD^+^ ratio alters after synaptic stimulation and that blockade of lactate uptake does not abolish this effect. These observations indicate that neuronal activity is not dependent on import of lactate to fuel energy metabolism, instead neurons enhance direct glucose consumption when they are stimulated [76].

Synaptic transmission, by which nerve cells communicate with each other, and the propagation of action potentials are highly energy expending processes that are reliant on mitochondria. Mitochondria are abundant in axons and synaptic terminals in all types of nerve cells in the CNS and the PNS. These two facts explain why the vast majority of recent studies were focused on multiparametric in vivo real-time imaging of redox signaling and ROS in the context of normal and pathological functioning of mitochondria. For instance, the contribution of mitochondrial Ca^2+^ and the NADH/NAD^+^ redox balance in the formation and functioning of ribbon synapses of hair cells in the lateral-line system of larval zebrafish was studied using of calcium and redox sensors [64]. The calcium sensors GCaMP6s [339], R-GECO1 [340], and GCaMP3 [341] were targeted to specific compartments of hair cells, the mitochondria, presynapse, and cytoplasm. The redox sensor REX-YFP [110] was targeted to the cytoplasm. Pharmacological blockade of Ca^2+^ channels in the plasma membrane and mitochondria combined with in vivo real-time imaging of calcium sensors and REX-YFP in immobilized larval zebrafish enabled identification of the NADH/NAD^+^ redox balance as a link that couples spontaneous presynaptic Ca^2+^ elevations and mitochondrial Ca^2+^ influx with the formation and functioning of the ribbon synapse [64].

Mitochondrial function during normal and pathological physiology of nerve cells was characterized by multiparametric in vivo imaging in *Thy1-mito-Grx1-roGFP2* transgenic mice [62]. In this mouse line, the Grx1-roGFP2 sensor [88] sensitive to the state of the glutathione redox system is targeted to the mitochondria of central and peripheral neurons. Multiparametric real-time imaging of mitochondrial function with the potential-sensitive fluorescent dye TMRM and GEFIs for Ca^2+^ (R-GECO1 [340]) and pH (SypHer [342]), both in vitro and in vivo, enabled the reconstruction of molecular events that are coupled to mitochondrial shape alterations under physiological (electrical stimulation) and pathological (nerve crush and chronic neurodegeneration) conditions. Similarly, mitochondrial health was evaluated in WT flies and mutants with progressive neurodegeneration and mitochondrial encephalomyopathy [61]. Real-time in vitro and in vivo imaging of the roGFP2 sensor [85] targeted to the mitochondria and cytoplasm revealed that, during aging, neuronal mitochondria in the mutant flies generate higher levels of ROS than WT controls, indicating involvement of redox dysregulation in the pathogenesis of degenerative processes.

In vivo live monitoring of roGFP2 [85] in axonal mitochondria in larval zebrafish revealed rapid elevation of mitochondrial matrix oxidation after axonal injury [133]. At the same time, expression of the slow Wallerian degeneration protein, a mutant mouse protein that delays axonal degeneration, or overexpression of the peroxisome proliferator-activated receptor gamma coactivator 1-alpha, a protein that is involved in mitochondrial biogenesis and ROS scavenging, diminished mitochondrial matrix oxidation induced by axonal injury. These observations indicate that the mitochondrial oxidation state after axonal injury correlates with the severity of subsequent axonal degeneration [133]. ROS generation and ATP production in mitochondria of myelinated axons within mouse peripheral nerves were evaluated under physiological (electrical nerve stimulation) and pathological (demyelination of axons) conditions using in vivo real-time imaging of H_2_O_2_-sensitive mito-roGFP-Orp1 [91] and ATP-sensitive ATeam [343] sensors targeted to mitochondria [150]. It was observed that, in resting axons, the levels of H_2_O_2_ and ATP in mitochondria residing in nodes of Ranvier were higher than in mitochondria of the internodal space, and that stimulated neuronal firing elevated levels of H_2_O_2_ and ATP in axonal mitochondria. Under demyelinating conditions, production of ATP in the mitochondria of the resting axons declined slightly, whereas generation of H_2_O_2_ increased significantly. These observations revealed that the link between ATP production and H_2_O_2_ generation probably disappears under pathologic conditions, thus offering a novel insight into the contribution of dysregulated mitochondrial redox metabolism to the pathogenesis of neurodegenerative disorders [150].

Intracranial recording of roGFP1 [85] targeted to neurons of the APP/PS1 transgenic mouse line, a mouse model of Alzheimer’s disease, revealed that mitochondria of “diseased” neurons, residing in a more oxidized state, had decreased membrane potential [143]. Notably, oxidative stress was higher in neuronal processes adjacent to plaques [321]. These observations indicate that oxidative stress accompanying Alzheimer’s disease may be linked, at least partially, with mitochondrial dysfunction [143].

Similarly, live in vivo imaging with roGFP2 sensor [85] revealed that neuronal mitochondria of larval flies with mutations in the *shawn* gene, a *Drosophila* homolog of the mitochondrial carrier proteins of the SLC25 family, exhibited a more oxidized state and decreased mitochondrial membrane potential [344]. Combined with aberrant neurotransmitter release in the mutants, this observation supports the contribution of mitochondrial disfunction to defects in synaptic transmission. 

Live in vivo imaging of ROS and redox signaling in the CNS and the PNS using GEFIs is burdened by the necessity of specific equipment (two-photon microscope, miniature microscope, fiberscope) and complicated surgical procedures (creating a cranial window, implantation of a platform for restraining the head under the microscope, implantation of the components of a miniature microscope or fiberscope) and other manipulations that require the appropriate skills. Therefore, if measurement of the dynamic alterations of ROS and redox state are not in the scope of an experiment, there is an option to directly measure ROS and redox state in fixed brain tissues. To perform this, a specific methodological strategy is necessary because common tissue processing and fixation with paraformaldehyde is always accompanied by unwanted and uncontrolled oxidation of redox GEFIs. This strategy exploits conservation of the redox state of GEFIs during tissue processing and fixation using N-ethylmaleimide alkylation of thiols [18]. H_2_O_2_ levels and glutathione redox state “snapfrozen” by the GEFIs redox state conservation have recently been assayed in fixed brain and spinal cord slices [105,319].

Apart from in vivo redox imaging with GEFIs targeted to neurons and astrocytes in live organisms, organotypic and acute brain slices and brain explants are frequently used to address similar questions. There are studies that exploited this approach in order to couple mitochondrial disfunction with oxidative stress in models of stroke [320,323,345], Parkinson’s disease [60,346,347], Alzheimer’s disease [348], and Rett syndrome [144,324]. Interestingly, several papers have reported real-time redox imaging of neurons and astrocytes during the course of oxygen-glucose deprivation and reperfusion in acute brain slices, a well-established in vitro model of stroke [320,323,345]. It is well known that uncontrolled ROS generation and redox system dysregulation underly the pathogeneses of stroke and brain hypoxic/ischemic injury. Obviously, real-time imaging of GEFIs offers a unique opportunity to trace redox dynamics during the course of stroke. However, live in vivo imaging of alterations in the redox state during middle cerebral arteria occlusion, an in vivo model of stroke, has not been attempted yet. In vivo real-time imaging of redox dynamics during middle cerebral arteria occlusion is extremely desirable because the model of oxygen-glucose deprivation and reperfusion in acute brain slices fails to replicate many aspects of stroke. 

To date, a tiny number of papers have reported in vivo brain redox imaging with GEFIs. However, this is a quickly growing research area because the palette of GEFIs is intermittently replenished with novel sensors or sensors with improved characteristics, thus offering new opportunities for multiparametric in vivo brain redox imaging. Moreover, multiple optical approaches for functional brain imaging, including those that are compatible with behavioral experiments in awake, freely moving animals, provide great flexibility in experimental design.

### 3.5. Cancer

Oxidative stress, known to be involved into pathogenesis of numerous human diseases, is implicated in cancer as well. Cancer cells undergo continuous proliferation and, therefore, are metabolically active. Elevated levels of ROS and remodeled energy metabolism based on aerobic glycolysis (the Warburg effect) are hallmarks of multiple cancer cell lines [349,350,351,352]. However, the role of ROS in tumorigenesis remains ambiguous [353,354]. One line of evidence has argued that ROS promotes tumorigenesis. It is currently believed that overproduction of ROS in the cell may underly nucleic acid damage, leading to the appearance of tumorigenic mutations and promoting genome instability [355,356,357]. Moreover, it has been shown that elevated ROS levels mediate remodeling of the normal transcriptional program, causing overexpression of proto-oncogenes or inactivation of tumor suppressor genes, thus, contributing to cell transformation [358,359]. Additionally, the ectopic or induced expression of oncogenes is almost always accompanied by elevated ROS levels [360,361,362]. ROS is also considered to be implicated in migration and invasion of cancer cells [363]. For instance, elevated ROS levels in tumor cells were found to induce mutations in the mitochondrial DNA, thus altering tumor cell behavior and metastatic potential [364], or to modulate signaling pathways implicated in epithelial–mesenchymal transition of cancer cells [365]. However, accumulating experimental evidence indicate that suppression of ROS in tumor cells by genetic manipulations or application of antioxidants seems to be non-beneficial.

Suppression of ROS in tumor cells can promote tumorigenesis [366,367]. Application of the antioxidants vitamin A and N-acetylcysteine had no beneficial effect on head and neck cancer and lung cancer in a clinical trial as well [368]. Another line of evidence states that ROS prevents cancer. Although most tumor cell lines exhibit elevated levels of ROS, they remain vulnerable to high levels of ROS, and they heavily rely on endogenous antioxidant defense systems [353,354,369]. An imbalance between ROS production and detoxification of ROS by endogenous antioxidant defense systems may lead to cancer cell cycle arrest, senescence, and apoptosis [353,354,369]. Excessive ROS production almost always accompanies chemotherapy and radiotherapy. This excessive ROS induces cancer cell injury, thus contributing to the beneficial effects of cancer therapy. On the other hand, overproduction of ROS and prolonged oxidative stress during the cancer treatment can activate multiple defensive mechanisms, enabling the development of resistance to cancer therapy [370,371]. In sum, ROS and the redox state are currently thought to play significant roles in the modulation of multiple signaling pathways to adapt cancer cell behavior during the progression of an untreated tumor or when exposed to cancer therapy. However, the ambiguity of the contribution of ROS to tumorigenesis and the controversy of the effect of antioxidants on tumor progression represent the challenging problem of how tumor cell redox state should be manipulated to promote effective and safe cancer therapy.

The ambiguity of the contribution of ROS to tumorigenesis seems to originate from the multifactorial nature of tumors and their complexity. Tumors have a heterogeneous cell composition and commonly consist of both cancer cells with a different degree of malignancy and different types of normal cells. This heterogeneity underlies the appearance of a complex redox landscapes within tumors. Moreover, these landscapes vary along with tumor progression. Therefore, to better understand how ROS and cell redox state contribute to cell transformation, tumor growth and spread, the beneficial effects of cancer therapy, and the appearance of resistance to cancer therapy, we need to dynamically measure ROS and redox state with a high spatial and temporal resolution. Obviously, in vivo live imaging of ROS and redox state in tumors enables more valuable readouts regarding redox landscapes within tumors in comparison to redox imaging of cultured cancer cells that do not reproduce the complex cell interactions observed in intact tumors. Additionally, in vivo monitoring of the redox response in tumors to therapeutic intervention in animal tumor models will be useful for screening novel anticancer drugs in terms of their effectiveness, safety, and risk of acquired resistance to cancer therapy.

Despite the conspicuous utility of in vivo redox imaging using GEFIs for cancer research, a tiny number of manuscripts have reported the application of this approach to address questions regarding the roles of ROS, redox signaling, and energy metabolism in tumor progression and cancer treatment. For instance, a recent study revealed that the metabolic properties pre-determine the fates of leukemia-initiating cells in an MLL-AF9-induced murine acute myeloid leukemia model [148]. In this study, Lin^−^ mouse fetal liver cells were transduced with viral vectors to express the MLL-AF9 fusion construct necessary to induce acute myeloid leukemia and the SoNar sensor which is sensitive to the NADH/NAD^+^ ratio, an indicator of the rates of glycolysis and mitochondrial respiration [111,149]. The transduced Lin^−^ cells were then transplanted into lethally irradiated recipient mice. Both in vitro and in vivo examinations identified SoNar-high and SoNar-low subsets within a population of acute myeloid leukemia cells. The SoNar-high subset of acute myeloid leukemia cells was found to be enriched for leukemia-initiating cells which tend to reside in endosteal niches and undergo symmetric divisions supporting leukemogenic activities [148]. At the same time, cells in the SoNar-low subset preferred to localize closer to vascular niches and exhibited equal frequencies of symmetric and asymmetric divisions [148]. 

Yet another study has identified a redox signaling mechanism that underlies epithelial cell invasion in the intestine of zebrafish meltdown (*mlt*) mutants [135]. Cancer cell invasion into the surrounding tissue is an event that contributes to the formation of metastasis. This process is a hallmark of malignant tumors. Disruption of intestinal architecture in zebrafish *mlt* mutants mimics events that observed during cancer cell invasion. Particularly, cells of the intestine epithelium in zebrafish *mlt* mutants form membrane protrusions that resemble the invadopodia observed in invasive cancer cells. These membrane protrusions contain the metalloproteinase Mmp-14 that is necessary for degradation of the basement membrane and for subsequent epithelial cell invasion into parenchyma. Using this model system and live in vivo imaging of the glutathione redox couple-sensitive sensor Grx1-roGFP2 [88] targeted to the intestine epithelium, the authors identified a ROS-mediated feedback signaling loop that enhances the contractile tone of adjacent smooth muscle that in turn amplifies the production of ROS in epithelial cells, contributing their invasion [135]. 

Tumor initiation, maintenance, and progression do not solely depend on increased oncogene activity due to a mutation in a proto-oncogene but also on normal cell functions. This phenomenon is referred as non-oncogenic addiction. Many proteins supporting normal cell functions have been identified to contribute non-oncogenic addiction and are currently considered potential targets for cancer therapy [372]. A recent study identified redox signaling as a key factor that determines the sensitivity of normal and cancer cells to inhibition of a non-oncogenic addiction enzyme mutT homologue (MTH1) both in vitro (in osteosarcoma cells) and in vivo (in zebrafish embryo) [136]. Live in vivo imaging of Grx1-roGFP2 [88] targeted to the cytoplasm and mitochondria in zebrafish embryos revealed that creating a condition resembling hypoxia via treatment with the hydroxylase inhibitor dimethyloxalylglycine evoked marked oxidation of the glutathione pool [136]. This shift in redox state was accompanied by a significant decrease in the survival of zebrafish embryos after MTH1 inhibition. At the same time, antioxidant administration in this model system protected zebrafish embryos against MTH1 inhibition [136]. Similarly, antioxidant treatment increased survival of U2OS osteosarcoma cells after pharmacological inhibition of MTH1 [136]. This study provided experimental data on how redox landscapes can modulate the action of anticancer drugs. 

In vivo optical imaging of cancer cells has become a powerful toolset to address numerous questions regarding tumor progression, expansion, and therapy. This toolset enables experimenters to detect cell signaling and track cell migration and invasion by illuminating transcriptional activity and specific cell types by fluorescent and bioluminescent reporters (FPs and luciferase) targeted to cancer cells, trace the cancer cell cycle using a fluorescent ubiquitination-based cell cycle indicator (Fucci), visualize angiogenesis by marking blood vessels with a fluorescent dye, and evaluate cancer cell metabolism by recording endogenous fluorescence of the metabolic cofactors NAD(H), FAD(H), and NADP(H) (reviewed in [373,374]). In vivo redox imaging by using GEFIs provides live monitoring of molecular events with superior specificity and a high spatiotemporal resolution, thus extending the opportunities of the existing toolset.

Many cancer cells are characterized by genome instability and have an increased tendency to acquire mutations, which can contribute to tumor progression and increase the degree of malignancy. Mutations may appear in genes encoding proteins involved in energy metabolism and redox regulation. Therefore, accumulation of mutations in these genes contributes to the re-programing of cell metabolism, thus linking genetics and metabolism of cancer cells. The recent introduction of several CRISPR/Cas9-based screening approaches opened new avenues for studying the genetic basis of changes in cancer cell metabolism. Particularly, the application of so called combinatorial CRISPR/Cas9 screening [375] where phenotypes of pairwise gene-knockouts are evaluated has allowed for the identification of gene interactions essential to carbohydrate metabolism in several cancer cell lines [376]. In yet another study, specific point mutations in key redox enzymes that were introduced using CRISPR/Cas9 system were found to contribute to oxidative stress and the acquisition of chemoresistance in epithelial ovarian cancer [377]. The application of CRISPR/Cas9 for in vivo studies in the field of cancer research is limited by insufficient editing efficiency. Therefore, ex vivo genome editing of cancer cells followed by transplantation into animals can serve as a reliable model for the identification of genes essential for tumor progression, metastasis, and therapy [378]. In vivo real-time redox imaging of tumors derived from cancer cells that have undergone genome editing using the CRISPR/Cas9 technology will enable new insights into the genetic mechanisms underlying modified redox metabolism in cancer cells and the creation of model systems for the evaluation of novel anticancer drugs.

In sum, in vivo redox imaging of tumors using GEFIs is still in its infancy. At that same time, this approach is of great promise. Numerous important questions regarding the roles of ROS and redox metabolism in tumor progression and expansion, as well as the appearance of chemoresistance can only be addressed using this approach. 

### 3.6. Some Other Interesting Examples in Mammals

Recently, redox GEFIs were applied in diabetes research. Reissaus et al. developed a novel platform for studying β-cells in pancreatic islets in vivo. Their approach combined virally induced expression of biosensors in β- cells of a living animal and intravital microscopy. The use of abdominal imaging windows allowed repeated observation of the same mouse for up to several weeks. The authors demonstrated the efficiency of their method in a pharmacologically induced model of diabetes. Streptozotocin injections induced oxidation of the cytoplasm of β-cells that was measured by the Grx1-roGFP2 probe, however, the redox state of the cells returned to a normal level approximately two weeks after the treatment [55].

Several research articles have been devoted to the study of diabetic nephropathy, a common complication of diabetes. At present, mitochondrial dysfunction is thought to contribute to the development of this pathology [145]. Studies have been carried out on db/db mice—a genetic model of diabetes. With the roGFP2 biosensor targeted to the mitochondrial matrix, it was revealed that the mitochondria of diabetic mice were more oxidized than in healthy animals [20,21]. In addition, in diabetic milieu mitochondria underwent excessive fission [21]. Mitochondrial fission is regulated in part by the Drp1 protein, a member of the dynamin GTPase superfamily [379]. Previously it was revealed that hyperglycemia-induced mitochondrial fission in the kidneys was promoted by phosphorylation of Drp1 protein at the serine 600 residue [380]. Galvan et al. obtained transgenic diabetic mice with a serine 600 to alanine mutation in Drp1 to exclude the possibility of phosphorylation at this site and to study the functional consequences of Drp1 phosphorylation in the progression of diabetic nephropathy. Mutant diabetic mice displayed improvement in several biochemical and histological parameters, a decrease in mitochondrial fission, and normalization of the redox state of mitochondria in comparison with control diabetic mice. The authors assumed that the Drp1 protein may be a potential therapeutic target in the treatment of diabetic nephropathy [21].

Several experimental articles have been devoted to the study of light-induced ROS production in mouse skin. It is well established that UV radiation contributes to skin aging and cancer: UVB (280–315 mn) causes direct DNA damage while UVA (315–400 nm) damages DNA indirectly by promoting the formation of ROS [381]. To monitor oxidative changes induced by UVA light in vivo Wolf et al. obtained a transgenic line of hairless albino mice with roGFP1 expression in the mitochondria or cytoplasm of keratinocytes. It was demonstrated that UVA irradiation caused oxidation in mouse skin in mitochondria only [54]. However, in a study from Nakashima et al. UVA of higher intensity induced oxidation of the cytoplasm as well [53]. The authors also examined the effect of visible and infra-red light on mouse skin. It was revealed that blue light can induce oxidative stress in the mitochondria but not in the cytoplasm of keratinocytes even at high intensities while other types of radiation tested caused no significant effect. In addition, UVA and blue light induced oxidation of the roGFP1 biosensor in mitochondria in cultured human keratinocytes (HaCaT cell line). This data suggests that blue light may contribute to skin damage similar to UVA, though to a lesser extent: per photon efficacy of blue light-induced oxidative stress in mitochondria was 68% and 25% of that of UVA in mouse skin and in HaCat cells, respectively [53].

## 4. Redox Biosensors in Plants

Abundant research continues to demonstrate the importance of ROS in a wide variety of biological processes [382]. In plants, ROS play a crucial role in growth, development, and pathogen defense [383].

### 4.1. Redox Metabolism of Chloroplasts

**Figure 4:**
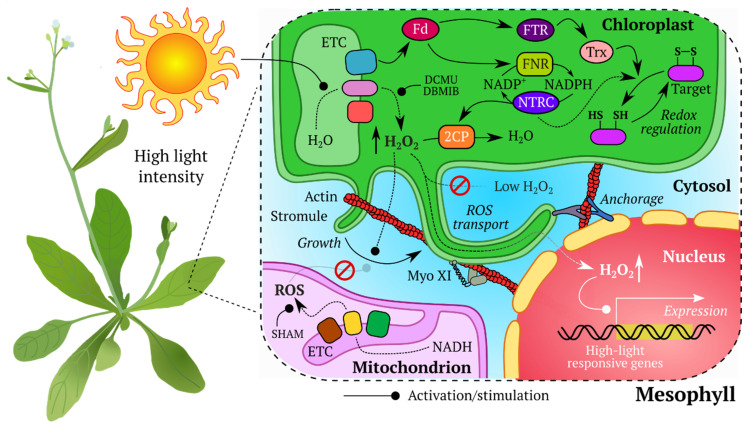
Schematic illustration of redox events during light-inducible reaction in photosynthetic leaves of *A. thaliana*. Plants sense light with the pigments of the photosynthetic electron transport chain (ETC) in the chloroplast or with photoreceptors. Chloroplasts generate stromules in response to the changes in the internal chloroplast redox status in a pathway regulated by the chloroplast NADPH-dependent thioredoxin reductase, NTRC. Stromules deliver H_2_O_2_ directly to the nucleus bypassing the cytosol. Chloroplast-sourced H_2_O_2_ in the nucleus may act as a signal to induce gene expression, which facilitates the acclimation of cells to high light intensities [163,187,188]. Abbreviations: 2CP—2-Cys peroxiredoxin; Myo—myosin; FTR—(Fd)-dependent Trx reductase; DBMIB—2,5-dibromo-6-isopropyl-3-methyl-1,4-benzoquinone; FD—ferredoxin; Trx—thioredoxins; DCMU—3-(3,4-dichlorophenyl)-1,1-dimethylurea; FNR—ferredoxin-NADP^+^ reductase; SHAM—salicylhydroxamic acid.

Plants experience the largest fluctuations in electron flux and ROS production due to differences in sunlight intensity during the day or season. Like other organisms, moderate levels of ROS modulate the plant metabolism and photosynthetic activity. At the same time, high concentrations of ROS are detrimental to plant cells and tissues [384]. It is well known that chloroplasts are organelles with a high rate of electron flow, which provides an additional source of oxidant production in plants. For example, it was shown on photosynthetic leaves of *Arabidopsis thaliana* that the photosynthetic mechanism changes in cells exposed to high light intensities, provoking a large increase in the level of H_2_O_2_ in chloroplasts and peroxisomes [182]. According to one hypothesis, H_2_O_2_ plays an important role as a signaling molecule that facilitates to the acclimation of cells under high light intensities [182]. At the same time, a mechanism was shown, where hydrogen peroxide gets to the nucleus directly from chloroplasts, bypassing the cytosol, which makes it possible to directly regulate gene expression through a photosynthetic signal [163]. A similar mechanism of signaling is necessary for the acclimation of photosynthetic cells to light intensity fluctuations [163]. A model has been proposed which promotes the formation of stromules, stroma-filled tubules that extend from all types of plastids, in the leaves by increasing ROS in the chloroplasts and increasing the level of sucrose in cells with leucoplasts [163]. To prove this idea, the redox status of the chloroplast was measured using roGFP2 in *A. thaliana* cotyledons [163]. Later Exposito-Rodriguez et al., using HyPer2, a hydrogen peroxide sensor, concluded that chloroplast-sourced H_2_O_2_ is transferred to the nucleus where it may act as a signal to induce high light intensity responsive gene expression (Figure 4) [188]. 

Meanwhile, it was discovered that such inter-organellar communication is vital for successful plant innate immune responses [187]. During the immune response, the concentration of H_2_O_2_ increased in the nucleus at the connection sites of stromules and nuclei [187]. Caplan et al. showed that chloroplasts in *Arabidopsis* dynamically change their morphology by sending out stromule extensions during the defense response [187]. Moreover, they showed a gradient of H_2_O_2_ emanating from a chloroplast associated with the nucleus and accumulation of chloroplast-generated H_2_O_2_ in nuclei using the chloroplast-targeted HyPer sensor. Their study described a model where stromules may be involved in retrograde chloroplast-to-nuclear signaling [187]. 

### 4.2. Redox Metabolism of Peroxisomes

If antioxidant systems cannot cope with elevated H_2_O_2_ levels or the production of ROS is not balanced, oxidative stress may occur [385]. The main sources of ROS in photosynthetic organisms are chloroplasts and peroxisomes, in contrast to non-photosynthetic organisms, where the main source of ROS production are mitochondria [386]. There is a wide variety of peroxisomes classified by morphology and function like glyoxysomes, leaf peroxisomes, and root peroxisomes [387]. ROS, such as H_2_O_2_, are abundant in peroxisomes because acyl-CoA oxidases [388] and glycolate oxidase [389], which play crucial roles in the β-oxidation and glycolate pathways, respectively, produce hydrogen peroxide in their reactions. In addition, peroxisomes possess systems for scavenging hydrogen peroxide [390]. Costa et al. using HyPer expressed in tobacco and *Arabidopsis* plants, demonstrated that intraperoxisomal Ca^2+^ rise stimulates catalase activity for increased peroxisomal H_2_O_2_ scavenging efficiency [180].

The importance of catalase in maintaining proper metabolism of peroxisomes was demonstrated with the help of roGFP2 [162]. Shibata et al. showed that the excess amount of H_2_O_2_ that is formed as a result of inactivated catalase leads to peroxisome aggregation [162]. These organelles can form special structures called peroxules, which are thin protrusions from spherical peroxisomes produced under low levels of ROS stress [184]. A study using peroxisome-targeted HyPer and a mitochondrial targeted GFP probe expressed in *Arabidopsis* plants, revealed sustained interactions between peroxules and small, spherical mitochondria [184]. The model suggests peroxules act as interaction platforms for ROS-distressed mitochondria that may release membrane proteins and fission factors, which increases their availability for peroxisomes, leading to active proliferation, enhancing plant cell protection [184]. 

### 4.3. Stress Conditions

Plants are constantly exposed to environmental challenges such as salinity, drought, pathogen attack, and biotic factors such as herbivory [391]. In order to combat these abiotic and biotic threats, plants have evolved complex signaling networks to ensure their survival. One of them is ROS, which help the plant to adapt to various stress conditions [383]. For example, to monitor the overall redox status in roots in non-stressed and in saline environments Jiang et al. used *Arabidopsis* seedlings, expressing a redox-sensitive reporter (roGFP) [152]. This study shows that the signal of the sensor (roGFP) varied in different root regions in response to salt stress both spatially and temporally. Moreover, the differing responses to varying levels of salt point to mechanisms used by roots to adjust to saline soils [152].

Water is a limiting resource for plant growth and development, which is why it is very important for plants to have constant access to this resource, otherwise they are exposed to water stress. Understanding the molecular mechanisms of water stress can help us cope with the effects of adverse conditions such as drought. For example, Jubany-Mari et al. using cytosolic roGFP1 demonstrated changes in redox potential of the cytosol to more oxidized in plants subjected to water-stress, with subsequent reduction, followed by re-watering [155]. Interestingly, the re-watering was paralleled by a return of water stress, redox potential, and ascorbate to initial values, showing the reversibility of water stress and its consequences [155]. 

#### Glutathione Metabolism during Stress

The potentially damaging end-products of aerobic energy metabolism, ROS, are powerful signaling components linking growth, metabolism, and defense responses in cells [392]. At the same time, the key component that maintains the redox status of the plant cell is the glutathione pool [393]. It is known that the redox state of plant mitochondria, plastids and cytosol is highly reduced because of NADPH-dependent glutathione reductase [159]. Glutathione reductase catalyzes the reduction of GSSG into GSH [394]. In contrast with other organisms, plant genomes encode two isoforms of glutathione reductase: GR1 and GR2, which have different importance for plant development [395]. Yu et al., using Grx1-roGFP2, demonstrated that proper activity of plastid-localized GR2, is essential for root growth and root apical meristem maintenance. [170]. Another study explicated the role of GR1 in plants with the help of glutathione-specific redox-sensitive GFP, which was used for dynamic measurement of *E_GSH_* in the cytosol [168]. Marty et al. demonstrated that GR1 mutants survive and even show slower dynamic reduction in GSSG compared to WT, subject to a normally functioning thioredoxin reductase A, which is the backup system for GR1 [168]. Moreover, glutathione plays a crucial role during abiotic stress in plants. Cheng et al. reported that endogenously increased GSH, which was measured by Grx-roGFP2, conferred tolerance to drought and salt stress in *Arabidopsis* [171]. 

Glutathione has great importance in the signaling processes underlying essential defense responses in plants. Previously in a study carried out by Dubreuil-Maurizi et al. it was shown that *Arabidopsis* phytoalexin-deficient mutants are more vulnerable to a large number of pathogens [169]. Moreover, these mutants possess a more oxidized glutamate-cysteine ligase protein, which is the first enzyme of the GSH biosynthetic pathway, encoded by a gene containing a phytoalexin-deficient mutation [169]. Thus, using Grx1-roGFP2, Dubreuil-Maurizi et al. demonstrated that due to a lower level of glutathione in mutants there is more oxidized glutamate-cysteine ligase protein in the phytoalexin-deficient mutant which makes plants more defenseless [169].

### 4.4. Growth and Development

#### 4.4.1. Root Growth

The responses of cells to cellular oxidation due to abiotic stress or the action of defense phytohormones depend on cell identity. Using the roGFP1 probe, Jiang et al. demonstrated that the redox potentials of various regions of *Arabidopsis* roots differ significantly [154]. The redox status, in turn, depends on glutathione transferases (AtGSTF8 and AtGSTU19), which are involved in the maintenance of root redox status affecting meristem size and salt stress sensitivity [175]. According to results obtained by Horvath et al., the redox status of untreated *Atgstu19* mutant roots was more oxidized than that of the WT, and the size of the mutant’s meristem proved to be shorter compared than the WT [175]. Redox potential, measured by roGFP2, showed the biggest differences in the proximal meristem of the roots. Treatment with a high concentration of NaCl resulted in a more oxidized redox state in all studied zones of the roots of the investigated genotypes, but the highest redox potential values were detected in the transition zone of the *Atgstu19* mutant [175].

Furthermore, it is known that micromolar concentrations of aluminum significantly limit plant growth in acidic soils by disrupting root hair development, although the precise mechanism by which aluminum inhibits root elongation has not yet been established [396]. Hernández-Barrera et al., found during experiments, using HyPer in *Arabidopsis*, that during aluminum exposure, cessation of root growth, mainly in the elongation zone, occurs due to a decrease in H_2_O_2_ levels [183]. Thus, an increased H_2_O_2_ level is a crucial and tightly regulated event during optimal root growth in plants [183].

#### 4.4.2. Pollen Germination

Together with nitric oxide, ROS and GSH are key regulators of plant development [397], in particular, development of flowers and pollen germination [398]. In a study by García-Quirós et.al roGFP-expressing lines were used to estimate the redox state of the nuclei and cytosol of flower and pollen cells, confirming the essential role of a highly reduced glutathione pool on pollen germination and tube growth [166]. In another study Boisson-Dernier et al., using HyPer, showed that the tip-focused Ca^2+^ gradient, which is maintained by NOX enzymes producing tip-localized H_2_O_2_, is essential for pollen tube growth [181].

### 4.5. Redox Processes Regulating Stomata Function

Stomata are formed by pairs of specialized guard cells that sense and integrate multiple stimuli such as light, CO_2_, humidity, and hormones, to modulate stomatal pore size [399]. Therefore, stomata play a crucial role in controlling transpiration and the plant’s water status. Stomata closure is tightly regulated by various enzymatic systems including NOX [400], phospholipase D [401] and gasotransmitters (NO) [402]. Recently, H_2_S has been discovered as another gasotransmitter regulating stomatal closure [403]. Scuffi et al., using the biosensors roGFP2-Orp and Grx1-roGFP2 carried out a study, revealing that H_2_S stimulates H_2_O_2_ production in *Arabidopsis* guard cells, which leads to further closure of stomata [174].

Furthermore, some pathogens can enter the plant through the stomata (Rodrigues et al. 2017). It is known that plants have the capacity to close their stomata after perception of pathogen-associated molecular patterns or damaged associated molecular patterns [404]. A study carried out by Rodrigues et al. using HyPer revealed that stromata closure occurs due to the *Arabidopsis* plasma membrane aquaporin PIP2;1, which is also required for intracellular accumulation of hydrogen peroxide after pathogen-associated molecular pattern *flg22* treatments [185]. As a result, Rodrigues et al. proposed a model where flg22 activates the plasma membrane aquaporin PIP2;1 in order to contribute to the transport of both water and H_2_O_2_ and promote stomatal closure [185].

### 4.6. Interaction of Plants and Phytopathogens

Plants attacked by pathogens react by producing ROS in a defense reaction called the oxidative burst [405]. The impact of ROS on necrotrophic fungi is still unclear and sometimes even contradictory [406]. Although several reports have shown that some fungi require effective ROS-scavenging machinery to overcome the plant’s oxidative burst, others have demonstrated that fungi are completely unaffected by plant-derived ROS [407,408]. In addition, ROS signaling has been proposed to be important for several differentiation processes in filamentous fungi, such as conidiation and the formation of appressoria, sclerotia or fruiting bodies [409]. For example, in the rice blast fungus, *Magnaporthe oryzae*, H_2_O_2_ plays an important role in the development of special structures called appressoria that are used to infect host plants [196]. Notably, the production of hydrogen peroxide is a prerequisite for appressoria formation. Improper work of NOX enzymes interferes with appressorial development [196]. Using the HyPer-2 indicator Mentges and Bormann analyzed ROS dynamics in mycelia and observed higher H_2_O_2_-levels in infection cushions of *Fusarium graminearum* in the early infection stages on wheat [194]. Mentges and Bormann demonstrated that developmental processes like septation and development of infection structure are accompanied by an increase in intracellular H_2_O_2_ levels [194]. Another crucial parameter that changes upon penetration by infecting hyphae is the cellular glutathione pool [191]. Heller et al. observed a difference in the glutathione pool in *Botrytis cinerea* between appressoria-like structures and infecting hyphae. They show that the glutathione pool is more reduced in the presence of infecting hyphae and more oxidized around appressoria-like structures [191]. 

Moreover, the redox state of the different fungal compartments is affected by stress-inducing agents in different ways. Surprisingly, every stress signal seems to be transduced into a changed redox potential. Marshall et al., expressing the biosensor roGFP2 in different cellular compartments of *B. cinerea*, showed that the redox state of filamentous fungus gradually shifted from the cytosol to the mitochondria and the ER as the most oxidized compartment [192]. The present evidence suggests that different external stressors influence the redox states of the three compartments. They are first recognized and transmitted in the cytosol and afterwards in the ER or the IMS. The data presented in this study underline the close association of ROS, Ca^2+^ signaling, and cell wall stress [192]. 

To protect against the penetration of pathogens, plants have developed their own defense mechanisms, which include the production of ROS [405]. However, this response, called an oxidative burst, can promote infection by necrotrophic fungi that take advantage of the host’s response, feeding on dead plant material [410]. Since plants have ROS defense mechanisms, all phytopathogens try to adapt to such conditions, developing their own protective mechanisms against oxidative stress. Balanced production, decomposition and use of ROS for their own needs is the most important strategy for the survival and maintenance of activity by pathogenic fungi [410]. For example, data obtained using Grx1-roGFP2 established that fungi require effective antioxidant defense systems to cope with oxidative stress [195]. Experiments carried out by Samalova et al. showed that *M. oryzae* regulates the redox potential of the glutathione pool well and, accordingly, has sufficient antioxidant activity to cope with the plant’s nonspecific response to fungal infection [195]. Thus, plants need to develop additional methods of protection to counteract infection with this fungus. 

Another way to counteract plant defense reactions is the expression of transcription factors which increase the resistance of fungi to adverse conditions. For instance, *Cochliobolus heterostrophus*, a fungal plant pathogen that causes southern corn leaf blight in maize, produces the redox-sensitive transcription factor ChAP1, which is required for oxidative stress tolerance [193]. In order to confirm that ChAP1 maintains redox homeostasis in the pathogen during oxidative stress, HyPer was expressed in *C. heterostrophus* to detect changes in H_2_O_2_ levels [193]. The study showed that *chap1* mutants are actually slowly recovering the redox homeostasis compared with the wild type on exposure to H_2_O_2_ [193]. 

### 4.7. Symbiosis

Not all microorganisms are pathogenic towards plants, many of them can stably coexist forming a widespread network of relationships between macro and microorganisms living on the plant surface, called the phytobiome [411]. Nowadays, this topic deserves special attention, since it was found that the proper interaction between plants and macro and microorganisms depends on the reduction-oxidation environment of the phytobiome [256]. Liu et al. discovered a plant–microbe redox interface which was expressed in the epiphytic bacterium *Pantoea eucalypti 299R* (Pe299R/roGFP2), using roGFP2. The Pe299R/roGFP2 reporter rapidly assesses differences in redox microenvironments and provides a noninvasive tool that may be very useful for studying symbiotic interactions [256].

Using another molecular genetic tool - HyPer, which was expressed in *Medicago truncatula* plants inoculated with the *Sinorhizobium meliloti* DsRed strain, it was found that H_2_O_2_ is involved in the signaling processes during the establishment of legume-Rhizobium symbioses [186]. As a result of complex molecular interactions between the plant and the symbiont, root nodules, in which nitrogen fixation occurs, are formed. NOX enzymatic activity appears to play an important role in H_2_O_2_ generation during the process of symbiosis formation [412], and HyPer probes have been used for imaging H_2_O_2_ accumulation in nodules [186].

### 4.8. Analysis of the Topology of Transmembrane Proteins

The secretory pathway is an essential system of functionally interconnected organelles. Approximately one-third of all newly translated proteins in a eukaryotic cell are translocated into the ER to be delivered to their final destination along secretory pathway routes [189]. A challenging task for molecular analyses of membrane proteins in the secretory pathway is to determine their topology relative to the membranes, in which they are embedded. Using ratiometric imaging of roGFP tags fused to proteins, Branch et al. developed a method for analysis of the topology of transmembrane proteins in the secretory pathway, which relies on the difference in the *E_GSH_* across the ER membrane, which is sensed by roGFP [189]. The measurement results in ratiometric images which provide direct information about the orientation of roGFP relative to the membrane, since roGFP fluorescence changes with changes in the redox potential of glutathione across the ER membrane. To test the effectiveness of the method, Branch et al. measured an oxidized roGFP inside the ER lumen and reduced roGFP on the cytosolic side of the membrane for both N- and C-terminal fusions of single and multi-spanning membrane proteins [189]. This method allows for the topological analysis of transmembrane proteins in the secretory pathway, determining their position and possible functions in a particular organism.

## 5. Redox Biosensors in Microorganisms

### 5.1. Oxidative Stress Caused by External Factors and Genetic Landscape

#### 5.1.1. Oxidative Stress in Bacteria

An obvious implementation of redox sensors is imaging redox stress induced by toxic compounds, genetic landscape and/or cultivation conditions. These data allow the exact molecular mechanisms of action of oxidants and their interplay with cellular pathways, in particular, with antioxidant systems to be investigated. Thus, experiments using genetically encoded tools can clarify the functions of poorly studied redox metabolism related genes and their protein products. *M. tuberculosis* (Mtb), a socially significant pathogen which causes about 10 million incidents of tuberculosis annually [413], is represented by seven distinct lineages found in different global regions [414]. Deciphering their metabolic features including the ability to resist oxidative damage is of high practical importance as it paves the way for drug development. In their work using Mrx1-roGFP2 probe for *E_MSH_* measurements, Arumugam et al. addressed the functions of the MmpS6-MmpL6 operon in Mtb physiology [251]. The MmpL proteins are integrated into the mycobacterial plasma membrane and their primary functions consist of transporting ciderophores and virulence-associated lipids to the periplasmic space where they play a key role in the cellular envelope structure and dynamics [415]. MmpS proteins are considered to act as MmpL partners and a growing body of experimental evidence is emerging that suggests that the described system might be involved in the formation of membrane-associated scaffolds that orchestrate not only lipid transport, but also biosynthesis, and regulate drug resistance and heme uptake [416]. In this light, MmpL-MmpS proteins are studied as possible candidates for novel treatment strategies. Activation of the MmpS6-MmpL6 operon in the presence of various redox active compounds (triclosan; plumbagin, PLB; and juglone), but not in the case of menadione has been demonstrated [251]. It was suggested that the latter did not enter bacterial cells with sufficient efficiency or was not capable of inducing sufficient oxidative stress due to the lack of the extra hydroxyl group that facilitates semiquione formation and is part of the PLB and juglone structures. The introduction of the MmpS6-MmpL6 operon to lineage (L) 3 cells rescued their growth under the conditions of triclosan and PLB treatment, this feature was also observed to some degree for L1 cells with these genes already present in their genome [251]. This result was in line with imaging using the Mrx1-roGFP2 sensor that revealed a decreased reduced/oxidized mycothiol (MSH/MSSM) ratio in L3 cells in contrast to L1 and operon-complemented L3 cells in the presence of PLB [251]. Therefore, the data highlights the role of the MmpS6-MmpL6 operon in the ability of Mtb to confer redox stress. 

A paper by Linzner et al. dedicated to the investigation of the physiological role of YpdA in *S. aureus* is another example of how studies using GEFIs clarify the functions of largely unexplored proteins [260]. *S. aureus*, like many other firmicutes, utilizes bacillithiol as a primary low molecular weight thiol for buffering the redox state of the cysteine residues within the proteome, detoxifying antibiotics and toxic electrophiles, and for metal homeostasis [417]. While it is established that BrxA and BrxB are responsible for the protein de-becillithiolation step [418], the reductase of bacillithiol remains unknown. Indirect genetic [419] and biochemical [420] evidence exist that YpdA plays this role and it has been experimentally shown that its overproduction increases the level of reduced bacillithiol (BSH) and contributes to oxidative stress resistance [420]. With the use of the Brx-roGFP2 and Tpx-roGFP2 probes, Linzner et al. investigated the BrxA/BSH/YpdA electron pathway in various physiological conditions, and these experiments along with in vitro enzyme essays supported the described hypothesis about YpdA functions in vivo [260]. 

Redox biosensors do not only allow well known proteins to be characterized in more detail, but also the discovery of novel antioxidant systems. To complement the map of redox pathways interactions in Mtb, Nambi et al. constructed saturating transposon libraries of WT and CtpC deficient Mtb and subjected them to selection in mouse spleen tissue [245]. CtpC is required for Mn^2+^ integration into SodA [421], the enzyme crucial for detoxifying superoxide that is considered the primary oxidant during phagocyte oxidative burst. The protein product of the gene named *DoxX*, one of the strong alleviating interactors of *CtpC*, was shown to act as a scaffold of a thiosulfate oxidizing complex that included SodA and the product of another *CtpC* alleviating interactor, *SseA* [245]. Enzymatic activity in vitro was dependent on the presence of both DoxX and SseA, but not on SodA. Cells lacking DoxX or SseA demonstrated increased sensitivity to tert-butyl-hydroperoxide and cumene hydroperoxide (CuOOH), known thiol oxidizing agents [245]. The role of this complex in thiol recycling was additionally supported by the decline in diamide reduction rate in conditions where one of these enzymes was absent. Imaging with the use of Mrx1-roGFP revealed a decreased MSH/MSSM ratio in the mutant strains suggesting that ROS produced during respiration are not eliminated with sufficient efficiency [245]. Moreover, cells lacking DoxX or SseA were characterized by elevated markers of lipid peroxidation which confirms that they face oxidative stress [245]. The proposed model is that DoxX/SseA complex catalyzes the electron flux from the thiyl radicals to molecular oxygen and, therefore, association with SodA is needed to eliminate the resulting compounds at the point of their generation [245]. The authors note that their study demonstrates the significance of determining genetic interaction networks in vivo as these conditions allow physiologically relevant metabolic processes to be identified. A paper by Fritsch et al. is one more example of a study in which a redox biosensor (Brx-roGFP2) was implemented to investigate the physiological functions of the poorly characterized MhqR regulon of *S. aureus* [262]. This work revealed its role in quinone and antimicrobial resistance. 

Another set of studies addresses the question of redox homeostasis in various genetic contexts and growth conditions. *E. coli* SHuffle cells represent a bacterial system with oxidizing cytosolic conditions for the expression of proteins containing disulfide bonds which is achieved by the removal of thioredoxin reductase and glutathione reductase [422]. Despite being characterized by a remarkably low cytoplasm *E_GSH_* (about −250 mV) [240], SHuffle cells are still far from modeling the conditions of the ER where *E_GSH_* is about −180 mV [423]. Investigation of their redox metabolism is therefore important for the future engineering of more powerful expression strains. Reuter et al. demonstrated that in contrast to the *soxRS* regulon that is activated in the presence of superoxide, in SHuffle cells *oxyR* regulon was upregulated, indicating that these bacteria experience endogenous H_2_O_2_ stress [240]. Western blots with 4-acetamido-4′-maleimidylstilbene-2,2′-disulfonic acid implementation revealed that about 90% of Grx1-roGFP2 molecules were reduced in WT cells and this portion fell to only 10% in the SHuffle strain. For the roGFP2-Orp1 probe the corresponding numbers were about 100% and 80%, respectively [240]. It was shown with flow cytometry analysis of excitation ratios that oxidation states of both sensors were close to their limits in SHuffle cells and on average 2–3 fold more oxidized than in the case of the WT strain. It is interesting to note that while the recorded distributions of Grx1-roGFP2 expressing cells were similar for the SHuffle strain and WT strain treated with oxidants, indicating that the cells experienced similar GSSG-driven stress in these conditions, the distribution of roGFP2-Orp1 expressing SHuffle cells was shifted to the larger values compared to WT cells treated with diamide or H_2_O_2_ [240]. This suggests that SHuffle cells experience even more pronounced H_2_O_2_-driven stress than WT cells in the presence of oxidants [240]. In cultivation experiments, both SHuffle and WT cells accumulated GSSG slowly during aging and plateaued at the stationary phase, however, in the case of the former the overall change was smaller due to the more oxidized initial state. Another behavior was registered with the use of roGFP2-Orp1 probe; while WT cells gradually increased the oxidation degree, SHuffle cells were already in the fully oxidized state in the growth phase [240]. 

The elevated oxidative burden in stationary phase bacteria was found in other studies. In particular, it was observed in the *S. aureus* USA300 and COL strains with Brx-roGFP2 [89] as well as in *Salmonella* Typhimurium cells that lack catalases and peroxidases with roGFP2 [235]. Being deficient in antioxidant systems, this strain allows endogenous ROS to be recorded during respiration and other metabolic processes without the interference of rapid detoxification. With the same strain it was demonstrated that nutrient rich mediums and lower temperatures cause similar effects [235]. However, these trends are not universal. Thus, in a study by Tung et al., mycothiol redox potential (*E_MSH_*) in *C. glutamicum* cells, an industrially important bacterium [424] and a model organism for several related pathogens [425], was characterized by a value of approximately −300 mV during both the log and stationary phases as revealed by Mrx1-roGFP2 [74]. The depletion of MSH metabolism by knockout of either *mshC*, a gene required for MSH synthesis, or *mtr*, a gene required for NADPH-dependent MSSM reduction, resulted in a statistically significant increase of the *E_MSH_* value [74]. A similar effect was achieved by the removal of SigmaH factor (*sigH* gene) that orchestrates the expression of the disulfide stress regulon, including the genes for thioredoxins, mycoredoxin-1 and several genes involved in MSH metabolism [74]. By contrast, the elimination of OxyR, a transcriptional repressor crucial for adjusting H_2_O_2_ resistance, did not result in any statistically significant alterations [74]. Cells lacking its target genes, namely, *katA*, *tpx*, and *mpx*, also did not differ from the WT strain, indicating that in the tested conditions their protein products do not play a key role in sustaining *E_MSH_* [74]. In another study using Mrx1-roGFP2, it was shown that the *E_MSH_* values of WT, MSH-negative (Δ*mshA*), MSH-depleted (Δ*mshD*), and SigH-deleted *Mycobacterium smegmatis* (Msm) cells were approximately −300 mV, −239 mV, −275 mV, and −300 mV [52]. Therefore, in contrast to *C. glitamicum* the loss of SigmaH factor in Msm does not affect the MSH redox state significantly. As Δ*mshD* cells contain only 1%–3% of normal MSH [426], the lower *E_MSH_* value compared to the Δ*mshA* context may be due to the moderate ability of Mrx1 to equilibrate the redox-active cysteines of roGFP2 with Suc-mycothiol and formyl-mycothiol that are present in these cells [52]. Similarly, the disruption of BSH synthesis in the *bshA* and *bshC* mutants of *S. aureus* led to the complete oxidation of the Brx-roGFP2 probe [89]. No differences were registered between MSH redox potentials in drug-resistant (Jal 2261, 1934, Jal 2287, MYC 431) and drug-sensitive (Mtb H37Rv, *M. bovis* BCG) Mtb strains under laboratory growth conditions. However, being located in the −273–280 mV range, all values were notably higher compared to Msm [52]. With the use of the Peredox-mCherry sensor, Bhat et al. demonstrated that deletion of *sigH*, *mshA*, or *mshD* increased the NADH/NAD^+^ ratio in Msm cells confirming a functional link between this redox couple and thiol recycling pathways [250]. In the same system, the authors also demonstrated that inhibition of the ETC at different points resulted in a ratio increase suggesting that in Msm the ETC is a major contributor to the utilization of electrons from NADH [250]. The disruption of either membrane potential or proton moving force also led to a ratio increase, while the disruption of both at the same time (proton motive force) acted in the opposite manner [250]. 

As briefly mentioned above, the interplay between redox homeostasis and temperature is a separate field of interest. *Lactococcus lactis* is a mesophilic bacterium that is widely used in the production of dairy products, in particular, various types of cheese [427]. Understanding of its metabolism is of great importance as it paves the way for the optimization of the technological processes. Chen et al. demonstrated that the elevated temperature (37 °C vs. 30 °C), to which *L. lactis* is exposed during food manufacturing, leads to a general decline in the growth rate that could be salvaged by oxygen elimination or increased riboflavin supply [242]. In these conditions the intracellular FAD content was significantly reduced correlating with the decrease in the activities of the FAD-dependent enzymes, NADH oxidase and pyruvate dehydrogenase [242]. Taking into account that NADH oxidase is crucial for lowering molecular oxygen concentration and that thioredoxin reductase also requires FAD for its functioning, it could be expected that *L. lactis* would suffer from oxidative burden at high temperatures; this was confirmed by imaging with a roGFP probe [242]. The external addition of acetate, the building material for cellular membranes, which cannot be produced under conditions of disrupted pyruvate dehydrogenase activity, and the overexpression of the riboflavin transporter RibU improved the growth rate of *L. lactis* under heat shock [242]. 

In addition to temperature, the acidity of the growth medium constitutes another important cultivation factor. It is known that in rich medium the growth of Mtb is slowed down at pH 6.4 and arrested at pH 5.0 [428]. However, experimental evidence exists that cytoplasmic acidification is not a major contributor to such behavior [429]. Baker et al. established that the carbon sources that feed central metabolism at the anaplerotic node, namely phosphoenolpyruvate, pyruvate, oxaloacetate, and acetate rescued the growth of various Mtb strains (CDC1551, H37Rv, HN878, and Erdman) at pH 5.7 [254]. Interestingly, Msm is not prone to such inhibition, which suggests that the described behavior is somehow related to the Mtb pathogeny. In acidic conditions, glucose but not pyruvate, being a single carbon source, induced cytosolic reductive stress as visualized by roGFP2, which was enhanced under disrupted functioning of the *phoPR* regulatory system [254] that participates in adaptation to low pH. Further transcriptome studies revealed activation of the *mmpL8-pks2* operon [254] which is known to control sulfolipid synthesis in a *phoPR*-dependent manner [430,431]. Indeed, the concentration of these compounds was 3-fold induced at pH 5.7 compared to pH 7.0. The authors suggest that the *phoPR* system lowers the growth rate in acidic conditions by directing carbon towards lipid synthesis, a process that requires reductive equivalents, for the purpose of confronting redox stress [254]. 

Redox biosensors allow recording of the real-time dynamics of oxidative stress that is important for understanding the roles of different enzymes in ROS detoxification and the temporal parameters of these reactions. First of all, studies with the implementation of GEFIs shed light on their functioning in live systems. Uncoupled roGFP family probes rely on host glutaredoxins for their sensitivity and in animal cells their equilibration with the cellular GSH/GSSG pool proceeds slowly [88]. However, experimental evidence exists demonstrating that in bacterial cells these instruments develop a response to the oxidant addition quite quickly [235,237,259], which reveals differences in the temporal properties of the corresponding bacterial systems compared to the animal ones. Similar observations exist in regard to yeast systems [12]. Conversely, a direct comparison of the Mrx1-roGFP2 and roGFP2 dynamics in Msm after H_2_O_2_ addition revealed that in the latter case the maximum amplitude of response and its onset were both diminished as well as anti-oxidative recovery [52]. Therefore, a possible difference in the behavior of coupled and unfused redox-sensitive FPs should not be neglected even during imaging in microorganisms. 

Using Michaelis-Menten correlation, van der Heijden et al. measured H_2_O_2_ detoxification rates and the total antioxidant capacities of various Gram-negative bacteria using roGFP [235]. The data indicated that even closely related strains can demonstrate pronounced differences. This study provides a detailed investigation of how growth conditions (medium, pH value and temperature) influences the discussed parameters in *S. Typhimurium* and reveals that the antioxidant capacity does not always correlate with survival after oxidative stress [235]. The authors showed that the total antioxidant capacity increased at the stationary phase and this phenomenon was dependent on catalases and/or peroxidases, as the strain lacking these enzymes did not demonstrate such a phenotype [235]. Pre-treatment of bacteria with 500 μM H_2_O_2_ resulted in an increase of both detoxification rate and capacity; this effect was dependent on protein synthesis, as incubation of the cells in the presence of chloramphenicol prevented its emergence. Further investigation established the role of OxyR in this adaptation [235]. The priming responses to sublethal H_2_O_2_ doses were also studied in *E. coli* cells using Grx1-roGFP2 and roGFP2-Orp1 [240]. In *S. Typhimurium*, the elimination of all catalases significantly reduced both the detoxification rate and antioxidant capacity while elimination of all peroxidases increased these parameters, suggesting that over-compensation occurs in these conditions. Detailed examination of the individual catalases revealed different roles for these enzymes: KatE and KatG make a greater contribution to the total capacity while KatG is responsible for the detoxification rate [235]. The role of OxyR in H_2_O_2_-induced stress was also proven in *C. glutamicum* where the Mrx1-roGFP2 signal after oxidant addition reached smaller values in the knockout strain than in WT cells, which was additionally confirmed by investigation of bacteria deficient in *katA*, its target gene [74]. In the latter case, the maximum signal was more than twofold larger than in normal cells and did not return to the initial value during the observation period. By contrast, *sigH* mutants demonstrated non-disturbed dynamics, suggesting that MSH does not play a direct role in the response to H_2_O_2_ [74]. Cells lacking *mpx*, *tpx*, or both did not significantly differ from the controls although it was previously hypothesized that *mpx* compensates for *tpx* inactivation [74]. These studies support the concept that catalases play the primary detoxification role under conditions of high H_2_O_2_ concentrations, because low levels of NAD(P)H during oxidative stress might be not sufficient for peroxidases recycling. 

As H_2_O_2_-induced responses cannot be observed in SHuffle cells due to the initially high degree of roGFP-based probes oxidation, the temporal parameters of recovery after dithiothreitol-induced reduction were recorded using the Grx1-roGFP2 and roGFP2-Orp1 sensors confirming the different powers of basal GSSG- and H_2_O_2_-driven oxidative stresses mentioned above [240]. The deletion of MSSM reductase resulted in a two-fold increase of the response amplitude and slowed-down the recovery rate in Mrx1-roGFP2 expressing Msm cells under H_2_O_2_ exposure, supporting the role of this enzyme in anti-oxidative defense [52]. The signal recovery was dramatically delayed in Mtb (~120 min) compared to Msm (~5 min), which highlights the differences in the antioxidant systems of these species [52]. In a study by Bhat et al., the effects of a wide set of oxidants on the NADH/NAD^+^ ratio were investigated in Mtb using Peredox-mCherry at two time points (6 h and 24 h of incubation) [250]. Interestingly, the NO donor spermine NONOate did not induce any significant changes, which is surprising in the context of its ability to inhibit the ETC [250]. 

Several reports have been published that address the question of HOCl-induced stress dynamics which is extremely important in the context of pathogenic bacteria as these organisms might face (pseudo)hypohalous acids during interaction with the immune system of the host [432]. Thus, addition of NaOCl to *C. glutamicum* at different concentrations led to responses that were not reversible during the time of imaging in contrast to H_2_O_2_ treatment [74]. Similarly, NaOCl-treated *S. aureus* cells expressing Brx-roGFP2 required more time for antioxidant-mediated recovery than H_2_O_2_-treated cells [89]. Signal recovery was affected in the *S. aureus* mutant lacking YpdA, a promising candidate for BSSB reductase [260]. H_2_O_2_ induced a similar behavior of the sensor in this genetic context, supporting the role of YpdA in ROS-resistance. However, cells that were deficient in *brxA* and *brxB* did not significantly differ from the control [260]. It should be noted, that any experiments in which roGFP-based probes are expressed in conditions where HOCl production is expected must be interpreted with the utmost caution because these sensors can be directly oxidized by both HOCl and its derivatives such as N-chlorotaurine [89,94,97]. More information on this issue can be found in Section 5.6.

Finally, redox biosensors are capable of answering the question of whether a compound affects the redox homeostasis of cell. Despite the experimental evidence obtained from several studies that oxidative stress is a general mechanism of action of aminoglycosides, quinolones and β-lactam antibiotics [433,434,435], roGFP expressed in *S. Typhimurium* actually demonstrated a decrease in its oxidation degree in the presence of gentamicin, kanamycin, streptomycin, ciprofloxacin, and cefotaxime which might be due to slowdown of respiration [235]. In another study, Loi et al. observed no Brx-roGFP2 signal changes in *S. aureus* under exposure to the sub-lethal doses of erythromycin, rifampicin, vancomycin, ciprofloxacin, gentamicin, ampicillin, fosfomycin, lincomycin, linezolid, and oxacillin [89]. Zinc pyrithione and triphenyl tin are organo-metallic biocides whose molecular mechanisms of toxicity remain poorly studied. Their application to roGFP2 expressing *E. coli* cells revealed a concentration-dependent decrease in GSH/GSSG ratio as reported by the sensor, suggesting the presence of a redox-related component in their action [237]. Several studies have addressed the question of ROS production caused by cations of heavy metals and metalloid oxides which are known environmental pollutants [235,237,238]. However, the results are relatively controversial as some of these compounds can directly interact with redox active cysteines of roGFP family probes. In another study, it was shown that the antibacterial coating AGXX^®^ (Largetec GmbH, Berlin) promotes BSH oxidation in *S. aureus* expressing Brx-roGFP2 [261]. This material includes transition metals (silver and ruthenium) conditioned with ascorbic acid and, therefore, acts via production of toxic ROS [436]. 

It was suggested that live bacteria expressing roGFP2 can be implemented as sensor systems for ecological screening because they take into account the bioavailability of toxic substances and their responses are much faster than those of transcription-based assays [237]. The immobilization of cells into a κ-carrageenan matrix resulted in a significant improvement of storage time from 10 h to 46 days at 4 °C with a fluorescence intensity decrease of only ~20% [238]. This system is compatible with plate readers and provides a faster response, larger linear region of the signal and lower limit of detection than the alternative approaches [238]. 

#### 5.1.2. Oxidative Stress in S. cerevisiae

**Figure 5:**
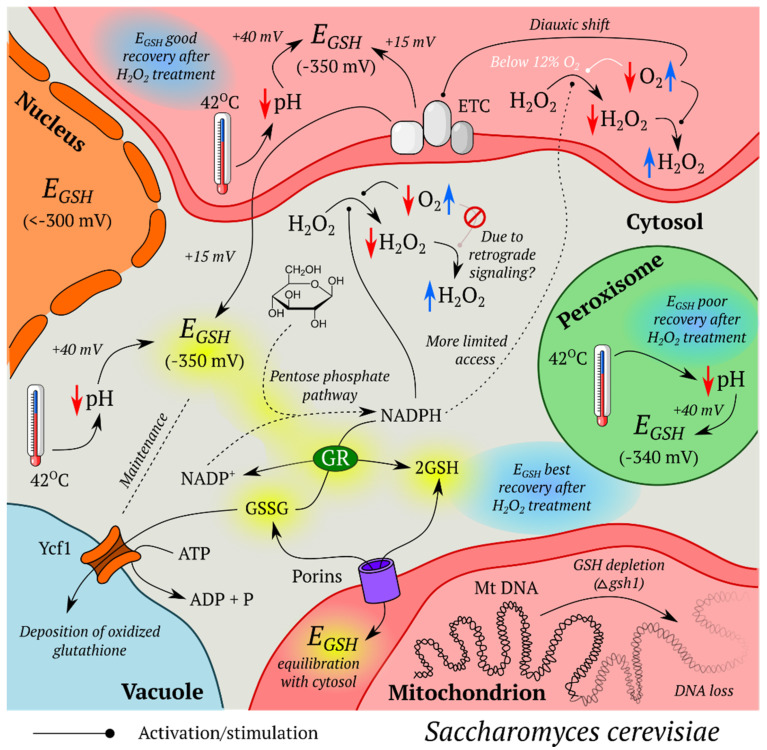
Compartment-specific features of selected redox processes in yeast cells. The picture summarizes the mechanisms of glutathione redox potential (*E_GSH_*) maintenance and its dependence on the temperature, as well as the dynamics of H_2_O_2_ concentration shifts in response to changes in oxygenation degree in cytosol, mitochondria and peroxisomes [12,14,32,46,205,212]. Abbreviations: ETC—electron transport chain; GR—glutathione reductase; GSH—reduced glutathione; GSSG—oxidized glutathione; Mt DNA—mitochondrial DNA.

Generally, studies with redox biosensors in yeast address similar questions, as in case of prokaryotic microorganisms. Traditional analytical protocols often fail to reveal redox shifts under the tested conditions and, most likely, the fact that they rely on whole cell measurements is responsible for their low accuracy because it may not reflect redistribution of reduced/oxidized species between different cellular compartments, nor pH alterations which affect the thermodynamics of redox couples. Using roGFP2, Ayer et al. estimated the *E_GSH_* values of *S. cerevisiae* cytosol and mitochondria to be in the range of −340–350 mV when grown in fermentative medium [12]. At the stationary phase the corresponding values increased by approximately 10–15 mV which might be attributed to ETC activation caused by the switch to aerobic respiration during the diauxic shift (Figure 5) [12]. For cells that were initially grown in the respiratory medium, no changes were observed, and the initial redox potentials were already shifted towards more positive numbers, indicating that the respiration status plays a bigger role in adjusting GSH homeostasis than the growth phase. *E_GSH_* in peroxisomes was approximately −340 mV under all tested conditions [12]. It is important to note that the potential values reported in this work are more negative than in earlier studies [31,437], mostly because the authors utilized the pHluorin probe for direct acidity measurement which revealed that the pH-statuses of the imaged organelles were more basic by 0.2–0.3 units than the previous estimates [438]. The issue of endogenous H_2_O_2_ generation at different oxygenation levels was investigated with the use of mito- and cyto-targeted roGFP2-Tsa2ΔC_R_ probes [14]. A gradual decrease in oxygen availability from 18% to 1% over a time period of 5 h was followed by a corresponding reduction of the cytosolic reporter while the mitochondrial reporter showed a noticeable delay until the oxygen concentration reached a value of 12% (Figure 5) [14]. It seems that mitochondria possess a more powerful redox buffer for softening oxygen fluctuations. Alternatively, the observed dynamics might be explained in terms of subcellular oxygen redistribution in favor of the ETC during the initial stage of hypoxia. Interestingly, in contrast to the mitochondrial sensor that was rapidly oxidized following reoxygenation, the cytosolic redox status remained unchanged during the whole observation period [14]. One explanation for this is the readjustment of redox homeostasis in this compartment due to the activation of retrograde signaling which was detected in this study. 

Experimental evidence exists that in yeast many types of environmental stress are accompanied by oxidative components, and upregulation of antioxidant systems can be detected in these conditions [439,440,441]. In *S. cerevisiae*, heat-shock induced by heating cells from 25 °C to 42 °C for 60 min led to an *E_GSH_* increase of 40 mV in the cytosol, mitochondria, and peroxisomes (Figure 5) [12]. However, as revealed by pHluorin the observed shift was mostly driven by acidification, as the roGFP2 excitation ratio was not significantly affected during the experiment. In the same study, hyperosmotic stress caused by 1.8 M sorbitol, 0.9 M NaCl, or 1.3 M KCl treatments for 60 min did not alter pH or *E_GSH_* values [12].

Redox homeostasis is a dynamic interplay between emerging oxidative and reductive events in cells. In *S. cerevisiae* grown in synthetic defined medium, supply of glucose resulted in more rapid and strong reduction of the roGFP2-Tsa2ΔC_R_ probe after depletion of dissolved oxygen [14]. The contribution of reducing equivalents to the observed phenomenon was established by imaging non-respiring Δ*cox7* mutants in which the initially oxidized sensor showed noticeable reduction after glycose addition. Access to the reducing equivalents was significantly higher for cytosol because the signal of the corresponding probe reached the value of the control cells while in mitochondria only a modest shift was observed (Figure 5) [14]. The oxidative pentose phosphate pathway is one of the major sources of NADPH that influence the redox status of peroxiredoxins, as confirmed by an increased oxidation degree of the sensor either in the presence of glucose-6-phosphate dehydrogenase (G6PD) inhibitor CB83 or in the cells deficient in this enzyme [14]. 

As in bacteria, redox sensors allow the dynamics of exogenous oxidative stress in yeast to be recorded. Treatment with 1 mM H_2_O_2_ resulted in *E_GSH_* value increases of 40–50 mV and pH value declines of approximately 1 unit in the mitochondria, cytosol, and peroxisomes [12]. High doses of H_2_O_2_ shifted the redox potentials by ~100 mV in all cases. Different recovery kinetics after incubation with H_2_O_2_ (2 mM, 30 min) was observed for three compartments: while the cytosol and mitochondria re-gained 80–90% of the observed shift mostly within 4 and 10 min, respectively; peroxisomes recovered by only 25% and plateaued after 10 min of imaging (Figure 5) [12]. This might be attributed to the fact that the number of antioxidant enzymes in *S. cerevisiae* peroxisomes is relatively modest. A low adaptive dose of H_2_O_2_ (0.2 mM, 1 h) did not led to a pronounced change in *E_GSH_* itself; however, it lowered the observed shift after treatment with 2 mM H_2_O_2_ for 30 min by 10–15 mV, suggesting that the change in GSH redox potential is not the mechanism of preadaptation to this oxidant [12]. Moreover, the knockout of Yap1 and Skn7p, transcription factors that are key mediators of H_2_O_2_ resistance [442], elevated the mitochondrial and cytosolic *E_GSH_* values by ~20 and ~30 mV, respectively; but in the preadaptation test the final values reached after 2 mM H_2_O_2_ treatment were similar to those for the WT cells. Given that, the authors conclude that it is unlikely that redox state changes per se are important subsequent events in the cellular adaptation to H_2_O_2_ [12]. The impact of reducing equivalents coming from glucose on the roGFP2-Tsa2ΔCR signal dynamics after H_2_O_2_ treatment was also investigated in WT and G6PD-deficient yeast [14]. In normal cells, the presence of glucose reduced the maximum oxidation degree of the probe and accelerated recovery. This paper also reports interesting data about NAD(P)H levels in the studied system measured by autofluorescence [14] which is beyond the scope of the current review. 

At present the exact molecular mechanisms by which even well studied oxidants such as H_2_O_2_ affect cellular metabolism are not fully understood. In contrast to much more reactive compounds like (pseudo)hypohalous acids [443,444], H_2_O_2_ is characterized by relatively low reaction rates with biological molecules [445] and in that light direct damage seems not to be the pathway underlying the effects of pronounced H_2_O_2_-induced stress. An outstanding paper by Calabrese et al. in which this issue was investigated with the combination of Grx1-roGFP2 and roGFP2-Tsa2ΔCR reporters significantly complements the modern knowledge about H_2_O_2_-mediated toxicity [15]. In their work, the authors demonstrated that the antioxidant activities of cytosolic Tsa1 and Tsa2 protect the mitochondrial matrix from exogenous H_2_O_2_ and at the same time Por1 participates in excretion of endogenous ROS generated in the ETC from the IMS to the cytosol. It was shown that in conditions of Prx1 and Trx3 deficiency, when mitochondrial antioxidant defense is weakened, the cytosolic catalase Ctt1 becomes upregulated providing additional cytoprotection [15]. Despite the existence of the described detoxification mechanisms, the response of matrix Grx1-roGFP2 to exogenous H_2_O_2_ treatment was quite pronounced. Experiments on the modulation of Glr1 activity revealed that this phenomenon was only partially attributed to the insufficient ability of the enzyme to reduce mitochondrial GSSG compared to the cytosolic pool [15]. Interestingly, cells lacking mitochondrial Prx1 did not demonstrate significant shifts in the spectrum of the matrix Grx1-roGFP2 under treatment with high H_2_O_2_ concentrations, suggesting that oxidation of mitochondrial GSH in response to this oxidant is Prx1-dependent. Preadaptation of cells with either a high exogenous H_2_O_2_ concentration or endogenous ROS production in the ETC decreased the repose of the matrix Grx1-roGFP2 to 1 mM H_2_O_2_ addition which arose from the fact that over-oxidized cysteine in Prx1 was not capable of equilibrating with the GSH/GSSG couple. In line with that, Prx1 was shown to protect yeast growing in the continuous presence of H_2_O_2_; however, its deletion was advantageous under acute H_2_O_2_ stress (>10 mM) [15]. When natural Prx1 was substituted for a truncated version more resistant to over-oxidation, cells became more sensitive to acute H_2_O_2_ stress-induced cell death (10 mM) than the WT control. Finally, the authors addressed the question of whether cell death in these conditions was attributed to GSH oxidation itself or if the latter was just a step towards NADPH depletion. Yeast Glr1 mutants that cannot reduce GSSG in the matrix and, therefore, preserve NADPH were severely impaired in their ability to survive acute H_2_O_2_ stress which was partially improved by Prx1 deletion. In contrast, substitution of natural Prx1 for the over-oxidation resistant version worsened the phenotype confirming that matrix GSH oxidation promotes cell death [15]. Given that many eukaryotic typical 2-Cys peroxiredoxins contain GGLG and YF motifs that are required for susceptibility to over-oxidation [446,447], the authors hypothesize that the described mechanism is physiologically relevant [15]. 

In some studies, redox sensors are utilized to clarify the functioning mechanisms of toxic compounds. 4,4′-dipyridyl disulfide (DPS) is a membrane-permeable thiol oxidant [448] capable of reacting with GSH, as shown in both in vitro and in vivo studies [449,450]. A study by Lopez-Mirabal et al. was the first to investigate its effects on redox homeostasis of *S. cerevisiae* [200]. By testing mutants for the most important antioxidant pathways the authors discovered a set of genes that showed marked abilities to influence DPS sensitivity. The most pronounced effects were observed for *trx2*, *sod1*, *glr1*, and the *grx1/grx2* double combination. With the use of rxYFP it was shown that the sensitivity of various mutants to DPS correlated with the degree of the probe’s oxidation [200]. Moreover, in contrast to WT cells, DPS induced significant shifts in the GSH/GSSH ratio of Δ*glr1* mutants indicating, that on the one hand GSH depletion was not the major cause of cell damage in that system, but on the other that the DPS targets depended on this low molecular thiol. This study was also one of the first to indicate that thioredoxins apparently contribute to the adjustment of the cytosolic GSH redox potential [200]. In subsequent work, the DPS-induced effects on redox homeostasis in ER and their interplay with the Ero1 pathway were investigated in detail [201]. In another study roGFP was implemented to register redox changes in *S. cerevisiae* treated with NaAsO_2_ and Pb(NO_3_)_2_ [203]. The dose-dependent behavior of the signal was observed which was mitigated in the presence of the hydroxyl radical scavenger thiourea. Allicin, an antimicrobial agent from garlic, also induced roGFP2 oxidation [204]. 

With GEFIs, it becomes possible to not only establish redox-dependent patterns of toxic compounds action, but also identify the roles of various proteins in redox homeostasis. Cdc48 (p97/VCP in mammals) is an important regulator of proteasomal degradation pathways such as ER-associated degradation [451] and mitochondria-associated degradation [452]. Besides its role in misfolded protein targeting for degredation, data exists showing that it possesses direct refolding activity [453]. Previous research revealed that Cys115 in the Cdc48 N-terminal domain undergoes oxidation during chronological aging which precedes massive thiol modifications emerging after the metabolic diauxic shift [454]. A paper by Radzinkski et al. provides experimental evidence that Cys115 serves as a redox sensor crucial for sustaining Cdc48 cytosolic localization under conditions of oxidative stress, including that observed during senescence [48]. In this work, Grx1-roGFP2 allowed cellular populations with different *E_GSH_* to be isolated for investigation of the effect of the Cys115Ser substitution on their growth. Moreover, the mass spectrometry analysis of Cdc48 interactom suggested that a set of antioxidant proteins are either misfolded substrates of Cdc48 or that they regulate the redox homeostasis of Cdc48-associated proteins. The authors drew attention to the fact that since mutations in VCP are connected to several human diseases [455], redox metabolism of affected cells should be carefully investigated in future research [48]. 

Some studies with the use of redox sensors address specific but no less interesting questions. Fe/S cluster-containing proteins are extremely vulnerable to oxidation in their apo-forms because their cofactor-coordinating cysteines become more accessible to the external medium [13,456]. In eukaryotes, early Fe/S cluster assembly proceeds in mitochondria [457], which puts them in a tough situation as these organelles are one of the major sources of ROS production in cells. Christ et al. established that under conditions, when the first steps of Fe/S cluster assembly are disrupted, the mitochondrial [4Fe-4S] apo-proteins are purified with their coordinating cysteines being blocked [13]. Subsequent experiments combining the use of thiol-binding agents and mass-spectrometry led to a model in which these residues undergo physiologically relevant cyclic S-polythiol modifications which serve as a reversible protective mechanism. Imaging with the roGFP2-Orp1 sensor targeted to either cytosol or mitochondria revealed that the latter provide more oxidizing conditions capable of sustaining the discussed modifications [13]. 

Nowadays it is widely accepted that oxidative stress not only leads to cellular damage and corresponding pathology development, but also allows live organisms to adapt to the changing environment. Despite not having specialized photo-sensing machinery represented by opsins, phytochromes, and cryptochromes [458], *S. cerevisiae* demonstrate light-dependent behavior, in particular, rhythmical changes in subcellular localization of the Zn-finger transcription factor Msn2 [459]. Using the HyPerRed sensor, Bodvard et al. established that light exposure induced H_2_O_2_ production in yeast which could be mitigated by elimination of Pox1, the yeast homologue of peroxisomal acyl-CoA oxidase [216]. This also affected nuclear accumulation of Msn2 under illumination. This phenomenon apparently arose from the fact that light promotes photoreduction of the flavin cofactor inside this protein with subsequent H_2_O_2_ generation, a feature that explains acyl-CoA oxidase-mediated blue light phototoxicity in mammalian cells [460]. In line with this, exogenous H_2_O_2_ addition as well as modulation of the mitochondrial cytochrome c peroxidase Ccp1 was shown to adjust Msn2 redistribution in this system [216]. In cells lacking Tsa1, Msn2 photo-behavior was lost, which depended on thiol redox cycling in this protein, confirmed by experiments with either mutations of the key cysteine residue, or removal of the carboxy-terminal YF motif crucial for over-oxidation susceptibility. A mutant, deficient in Trx1 and Trx2 also demonstrated predominantly cytosolic Msn2 localization under illumination, suggesting that Tsa1 acts as a sensor for Pox1-generated H_2_O_2_ that further transmits oxidative equivalents to these proteins, controlling targeting of Msn2 [216]. Interestingly, in a relatively large number of works, peroxiredoxins were shown to participate in modulation of molecular clocks and other forms of rhythmic behavior [461,462,463,464]. Further experiments complemented the described picture with clarification of PKA role in this system [216]. It seems that the electron route discovered relieves Msn2 from inhibitory serine phosphorylation in the nuclear export sequence and it requires PKA nuclear-cytosol transition as a step. Given the high degree of conservation of all the components of the pathway, the authors concluded that H_2_O_2_ might be a universal secondary messenger coupling light and circadian clocks with peroxiredoxins as general sensors [216]. 

### 5.2. Glutathione and Thioredoxin Systems

GEFIs have also contributed to the study of the glutathione and thioredoxin systems. The redox status of the cytosolic glutathione pool of *S. cerevisiae* was assessed in [199]. The nuclear glutathione redox state was found to be highly reducing, similar to that of the cytosol under steady-state conditions (Figure 5) [46]. Morgan et al. investigated changes in the redox state of the cytosolic pool of glutathione in response to severe oxidative stress and found that it was robustly maintained [212]. The authors showed that the ABC-C transporter Ycf1 mediated the transport of GSSG, which was not immediately reduced in cytosol, to the vacuole. Therefore, extracytosolic GSSG accounted for changes in the whole-cell glutathione redox state, while cytosolic glutathione homeostasis was accurately regulated (Figure 5) [212]. The IMS was observed to be considerably more oxidizing than the mitochondrial matrix and cytosol, which might support oxidative folding of imported proteins [31]. The authors also provide evidence for the existence of independent glutathione pools in the cytosol, matrix and IMS, which might be caused by a different thiol-disulfide balance in the compartments [31]. However, Kojer et al. demonstrated that steady-state *E_GSH_* in the IMS was similar to that of the cytosol [32]. It was suggested in the work that the glutathione pools in the IMS and the cytosol were interconnected via porins, and that the IMS relied on the cytosolic glutathione redox system for the regulation of its *E_GSH_* (Figure 5). At the same time, matrix *E_GSH_* was shown to be regulated independently of the cytosol and the IMS [32]. In [33] Kojer et al. addressed the question of how oxidation-driven protein import to the IMS was possible if *E_GSH_* of the cytosol and IMS was similarly reducing. It was demonstrated that the IMS harbored the glutaredoxin activity, which mediated the influence of the IMS glutathione pool on IMS proteins, but the limiting amounts of glutaredoxins provided a kinetic barrier to prevent the thermodynamically feasible reduction of proteins. Thus, in the reducing environment of the IMS, efficient oxidative folding may be allowed by the accurate regulation of glutaredoxin levels [33].

ER redox homeostasis has also been investigated in several studies using roGFP variants [37,197,208]. Delic et al. showed on a *P. pastoris* model that increased oxidative folding of proteins in the ER had a strong effect on the redox environment in the cytosol, leading to more reducing conditions [37]. Later, Delic et al. continued the research and revealed the specific role of the Yap1 transcriptional factor in oxidative protein folding [197]. Yap1 has been demonstrated to play a role in physiological detoxification of ROS formed upon the process. Overexpression of Yap1 restored cellular redox conditions of protein-secreting yeast by slightly reducing the redox state of the ER and re-oxidizing the cytosol to the WT level [197]. In a study by Puigpinós et al., evidence was provided for *S. cerevisiae* Grx6 to regulate the glutathionylation of thiols of ER/Golgi target proteins and glutathione redox balance in the lumen of these compartments [208]. Also, the influence of Grx6 on intracellular calcium homeostasis was observed. It was proposed by the authors that Grx6 impacts some Ca^2+^ transporter whose activity would be modulated through the deglutathionylation activity of Grx6.

The impact of glutathione on the *S. cerevisiae* mitochondrial genome was studied by Ayer et al. [205]. Glutathione depletion has been found to cause a loss of the mitochondrial genome, and therefore irreversible respiratory incompetency, which occurred both in an iron-independent and iron-dependent manner. Moreover, the redox state of the cytosol, more than that of the mitochondrial matrix or IMS, was shown to change under GSH limitation. Dardalhon et al. also showed that GSH depletion had profound effects on mitochondrial genome stability, while the nuclear genome remained stable (Figure 5) [46]. Braymer et al. investigated the dependence between iron-sulfur protein assembly and the thiol-reducing glutaredoxin and thioredoxin systems [217]. The cytosolic Fe/S protein assembly machinery was observed to be significantly more sensitive to thiol redox enzyme depletion than that of mitochondria, which was found to be functionally robust.

A high throughput study of *S. cerevisiae* redox state using roGFP2 revealed about 100 genes with diverse functions that were required for the maintenance of cytosolic redox potential [19]. An interesting fact found by Ayer et al. was that certain redox-active systems traditionally considered as stress response systems were crucial for steady-state redox homeostasis. The roGFP2 constructs were also targeted to the mitochondrial matrix and peroxisomes, which allowed for the identification of key redox systems on a sub-cellular level [19]. A highly sensitive methodology based on flow cytometry that allowed quantification and isolation of cells with different redox status was suggested by Radzinski et al. [47]. In this study, Grx1-roGFP2 was applied to measure the redox state of individual cells within a heterogenous *S. cerevisiae* population during aging. The cells were sorted based on their oxidation status, and this allowed the phenotypic, proteomic, and transcriptomic profiles associated with the redox environment of cells of similar chronological age to be defined. The proteomic and transcriptomic profiles as well as growth and cellular division of reduced and oxidized cells were observed to differ within the populations. Remarkably, the transcriptome and proteome were found to remain linked to the redox status over 72 h [47].

Several studies have been published in which the thioredoxin system was investigated with GEFIs [45,215]. Oku et al. found that Trx2 was needed for maintenance of the redox homeostasis of *S. cerevisiae* under the starvation and mild-heat stresses of stationary culture [215]. Biddau et al. studied the role of thioredoxins in the biogenesis of the *Toxoplasma gondii* apicoplast, a plastid of apicomplexan parasites, which is essential for pathogen survival throughout their lifecycle [45]. The authors identified two biogenesis pathways controlled by apicoplast Trx1 and Trx2 and suggested that apicoplast thioredoxins might be used as new drug targets.

### 5.3. The UPR in Yeast

The ER is a cellular compartment responsible for oxidative folding and maturation of secretory, transmembrane, and ER-resident proteins. Under certain conditions, ER protein folding capacity is overwhelmed which leads to the accumulation of unfolded proteins. The UPR is a signaling pathway that is activated in this case and is required to restore proper protein folding in the ER [465]. In yeast, Ire1 is a key regulator of the UPR. A well-established signal for Ire1 activation is the accumulation of unfolded proteins in the ER lumen [466]. However, Promlek et al. reported that membrane aberrations caused by the deletion of lipid homeostasis genes, or depletion of inositol, a component of phospholipids, may be an alternative mechanism for Ire1 activation [467]. When Ire1 enzyme is activated, it splices pre-mRNA of Hac1 via cytosolic ribonuclease domain, initiating synthesis of Hac1 transcription factor. Hac1 induces translation of the UPR target genes which act to restore ER protein folding capacity [466]. 

Several studies have been devoted to the study of activation mechanisms of the UPR. It was revealed that an excess of cadmium ions causes the UPR, most probably by the accumulation of unfolded proteins. In contrast, ethanol [41] and diauxic shift [42], a transition from fermentation to respiration in yeast, seemed to induce the UPR independently of unfolded proteins [41,42]. It was also reported that oxidized thioredoxins, that accumulate in mutant yeast lacking thioredoxin reductase, induced the UPR [206]. The exact mechanism by which ethanol, diauxic shift and oxidized thioredoxins activate the UPR have not been identified [41,42,206]. However, for diauxic shift the authors concluded that ROS produced as byproducts of respiration are involved in Ire1 activation [42].

The ER is a more oxidizing compartment than the cytosol which provides optimal conditions for disulfide bond formation during oxidative protein folding [468]. Several research groups monitored changes in *E_GSH_* in the ER after UPR induction. For this purpose, roGFP-based probes targeted to the ER were used. One of these versions, eroGFP, was constructed by Merksamer et al. However, eroGFP was almost fully oxidized in the ER and thus could only detect if the reduction of this compartment occurred after UPR induction [35]. In another work the roGFP1_iE sensor was used. The probe is better adapted for the ER and can measure oxidation of this compartment as well as reduction [37]. Some of the stimuli that activate the UPR led to reduction of the biosensors [35,37,39], however, in other cases, no changes were observed [42]. It should be noted that eroGFP mislocalization was reported in one of the studies: the probe was translocated from the ER to the cytoplasm after UPR induction in a yeast strain with mutations in Ire1 [36]. This phenomenon was recently characterized in the work of Igbaria et al. According to their data, several ER stressing stimuli led to translocation of eroGFP and some other ER-localized proteins to the cytoplasm. The authors called this phenomenon “protein reflux” and supposed that it was one of the mechanisms of ER adaptation to stress [38]. Thus, the observed reduction of the biosensor during the UPR may be caused not only by redox changes of the ER itself but also by translocation of the probe into the cytoplasm which is less oxidized than the ER. Therefore, one should carefully interpret the results obtained with these biosensors if the probe’s localization was not verified during the experiment.

In several studies, the redox state of cytoplasm during ER stress was also monitored with roGFP-based biosensors [37,206]. Delic et al. detected a reduction of the cytoplasm despite ROS production during UPR induction by overexpression of secretory recombinant proteins [37]. However, in the experiment of Kritsiligkou et al. with yeast mutants lacking thioredoxin reductase UPR was accompanied by ROS production and oxidation of the cytoplasmic glutathione pool [206]. The results obtained by these two groups seem to contradict each other, but this can be explained by the different models that were used for UPR activation. According to Delic et al., reduction of the cytosol was a specific reaction to excessive protein folding in the ER and thus in their experiment UPR was accompanied by reduction of the cytoplasmic glutathione pool [37].

### 5.4. Inheritance of Mitochondria in Yeast

In budding yeast, the daughter cell is born with full replicative potential while the mother cell ages and its replicative potential declines with each division. This phenomenon is called mother-daughter age asymmetry and is explained by the fact that damaged cellular components such as protein aggregates, extrachromosomal rDNA circles, defective mitochondria and vacuoles are predominantly retained in the mother cell during division [469]. McFaline-Figueroa et al. discovered that daughter cells inherit on average fitter mitochondria than mother cells. With the use of mito-roGFP1 and the superoxide sensor, it was demonstrated that mitochondria in the bud were slightly more reduced and contained less superoxide than mitochondria in the mother cell [25]. 

The actin cytoskeleton that serves for mitochondria transport plays an important role in mitochondrial segregation between mother and daughter cells [469]. Actin cables are dynamic structures that exhibit retrograde movement in the direction from bud to mother cell, which is known as retrograde actin cable flow (RACF). Two major forces maintain RACF rates and direction: first, the addition of new actin monomers at the bud tip or bud neck, and second, the pulling action of myosin type II molecules (Myo1p) anchored in the bud neck [470]. In a study from Higuchi et al. it was shown that an increase in RACF rates facilitated the inheritance of fitter mitochondria to the bud, and promoted prolonged replicative lifespan, a decrease of RACF had an opposite effect. This data supports the model that RACF serves as a “filter” that prevents the inheritance of less fit mitochondria to daughter cells [26]. It was also demonstrated that Sir2p, a lifespan regulator of the Sirtuin family [470], may act in part by modulating actin skeleton dynamics, as deletion of this gene decreased actin cable abundance, decreased RACF rates, and impaired segregation of reduced and oxidized mitochondria between mother and daughter cells; overexpression of this gene had the opposite effect [26]. In addition, the transcriptional repressor Sum1p was shown to counteract the impact of Sir2p on actin cytoskeleton dynamics and mitochondria inheritance [202].

**Figure 6:**
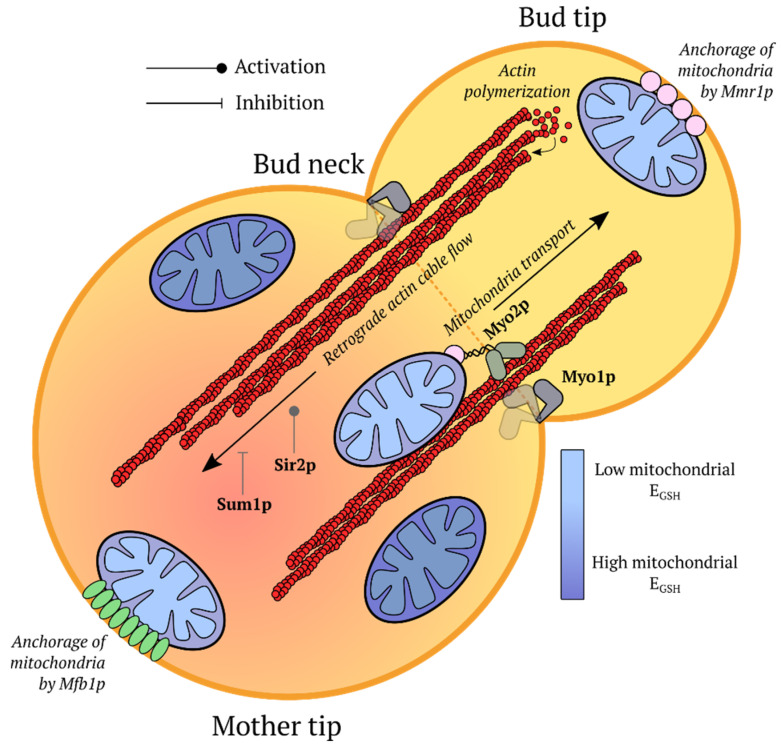
Inheritance of mitochondria in yeast cells. During division daughter cells on average inherit mitochondria with lower glutathione redox potential (*E_GHS_*) than mother cells, these mitochondria are anchored in the bud tip by Mmr1p. Mitochondria are transported to the daughter cell via actin cytoskeleton in the direction opposite to retrograde actin cable flow (RACF), which serves as a “filter” that prevents inheritance of less fit mitochondria to the bud. However, the mother cell retains a pool of high-functioning mitochondria anchored by Mfb1p in the mother tip [25,26,27,202,470].

Proteins that anchor mitochondria such as Mmr1p and Mfb1p also play a crucial role in proper mitochondrial distribution between the mother and the bud [25,27]. Mmr1p is required for myosin-dependent transport of mitochondria along the actin cables and anchors them in the bud tip [469]. In contrast to WT cells that always produce rejuvenated daughter cells, in Mmr1p deletion mutants, mitochondria quality control was disrupted which led to the generation of two subpopulations of daughter cells: long-lived cells that inherited fit mitochondria and short-lived cells that inherited less fit mitochondria [25]. The other protein, Mfb1p, is essential for retaining some high-functioning mitochondria in the mother cell by anchoring them in the mother tip. Deletion of Mfb1p caused a disproportionate loss of high-functioning mitochondria from mother cells and led to a decline in replicative lifespan (Figure 6) [27]. 

### 5.5. Redox Regulation of Transport Proteins in Microorganisms

**Figure 7:**
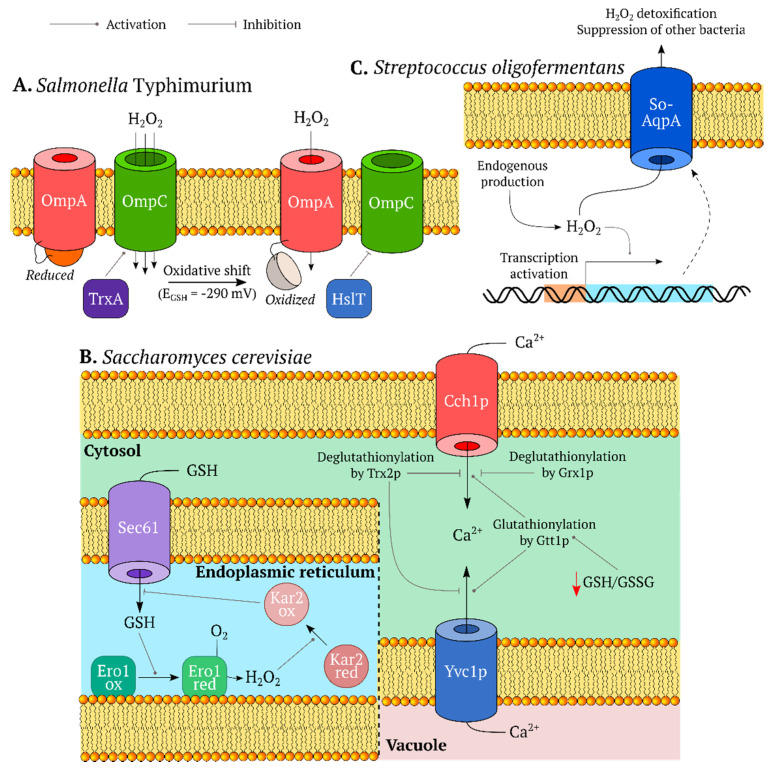
Many membrane transport systems are regulated in a redox-dependent manner. (**A**) In *Salmonella*, an oxidative shift in the *E_GSH_* value leads to a rapid decline in membrane permeability due to redox-dependent closure and opening of OmpC and OmpA proteins, respectively [258]. (**B**) Blue background: transport of glutathione into the endoplasmic reticulum (ER) in *S. cerevisae*. Glutathione is transported into the ER via Sec61 membrane protein complex. Glutathione transport is regulated by a negative feedback loop: (1) reduced glutathione (GSH) enters the ER via concentration gradient; (2) in the ER GSH induces activation of Ero1 which produces H_2_O_2_; (3) Kar2 protein is oxidized by H_2_O_2_ and in oxidized form blocks GSH transport into the ER [43]. Green background: Regulation of calcium channels in *S. cerevisae***.** Oxidation of the cellular glutathione pool leads to activation of Cch1p and Yvc1p calcium channels by glutathionylation. Deglutathionylaton is required for inactivation of these channels [209,211]. (**C**) So-AqpA of *S. oligofermentans* is involved in transport of H_2_O_2_ and expression of this gene is up-regulated in the presence of H_2_O_2_. So-AqpA promotes detoxification of endogenously produced H_2_O_2_ and enhances competitiveness of *S. oligofermentans* [263]. Abbreviations: *E_GSH_*—glutathione redox potential; GSSG—oxidized glutathione.

Several research groups conducted studies devoted to various transport proteins in microorganisms which turned out to be regulated by redox-sensitive mechanisms [43,209,211,258,263]. By recording *E_GSH_* using roGFP in catalases and peroxidases deprived *S. Typhimurium* after H_2_O_2_ addition, van der Heijden et al. discovered that at a potential of approximately −290 mV a switching point was observed after which the H_2_O_2_ influx rapidly dropped to ~8% and ~3% of initial values in log- and stationary-phase bacteria, respectively [258]. This change was too fast to be explained in terms of alteration of the membrane composition and subsequent knockout studies revealed that the OmpC and OmpA proteins are responsible for oxidant transport at different redox states of the cell. Detailed investigation led to a model in which disulfide bond formation in the periplasmic domain of OmpA results in its opening while OmpC undergoes a shutdown in oxidative conditions which is regulated in a more sophisticated way [258]. Elimination of the possible interaction partner HslT did not affect H_2_O_2_ influx before, but dramatically increased it after the switch. In contrast, TrxA mutants demonstrated abnormally low OmpC permeability at low *E_GSH_* (Figure 7A). The authors hypothesize that this regulation might be important for decreasing substance flow through the membrane in oxidative conditions such as those during the interaction with the host cells [258]. Therefore, HslT may be a perspective therapeutic target and HslT deficient cells were indeed more susceptible to cefotaxime application after incubation with macrophages [258]. 

Chandel et al. showed that the yeast calcium channels Cch1p, localized in the plasma membrane, and Yvc1p, localized in the vacuolar membrane, become activated by a variety of stimuli that promote glutathione oxidation. According to their data, a change of redox status of the glutathione pool induced opening of calcium channels via post-translational modification of cytoplasmically exposed cysteine residues by glutathionylation. The authors reported that glutathione S-transferase Gtt1p was responsible for glutathionylation of both Yvc1p and Cch1p. The opposite process, degluthathionylation, was mediated by thioredoxin Trx2 for vacuolar calcium channel and by Trx2 and glutaredoxin Grx1p for plasma membrane calcium channel (Figure 7B) [209,211]. 

In another study Ponsero et al. demonstrated that protein complex Sec61, known to translocate proteins in the ER [471], was responsible for glutathione import into the ER, and suggested a mechanism of regulation of this process [43]: (1) GSH enters the lumen of the ER through Sec61 via the concentration gradient as the concentration of GSH is higher in the cytoplasm than in the ER [43] (2) GSH induces activation of the Ero1 enzyme [472], that is involved in oxidative protein folding, and is known to produce H_2_O_2_ as a byproduct of its reaction [473]; (3) H_2_O_2_ induces oxidation of the ER-resident protein Kar2 [472] which in its oxidized form inhibits glutathione transport through Sec61 (Figure 7B). Thus, glutathione equilibrium in the ER is maintained via a negative feedback loop, as GSH induced activation of Ero1 eventually leads to inhibition of glutathione import [43]. 

Tong et al. chose the bacteria *Streptococcus oligofermentans* as their model system. These bacteria are known to produce H_2_O_2_ in aerobic conditions but, surprisingly, lack catalase—an enzyme responsible for H_2_O_2_ degradation. Thus, they need an alternative mechanism for detoxification of self-produced H_2_O_2_. The authors showed that the aquaporin of *S. oligofermentans* So-AqpA facilitated transmembrane H_2_O_2_ diffusion and transcription of the So-AqpA gene was up-regulated by elevation of H_2_O_2_ concentration. Furthermore, the presence of fully functional So-AqpA in the membrane of *S. oligofermentans* allowed the bacteria to inhibit growth of colonies of another species—*Streptococcus mutans*. The authors hypothesized that aquaporin So-AqpA might have several functions in bacteria: first, it is essential for the removal of excess H_2_O_2_, and second, it promotes the competitiveness of *S. oligofermentans* (Figure 7C) [263].

### 5.6. Redox Processes during Pathogenic Bacteria and Host Interaction

The interplay of redox processes that occur in pathogenic bacteria and the cells of the host represent a separate field of research interest, as deciphering of how these microorganisms orchestrate their responses against the defense machinery of infected cells and/or switch between various metabolic states for the propagation of their life cycle paves the way for the development of novel treatment strategies. *Chlamydia* spp. are obligate intracellular pathogens that demonstrate a biphasic developmental cycle represented by extracellular and intracellular forms called elementary and reticulate bodies (EB and RB) [474]. The conversion of condensed, osmotically resistant EB to osmotically sensitive, metabolically active RB proceeds in the inclusion vacuole after penetration into the host cell and this process is accompanied by significant redox rearrangements that involve several cysteine-rich outer envelope membrane proteins [475,476], in particular, the chlamydial major outer membrane protein which is highly cross-linked in EB and reduced in RB [477]. Wang et al. established that the oxidation state of roGFP1 expressed in the cytosol, mitochondria, or ER of CF15 cells did not demonstrate any significant changes indicating that the redox processes in *Chlamydia* do not influence the GSH metabolism of the host [234]. Under conditions of H_2_O_2_ or dithiothreitol treatment the infected cells were characterized by the same dynamics as the control ones. Therefore, the redox buffering capacity was not disturbed either. When the sensor was expressed in bacteria it underwent gradual oxidation from 16 to 18 h post infection (hpi) which correlated with the degree of major outer membrane protein cross-linking [234]. These data suggest that *Chlamydia* regulate their redox state at the individual cell level. Moreover, because *Chlamydia* switch from RB to EB before leaving the cell asynchronously, the redox status of the inclusion lumen becomes a subject of interest in the context of possible interplay between bacterial cells at different developmental stages. 

In another study, Nandy et al. addressed the question of Mtb (H37Rb strain) redox homeostasis in lipid-rich niches [252]. This question is of high medical relevance because in tuberculosis patients Mtb is capable of residing in caseating granulomas which constitute a complex environment formed after the necrosis of the host tissues [478]. The model system used in this work was represented by triglyceride-rich 3T3L1 adipocytes and their precursor preadipocytes serving as control cells [252]. Transcriptome studies revealed that Mtb in necrotic adipocytes (Mtb^A^) were characterized by suppression of iron uptake genes that are part of the IdeR regulon which suggests increased iron availability in this niche, confirmed by plasma mass spectrometry. Ferritin expression was enhanced both in granulomas of Mtb infected mice and RAW264.7 cells treated with oleic acid linking the lipid and iron metabolism in the tested systems [252]. Interestingly, a set of redox-active genes (*katG, ctpC, whiB3, cysD, cysN*) was upregulated in Mtb^A^ indicating that bacteria undergo oxidative stress under these conditions [252]. However, it is known that oxidative stress leads to dismantling of the IdeR Fe-S cluster which activates members of the regulon [479], a feature not observed in this study. The *E_MSH_* values registered with Mrx1-roGFP2 in Mtb^A^ and the control cells were similar, while CuOOH treatment induced a significantly smaller response in the former case [252]. When plated in the presence of CuOOH or PLB, the growth of Mtb^A^ cells was violated to lesser degree. A similar result was observed for bacterioferritin lacking cells that are sensitive to elevated iron levels and oxidative stress, significant growth impairment was observed only in preadipocytes. Finally, the external addition of oleic acid shifted *E_MSH_* of Mtb to lower levels leading to a more-reducing cytosol [252]. All these data together support the hypothesis that Mtb are capable of effectively coping with oxidative stress in a lipid-rich environment due to a set of mechanisms dependent on fatty acids supply and host ferritin presence. 

**Figure 8:**
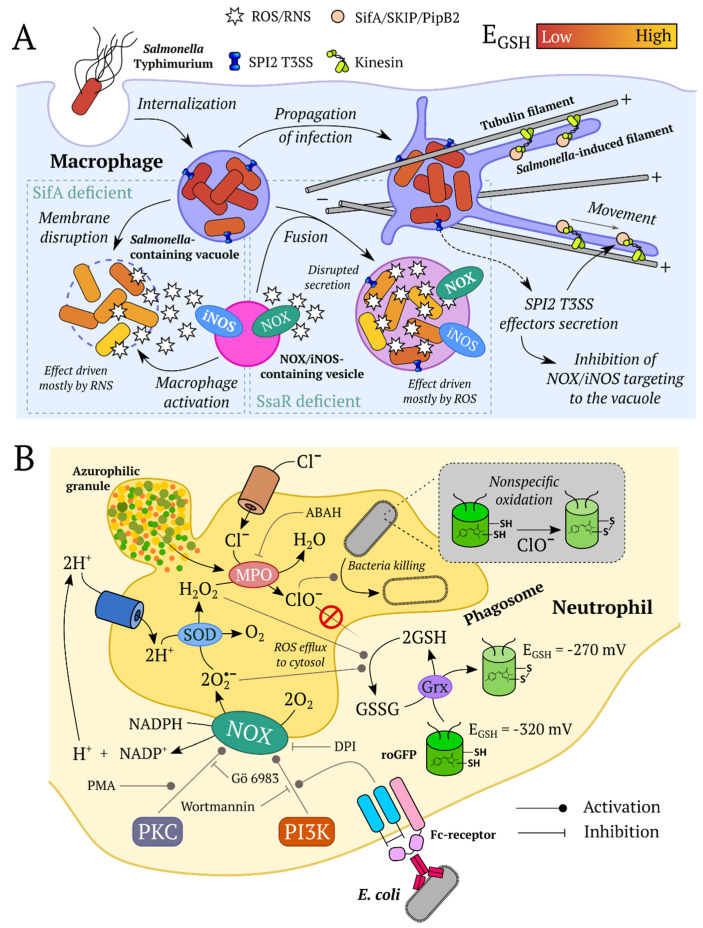
The picture represents selected redox processes during bacteria and immune cells interactions. (**A**) When residing in macrophages, *Salmonella* Typhimurium is capable of avoiding redox stress due to injection of *Salmonella* pathogenicity island 2 (SPI2) effectors via type 3 secretion system (T3SS) into the host cytosol. These proteins disrupt the correct localization of reactive oxygen/nitrogen species (ROS/RNS) generating machinery [259]. (**B**) Any implementation of roGFP-based probes in neutrophilic phagosome faces significant difficulties since high HOCl concentration in this compartment leads to a nonspecific oxidation of the sensors. Interestingly, it seems that myeloperoxidase (MPO)-generated oxidants do not alter cytosolic glutathione redox potential (*E_GSH_*) as revealed by roGFP2. When experiments with neutrophil activation are planned, it is important to take into account that phorbol 12-myristate 13-acetate (PMA) treatment and bacteria exposure stimulate this process via different signaling pathways [51,239]. Abbreviations: DPI—diphenyleneiodonium; GSH—reduced glutathione; GSSG—oxidized glutathione; iNOS—inducible NO synthase; MPO—myeloperoxidase; NOX—NADPH oxidase; PKC—protein kinase C; PI3K—phosphoinositide 3-kinase; SOD—superoxide dismutase.

Some studies exist that investigated the redox metabolism of pathogenic bacteria in macrophages and macrophage-like cells using redox sensors. In particular, Loi et al. established that after 1 h of THP-1 cells infection with *S. aureus* expressing Brx-roGFP2 the degree of sensor oxidation was calculated as 87% [89]. During infection, *S. Typhimurium* resides in the bacteria-containing vacuole (SCV) and utilizes the type III secretion system (T3SS) to inject effector proteins encoded in *Salmonella* pathogenicity island-2 into the host cell [480,481]. Experimental evidence exists that the described mechanism participates in ROS and reactive nitrogen species (RNS) evasion strategies [482,483]; however, this statement was questioned in a work by Aussel et al. [484]. The comparison of roGFP2 oxidation degrees revealed that *S. Typhimurium* mutants with disrupted T3SS assembly (*ssaR*) experienced more pronounced redox stress than WT bacteria in THP-1 cells which was not attributed to decreased overall oxidant production in the system (Figure 8A) [259]. Detailed investigation using bone marrow derived macrophages from *gp91phox*^−/−^ and *iNOS*^−/−^ (disrupted phagosomal ROS and RNS generation, respectively) mice established that the observed shifts in the signal of the sensor were attributed to ROS exposure [259]. These data suggested that *ssaR* mutants have an impaired ability to prevent the correct localization of the host defense machinery required for ROS/RNS delivery to SCV. This hypothesis was supported by the fact that *sifA* mutant bacteria capable of entering the host cytosol due to disrupted SCV integrity experienced significantly higher oxidative stress compared to WT cells [259]. Interestingly, implementation of diphenyleneiodonium (DPI) and L-NMMA established that the source of oxidative stress for cytosolic bacteria in THP-1 cells was inducible NO synthase (iNOS) [259]. The authors argue that the failure to register oxidative stress in *Salmonella* pathogenicity island-2 deficient bacteria in the previous study can be explained in by insufficient sensitivity of the *ahpCp*-GFP transcription reporter [259]. 

Bhat et al. observed that Mtb intrusion to RAW264.7 cells increased both the average NADH/NAD^+^ ratio and its variance as registered by Peredox-mCherry [250]. The authors believe that this shift results from a general slowdown of metabolism and according to the existing knowledge, it might help bacteria to confer antimycobacterial compounds [485]. Moreover, the increased variance suggests that individual cells occupy different metabolic niches, which is of interest in understanding drug resistance. The activation of RAW264.7 cells by interferon-γ treatment elevated the ratio of the probe [250], an important observation in light of the fact that in contrast to naïve cells activated macrophages can resist intracellular growth of mycobacteria [486]. Implementation of the selective iNOS inhibitor, L-NG-Nitroargininemethylester, significantly mitigated the effect of stimulation suggesting an important role for NO in adjusting the Mtb NAD(H) redox pool [250]. The phenomenon of metabolic heterogeneity induced by pathogen-host interaction deserves special attention. In contrast to the extracellular culture, H37Rv Mtb internalized into THP-1 cells can be divided into three subpopulations that differ by Mrx1-roGFP2 oxidation degree [52]. At 24 hpi a group of bacteria with reduced cytosol emerges, which is followed by the appearance of an “oxidized” fraction at 48 hpi and clearly detectible recovery for the next 24 h. The described trend is generally replicated in the RAW264.7 system [52]. Detailed investigation connected these shifts with sub-vacuolar localization of Mtb, namely the share of “oxidized” cells rapidly grew in the row of early endosomes, lysosomes and autophagosomes. In line with the data mentioned above, the reductive recovery phase was compromised when RAW264.7 cells were activated, while iNOS inhibition led to a significant decline in the size of the “oxidized” subpopulation [52]. Together these observations suggest that interaction with the host might itself generate cells that are metabolically resistant to existing treatment strategies even on the background of a drug-sensitive genotype, which will be discussed in the next subsections. 

Investigation of redox processes following phagocytosis in neutrophils, highly specialized immune cells possessing sophisticated bacteria-killing machinery, is of special medical interest. Cytosolic *E_GSH_* in PLB-985 cells measured by roGFP2 using a plate reader shifted from −320 mV to a noevel steady-state of approximately −270 mV after both phorbol 12-myristate 13-acetate (PMA) stimulation and *E. coli* exposure; however, the oxidation rate was 6 times slower in the latter case (Figure 8B) [51]. The observed slowdown resulted from phagocytosis dyssynchrony as revealed by imaging of individual cells with fluorescence microscopy. On the single-cell level, probe oxidation was completed within several minutes after bacteria internalization indicating that this process is the limiting step of the discussed shift [51]. Interestingly, in contrast to NOX2 inhibition with DPI, ABAH-induced MPO inactivation did not influence the steady-state after both stimuli suggesting that HOCl and its derivatives do not play the key role in the observed dynamics (Figure 8B) [51]. This result is quite exciting, considering that chloramines are capable of crossing biological membranes [487]. It is known that NOX2 can be activated by different routes including PKC- and PI3K-mediated signaling [488,489,490]. Implementation of Wortmannin, a PI3K inhibitor, to this system diminished the *E. coli*-induced shift while the response to PMA was not affected. The PKC inhibitor Gö 6983 led to the opposite behavior [51]. These data clearly show that PMA treatment and *E. coli* exposure trigger redox alterations that are visually similar but different by their nature, which highlights the importance of correct experimental model selection in light of the fact that PMA is often implemented as a relatively simple model for immune cells activation (Figure 8B). The redox homeostasis of the phagosomal lumen deserves special attention. When expressed in *E. coli*, Grx1-roGFP2, roGFP2-Orp1, and roGFP2 reported fast activation within seconds after phagocytosis by PLB-985 cells [239]. Given that all these probes are capable of direct interaction with HOCl [94], the similar kinetics observed in all cases was most likely attributed to unspecific oxidation (Figure 8B). MPO inhibition, significantly mitigated the amplitudes of response, supporting this hypothesis [239]. It seems that deep investigation of processes inside the neutrophil phagosome requires implementation of specific probes which have to be developed in the future. Interestingly, when bacteria were incubated with PLB-985 cells, in which phagocytosis was inhibited by Cytochalasin D, no signal changes were registered indicating that *E. coli* experience oxidative stress only when internalized [239]. 

### 5.7. Redox Processes during Interactions of Pathogenic Microorganisms and Drugs

#### 5.7.1. P. falciparum

Malaria is a parasitic tropical disease transmitted by the bite of the female *Anopheles* mosquito. Although 120 *Plasmodium* species exist, only six of them are known to cause human diseases [491]. *P. falciparum* and *P. vivax* are the predominant pathogens with an estimated incidence of 207 million and 8.5 million cases respectively in 2016 [492]. From an epidemiological point of view Africa remains the most problematic region since about 90% of global malaria morbidity and mortality are restricted to this area [493]. The human phases of the malaria life cycle start with the transfer of sporozoites into the blood from the vector insect after which they migrate to the liver where exo-erythrocytic schizogony proceeds [494]. Next, the mature merozoites enter red blood cells (RBCs) to multiply via the ring and the trophozoite stages. Several cycles of asexual replication lead to a significant increase in the parasitic burden and culminate with the emergence of female and male gametocytes that are subsequently transmitted to a mosquito during a blood feed [494]. At this point the sexual part of *Plasmodium* life cycle begins which produces new sporozoites. Despite the development of preventive and treatment approaches, malaria remains a major global death challenge. In 2017, ~435,000 deaths were attributed to this disease among which children under five years constituted more than 60% [493]. Currently, artemisinin-based combination therapies are the treatment of choice; however, there is a rising concern about the emergence of *Plasmodium* strains that are resistant to artemisinin and its partner compounds [495]. 

*Plasmodium* spp. face both internal and external oxidative stress during the infection cycle, and experimental evidence supports a key role of GSH in maintaining the redox homeostasis of the parasite [496]. In particular, elevated production of H_2_O_2_ and hydroxyl radicals was detected in infected erythrocytes [497]. When residing in erythrocytes, the parasite degrades hemoglobin in its digestive vacuole with a set of peptidases, wherein heme and hemin/hematin are generated during this process as redox-toxic byproducts. To avoid cellular damage, these compounds are biomineralized in the form of hemozoin crystals and stored within the digestive vacuole; however, certain leakage to other compartments occurs since the described mechanism is not absolutely efficient [498]. It is accepted that the parasite utilizes GSH to detoxify free hemin/hematin, preventing undesired ROS production [496]. Moreover, modern therapeutic approaches most likely rely on altering the redox state of *Plasmodium* cells to some degree. Several anti-malarial drugs, namely methylene blue, quinoline, and artemisinin-based compounds, were found to accumulate in the digestive vacuole and inhibit hemozoin formation [498,499]. Furthermore, chloroquine and amodiaquine are thought to interfere with GSH-mediated hemin/hematin neutralization [500]. In addition, artemisinin derivatives contain an endoperoxide bond that generates alkylating free radicals under activation by intracellular iron [501] and, finally, methylene blue is a known redox cycler [502] and an inhibitor of *Plasmodium* GSSG reductase [503]. 

All the data mentioned above clearly highlights the importance of measuring the redox changes in *Plasmodium* for understanding its biology and paving the way for the development of novel medications. In 2015 Rahbari et al. reviewed the approaches for investigation of oxidative stress in parasitic microorganisms [504]. The authors emphasized the advantages of GEFIs in this field and discussed the first implementation of the Grx1-roGFP2 probe in *Plasmodium* [223]. Subsequently, this sensor was directly compared with ThiolTracker^TM^ Violet (a marker for the thiol status) and CM-H_2_DCFDA (a marker for general oxidative stress) in the same system [225]. All methods reported consistent results; however, compared to ThiolTracker^TM^ Violet, Grx1-roGFP2 provided increased sensitivity in some situations. The sensor was proven to be suitable for both confocal laser scanning microscopy and plate readers. Interestingly, when total thiol and GSH levels were measured by chemical approaches, the corresponding values tended to shift in Grx1-roGFP2 expressing cells indicating a possible influence on the redox metabolism [225]. This hypothesis was rejected in a further study with the use of a genome-integrated probe, which suggested that the exposure to the selective marker antifolate WR99210 was responsible for the observed differences [73]. In addition, a superfolder variant of roGFP2 was engineered in this study which provided higher fluorescence levels with a similar dynamic range (hGrx1-roGFP2: 4.23; sfroGFP2: 4.56) [73]. Despite not having the sensory domain, unfused sfroGFP2 still showed satisfactory kinetic properties, apparently due to the high Grx concentration in *Plasmodium* cytosol. In this study the best time window for imaging was located between ~25 hpi and ~35 hpi, which corresponded to mature trophozoites. Although visible, fluorescence in ring-stage parasites and merozoites was too low for reliable measurements [73]. 

When targeted to various compartments, Grx1-roGFP2 allows for estimation of the steady-state *E_GSH_*. The basal cytosolic value in *P. falciparum* (approximately −315 mV) obtained by this approach was found to be more reducing than suggested in previous works [505]. Interestingly, the ratio of the sensor was significantly lower in a chloroquine-resistant (Dd2) strain compared to a chloroquine-sensitive (3D7) one [223]. Moreover, the maximum response amplitude was bigger for 3D7 cells and the same H_2_O_2_ concentration triggered more pronounced oxidative shift in their case. These observations are in line with earlier reports about higher concentrations of total GSH in the Dd2 strain, which might lead to enhanced reductive power [506]. As measured by pHluorin, the pH values in the cytosol, apicoplast and mitochondrial matrix of *P. falciparum* blood stages were ~7.16, ~7.12 and ~7.37. GSH redox potentials of −267 mV and −329 mV for the apicoplast and the mitochondrion, respectively, were calculated [16]. 

GEFIs have also been used for direct measurement of H_2_O_2_ in *P. falciparum.* When expressed in trophozoites, both HyPer-3 and roGFP2-Orp1 demonstrated dose-dependent responses to external oxidant addition; however, the former probe exhibited higher sensitivity and faster oxidation-reduction dynamics [226]. These differences may be due to the need in inter-domain exchange with oxidative equivalents for roGFP2-Orp1 as well as to unequal interaction of the sensors with the cellular reductive systems. To investigate whether erythrocytes provide antioxidant defense for the parasites, their membranes were destroyed by saponin implementation. Under these conditions, a pronounced increase in the rate of roGFP2-Orp1 response was observed while in case of HyPer-3 the amplitudes of the response became elevated [226]. Therefore, *P. falciparum* indeed becomes more susceptible to external oxidation after removal of the host cell. The measured maximum response amplitudes within intact RBCs were 5 and 7.3 for roGFP2-Orp1 and HyPer-3. The corresponding values after the lysis of erythrocytes rose to 5.5 and 12 [226]. Despite the aforementioned advantages, imaging with HyPer-3 in this system was associated with a number of pronounced drawbacks [226]. First, the probe demonstrated an approximately 6-fold lower expression rate compared to a roGFP2-based sensor. Second, in control studies it was established that the SypHer and HyPer-3 ratios overlapped only at pH 7.0–7.5, which might hamper signal normalization at pH values outside this range. When targeted to the mitochondrial matrix, roGFP2-Orp1 was capable to register dose-dependent responses to external H_2_O_2_; in addition, its subsequent reduction occurred faster than for the cytosolic version [17].

All studies in which redox sensors were expressed in *P. falciparum* were related to screening of the effects induced by antimalarial compounds. The mode of influence on *E_GSH_* was investigated for a large set of drugs: methylene blue [223], chloroquine [16,223,225], amodiaquine [223], quinine [223], mefloquine [73,223], artesunate [73,223], artemether [73,223], artemisinin [16,223,225], fosmidomycin [16], nitrofurazone [16], rotenone [16], 2-desoxyglucose [16], ellagic acid [16], malarone [16], atovaquone [16,73], arylmethylamino steroid 1o [73], and lumefantrine [73]. In addition, some compounds were tested for their ability to promote H_2_O_2_ generation: amodiaquine [17], atovaquone [17], lumefantrine [17], primaquine [17], rotenone [17], 2-deoxyglucose [17], arylmethylamino steroid 1o [17], ML30427 [17], artemisinin [17,226], artemether [17,226], artesunate [17,226], chloroquine [17,226], quinine [17,226], and mefloquine [17,226]. Generally, the experimental protocols included the registration of fluorescent signals as functions of incubation time. Thus, short- (minutes), medium- (4 h) and long-term (24 h) effects were studied. For many of the drugs, a common trend of oxidation induction at longer times was observed. However, in most cases, the redox shifts did not reach statistical significance. Interpretations from these studies must be carried out with reasonable caution. The redox cycler methylene blue and ellagic acid derivatives flavellagic acid and corulleoellagic acid were shown to induce pronounced and immediate responses of the sensors during direct interaction in vitro [223,226]. Therefore, it might be difficult to rule out a similar mode of action in vivo. The fact that artemisinin-based compounds as well as quinolines only trigger oxidative shifts on longer time scales is not really surprising [16,223]. In the former, the activation of the endoperoxide bond results in the production of a single radical per drug molecule; therefore, they most likely cannot act as a source of any potent and rapid redox stress. However, these compounds enter the food vacuole and inhibit hemozoin formation, resulting in pro-oxidative byproducts that require time for accumulation. In several studies, the ability of artemisinin and atovaquone to affect the mitochondrial membrane potential was shown [507,508]. The lack of pronounced shifts in mito-*E_GSH_* under their administration might be attributed to the specificities of the experimental settings [16]. In trophozoites, the asexual stages of the parasites, the citric acid cycle is unlikely to play a major metabolic role in contrast to in late gametocytes [509]. One more interesting observation is that the combination of glycolysis and ETC inhibition by simultaneous addition of 2-desoxyglucose and rotenone led to the disruption of intercellular thiol metabolism as a consequence of oxidative stress [16]. It should be noted that for many compounds the effects were more pronounced in the 3D7 strain compared to Dd2 in accordance with the stronger reductive power in the latter [223]. Finally, in a single study roGFP2-Orp1 was implemented to investigate if antimalarial drugs alter the response of *P. falciparum* to external H_2_O_2_ addition [226]. It was shown that pre-incubation of trophozoites with artemisinin, chloroquine and quinine decelerated the initial oxidation rate, this was most pronounced with quinine. 

Besides characterization of the redox components in the mechanisms of action of known antimalarial chemicals, imaging with Grx1-roGFP2 can complement research aimed at deciphering the molecular mechanisms underlying the toxic properties of novel drugs. G6PD deficiency confers increased sensitivity to oxidant compounds and food components on RBCs, making them susceptible to phagocytic removal due to more pronounced deposition of haemichromes, products of hemoglobin destruction [510,511,512]. In a large set of studies, it was suggested that this phenotype provides enhanced resistance to malaria via stimulation of the phagocytosis of the early ring stages of the parasites [513]. Earlier, 3-[substituted-benzyl]-menadiones (benzylMD) were identified as potent chemicals that affect the redox homeostasis of *Plasmodium* infected erythrocytes and, therefore, deserve attention as promising candidates for novel treatment programs [514,515]. Bielitza et al. demonstrated that after internalization, benzylMD becomes subjected to in vivo benzylic oxidation that generates a derivative that is an extremely effective substrate of *P. falciparum* glutathione reductase [224]. Moreover, the product of this enzymatic reaction can be reduced by met-hemoglobin. Therefore, a redox cycle emerges which on the one hand consumes cellular NADPH and on the other disrupts normal hemozoin formation. As described above, this eventually results in enhanced ROS accumulation and the formation of haemichromes on the membranes of infected ring-stage cells targeting them for phagocytosis [224]. With the use of Grx1-roGFP2 the authors showed that benzylMD significantly affected *E_GSH_* in the cytosol of *P. falciparum* after 4 h incubation and compared to artemisinin and quinolone, the shifts triggered by this compound developed more rapidly [224]. To conclude, benzylMD seems to be the first chemical that metabolically mimics the natural mechanism for malaria protection, namely G6PD deficiency.

#### 5.7.2. Trypanosomatidae

Trypanosomatidae is a big family of unicellular organisms that includes important pathogenic representatives among which *Trypanosoma brucei*, *T. cruzi* and *Leishmania* spp. cause African sleeping sickness, Chagas disease and leishmaniasis, a group of neglected tropical diseases [516]. As these obligate parasites circulate between vertebrate hosts and insect vectors, they shift between phenotypically and metabolically distinct forms, and each processes unique features in their life cycle [517]. While all growth and proliferation stages of *T. brucei* in mammalian hosts proceed in the extracellular space [518], *T. cruzi* and *Leishmania* spp. are characterized by internalization into the host cells at some step of development. When *Leishmania* spp. enter macrophages, they reprogram their functioning and reside in the parasitophorous vacuoles after conversion to the amastigotic form [519]. Fibroblasts, macrophages, epithelial cells, and smooth muscles serve as vessels for *T. cruzi* infection [516,517,520]. All the diseases caused by the representatives of Trypanosomatidae are life-threatening and inflict significant suffering on infected patients. Therefore, they constitute a major economic and social burden. In particular, the penetration of tripanosomes into the brain is accompanied by serious sleep cycle disruptions, paralysis, and progressive mental deterioration [517]. Despite prevention and treatment efforts, the corresponding incidence rates were estimated to be ~50,000, ~10,000, and ~2,000,000 new cases per year for Chagas disease, sleeping sickness, and visceral/cutaneous leishmaniasis, respectively [521]. In contrast to the human pathogens *T. brucei rhodesiense* and *T. brucei gambiense*, *T. brucei brucei* is the etiological agent of the cattle disease Nagana which leads to ~3000,000 deaths and financial losses in the range of $6–12 billion per year [227]. At present meglumine antimoniate, sodium stibogluconate, amphotericin B deoxycholate, pentamidine, miltefosine, paromomycin, benznidazole, posaconazole, eflornithine, nifurtimox, melarsoprol, and suramin are used for clinical treatment of the discussed diseases [517]. However, implementation of these chemicals has several limitations among which are the emergence of resistant strains, toxicity problems, as well as limited stage- and parasite subspecies-specific efficacy [522,523]. Thus, the search for novel therapeutic approaches represents an important goal in the field of Trypanosomatidae research. In several studies, Grx1-roGFP2 was implemented for clarification of the mechanisms which underlie the functioning of perspective drugs.

**Figure 9:**
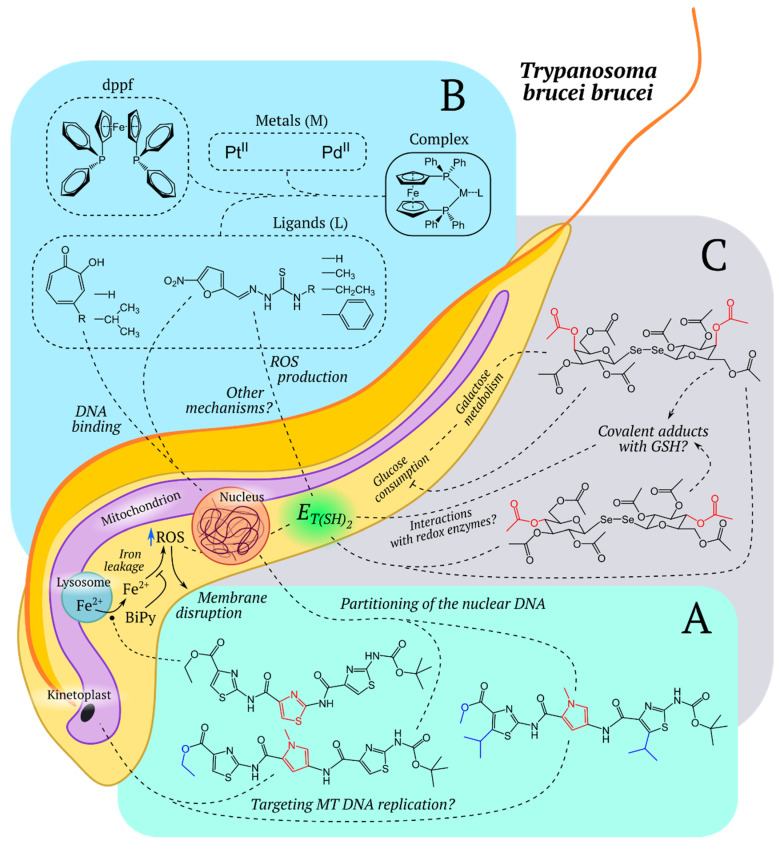
Several anti-trypanosomal compounds were tested for their ability to affect redox homeostasis in *Trypanosoma brucei brucei*. Interestingly, in many cases, small variations in the molecular structure of a drug (highlighted in color) lead to a dramatic change in the mechanism of its functioning. (**A**) Anti-trypanosomal tri-thiazoles linked by amide [227,231]. (**B**) Anti-trypanosomal organometallic compounds [229,230]. (**C**) Anti-trypanosomal sugar diselenides [228]. Abbreviations: dppf—1,1′-bis(diphenylphosphino) ferrocene; *E_T(SH)2_*—trypanothione redox potential; GSH—reduced glutathione; MT DNA—mitochondrial DNA; ROS—reactive oxygen species.

It is known that some analogues of the DNA minor groove binders distamycin and netropsin possess anti-trypanosomal activities [524,525]; however, the corresponding mechanisims of action were not investigated in detail. Franco et al. synthesized a set of bi- and tri-thiazoles linked by amide and tested their cytotoxicity against *T. b. brucei* [227]. Interestingly, in general the second group was characterized by smaller EC_50_ values and higher selectivity indexes towards murine macrophages. Moreover, it was shown that the hydrophobicity of the compound correlated with increased biological activity [227]. When the most potent candidate, N-Boc trithiazole ester, was investigated in detail, propidium iodide staining revealed that the drug caused disruption of cellular membranes suggesting that it might act in a similar way to defensins which are capable of pore formation. In addition, the ratio of Grx1-roGFP2 indicated that there was a more oxidized glutathione pool in the treated parasites. Imaging with the MitoTracker and LysoTracker fluorescent probes showed that N-Boc trithiazole ester affected the structure of the lysosome in some way, since discrete staining of the organelle was lost in these conditions. Finally, PhenGreen SK diacetate gave data showing elevated levels of free iron in treated parasites [227]. In light of the above, the authors hypothesized that N-Boc trithiazole ester disrupted the integrity of the lysosomal membrane with consequent release of free iron into the cytosol which generated ROS via redox cycling and, therefore, promoted pronounced lipid oxidation (Figure 9A). In line with that, the metal chelator BiPy was capable of diminishing the toxic effects of N-Boc trithiazole ester, supporting the described reasoning [227]. In further work, another set of candidates was investigated which differed by the inclusion of an N-methyl pyrrole as the central ring [231]. Such modification markedly enhanced both anti-trypanosomal activity and selectivity; thus, the selectivity indexes were found to be > 10169 and 5560 for the two best chemicals. Interestingly, another mechanism underlies their functioning since propidium iodide staining failed to visualize any significant disruption of the cellular membrane, and no redox shifts were detected with Grx1-roGFP2 (Figure 9A). Additional experiments with a synchronized culture suggested that these compounds act via inhibition of kinetoplast DNA replication without affecting its integrity [231]. 

In the context of rising interest towards organometallic compounds which might provide beneficial properties due to metal-ligand synergism, the representatives of this group were tested for their ability to affect *T. cruzi* metabolism [526,527]. The inspiring results obtained in these studies, encouraged Rivas et al. to characterize the biological mode of action for 1,1′-bis(dipheny1phosphino) ferrocene hexafluorophosphate compounds [M^II^(Tropolone)(dppf)](PF_6_) and [M^II^(Hinokitiol)(dppf)](PF_6_), where M corresponds to Pd or Pt [229]. All the complexes turned out to be more cytotoxic compared to free ligands and the Pd-based versions showed lower EC_50_ values at the cost of worse selectivity indexes. Ferrocenes are capable of undergoing one electron oxidation which in biological systems may lead to ROS production via Fenton-type chemistry [528,529], but none of the tested chemicals induced detectible shifts in the Grx1-roGFP2 ratio suggesting that their mechanism of action does not markedly disturb the thiol homeostasis of the parasites [229]. M-dppf-ligand complexes were also shown to interact with DNA as revealed by the ethidium bromide displacement assay. Therefore, the authors concluded that DNA might be the molecular target of these chemicals (Figure 9B) [229]. In another work, 5-nitrofuryl containing thiosemicarbazones, analogues of the anti-trypanosomal drug Nifurtimox, were tested as possible ligands for the same organometallic cores [230]. In these compounds the nitro group can be reduced to a nitro anion by trypanosomal enzymes, which underlies their ability to promote oxidative stress in vivo [530]. The cytotoxicity of the synthesized candidates was investigated in *T. cruzi* trypomastigotes and bloodstream *T. brucei* model systems [230]. Interestingly, cyclic voltammetry experiments showed that the complexes were more prone to reduction than the free ligands. Besides the fact that the best versions were also proven to act as DNA binders, the spin trapping agent DMPO revealed the production of free radicals in drug-treated parasites (Figure 9B). Moreover, an increase in oxygen consumption was detected in *T. cruzi* epimastigotes incubated with the studied complexes further supporting the induction of redox cycling processes in the cells [230]. However, the redox ratio of Grx1-roGFP2 was altered in the presence of only three candidates. Surprisingly, one of them even caused apparent reductive stress, the basis for which is not clear. Since different versions showed non-equal ability to modulate the redox state of GSH pool, it might be that selective molecular targets and/or activation pathways exist for the tested complexes [230]. 

In order to simultaneously target both glycolysis and redox homeostasis of *T. b. brucei*, Franco et al. investigated the anti-trypanosomal effects of sugar diselenides [228]. One of the compounds (named 15) was shown to reduce the ability of the parasites to consume glucose, which, was not attributed to uptake inhibition. The authors hypothesized that this mode of action relies either on the interaction between the drug and glycolytic enzymes, or on possible interference of the chemical with galactose metabolism, which alters glucose metabolism in response [228]. Compounds 13 and 15 also shifted the ratio of the Grx1-rpGFP2 sensor to more oxidized values suggesting the presence of a redox component in their mode of action. When drugs were mixed with the purified probe in vitro, no signal changes were observed even in the presence of GSH confirming that they do not oxidize the protein directly and do not induce GSSG production. However, nuclear magnetic resonance studies revealed that both chemicals covalently reacted with GSH and N-acetyl-cysteine via the formation of a Se-S bond between the reactants. The resulting species potentially act as modulators of either redox homeostasis or sugar metabolism. In particular, the molecular mechanism action might include enzyme glycosylation as an initial step (Figure 9C) [228]. 

Although the members of the Trypanosomatidae family possess GSH and Trxs in their cells, they lack GSSG reductases and Trx reductases, therefore, the aforementioned systems depend on electron supply from trypanothione [531]. Trypanothione consists of two GSH molecules linked by spermidine and is involved in redox reactions mostly mediated by tryparedoxin (Tpx) which is a distant thioredoxin homolog. As in a typical GSH-based system, NADPH serves as the primary source of reductive equivalents for the active thiol groups with participation of trypanothione reductase as a coupling enzyme [531]. Recently, a sensor in which roGFP2 is fused with Tpx was developed and compared to Grx1-roGFP2 in the same conditions [90]. When the selectivity of reduced probes was investigated in vitro it was shown that Tpx- and Grx1-based versions responded faster towards oxidized trypanothione (TS_2_) and GSSG, respectively; however, they were capable of being oxidized by both substrates. Moreover, in contrast to Tpx-roGFP2 the oxidized sample of Grx1-roGFP2 reacted not only with its selective target GSH, but also with reduced trypanothione (T(SH)_2_) [76]. The higher reductive power of the latter might be attributed either to the intramolecular character of disulfide bond formation or to the fact that its thiols are more acidic by at least one pH unit compared to GSH [532]. In any case, it seems that in Trypanosomatidae Grx1-roGFP2 is not specific for *E_GSH_* and more likely reports the redox status of T(SH)_2_/TS_2_ system. 

Since catalase is absent in African trypanosomes, hydroperoxides are neutralized by 2-Cys-peroxiredoxins (cytosolic Px I and Px II, mitochondrial Px III) and by non-selenium glutathione peroxidase-type enzymes (cytosolic cPrx and mitochondrial mPrx) that rely on electron supply from T(SH)_2_ [533,534,535]. The cytosol of procyclic (the insect stage) *T. brucei* turned to be highly reducing as revealed by both sensors, and dose-dependent oxidation was observed in response to external H_2_O_2_ treatment [76]. The registered recovery rate was faster for the Tpx-based version. Interestingly, H_2_O_2_ at a concentration of 50 μM made both probes insensitive to a second dose of the same oxidant but not diamide, suggesting that cPrx becomes hyperoxidized in these conditions and, therefore, incapable of transferring oxidative equivalents to T(SH)_2_ [76]. When sensors were targeted to mitochondria, they revealed a more oxidizing trypanothione pool and their responses towards external oxidant addition were enhanced while the recovery rates were diminished. In contrast to cytosolic versions the mitochondrial probes were capable of detecting a second dose of H_2_O_2_, which might be attributed to the more limited access of this chemical to the compartment, or to the lower ability of mPrx to become over-oxidized [76]. 

When difluoromethylornithine, a drug interfering with T(SH)_2_ synthesis [536], was applied to the system, none of the sensors showed any significant shifts in the signal, although the total GSH and T(SH)_2_ concentrations became doubled and halved respectively [76]. Difluoromethylornithine, therefore, has no pronounced effect on the ratio of oxidized and reduced thiols. However, under its action the antioxidant capacity of the cells decreased as measured by the parameters of recovery after diamide stress. When Tpx was eliminated via RNA-interference, cytosolic Grx1-roGFP2 almost lost its ability to sense H_2_O_2_ addition indicating that in the absence of this enzyme trypanosomal peroxidases cannot detoxify the oxidant with the use of reductive equivalents coming from T(SH)_2_. Interestingly, the response was not completely lost in mitochondria [76]. Thus, it seems that another oxidoreductase is present in this compartment that is capable of equilibrating thiol peroxidases and the trypanothione redox buffer. In other experiments the authors also investigated the role of Pxs in maintaining redox homeostasis and demonstrated that mPrx is the main peroxidase that couples H_2_O_2_ and T(SH)_2_ [76]. 

While elimination of cPrx at the blood stage results in the lysis of *T. brucei* cells, mPrx seems to be dispensable during the mammalian part of the life cycle [533]. However, its physiological role in procyclic trypanosomes has not been fully investigated, even though this question is quite interesting since the mitochondrion is fully developed and metabolically active at this stage [537]. In a recent work Bogacz et al. demonstrated that the depletion of mPrx in procyclic *T. brucei* resulted in pronounced growth defects under standard culture conditions compared to blood stage parasites which were not affected by elimination of mPrx and Px III either separately or simultaneously [232]. MitoTracker Red staining revealed that the mitochondrial membrane potential was markedly diminished in mPrx-lacking cells supporting the role of this enzyme in mitochondrial redox metabolism. In the previous study, it was confirmed that mPrx participates in detoxification of exogenous H_2_O_2_ [76]. To establish its role under conditions of endogenous ROS production, antimycin A was applied to cells expressing Tpx-roGFP2 in either the cytosol or mitochondria [232]. The signal of the cytosolic version was only slightly affected while the mitochondrial probe showed intense oxidation. This mode of action was diminished in mPrx lacking cells, confirming that it acts as the main matrix enzyme responsible for H_2_O_2_ reduction. Interestingly, when parasites were grown in the presence of oxidative agents (MitoParaquat, antimycin A, or H_2_O_2_) depletion of mPrx had only a minor additional effect on proliferation and/or viability. The authors also demonstrated that the enzyme plays a significant role in adaptation to heat shock at both the blood and insect stages of the cell cycle [232]. To conclude, we think that the development of the Tpx-roGFP2 sensor is a very valuable addition to the modern palette of GEFIs and we are sure that it will significantly contribute to the investigation of Trypanosomatidae biology and anti-trypanosomal drug development. 

#### 5.7.3. Mycobacteria

Multidrug-resistant tuberculosis is a form of disease caused by strains tolerant to at least both isoniazid and rifampicin; while extensively drug-resistant strains demonstrate additional resistances to at least one second-line injectable antimicrobial compound (capreomycin, kanamycin, and amikacin) and any fluoroquinolone [538]. The search for novel tuberculosis therapeutic approaches is prioritized by international health institutions since treatment of extensively drug-resistant tuberculosis can last for up to two years [539] and management of a single case imposes a financial burden of €75,000 or more [540]. However, despite all the implemented efforts the rates of success after 36 months of treatment usually do not exceed 30% in the European Region of the World Health Organization [541]. Thus, as in the case of *Plasmodium* and *Trypanosoma* spp., GEFIs are capable of facilitating development and testing of novel redox-active compounds with potential anti-Mtb activity. Infection alters metal homeostasis in macrophages; in particular, Cu, which is capable of displacing metal cofactors of enzymes and demonstrates thiophilic properties [542], becomes enriched in phagosomes via ATP7A transporter activity [543]. Mtb, in turn, possesses specific machinery to counter Cu-mediated cellular damage [542]. Libardo et al. investigated the anti-mycobacterial properties of DAB-10 peptide, composed of an Amino Terminal Copper and Nickel (ATCUN) binding motif and a C-terminal domain from a cell-penetrating antimicrobial peptide [246], since Cu-ATCUN complex is known to act as a ROS generator [544]. The compound induced dose-dependent Mrx1-roGFP2 oxidation in Mtb and Msm, which was more pronounced in the latter case and correlated with its increased toxicity towards Msm [246]. Performance of DAB-10 required the presence of molecular oxygen, and elimination of catalase from the 7H9 medium significantly improved the bactericidal effects, supporting ROS involvement in the mode of action. Studies with the compound fused to a fluorophore revealed that its internalization into RAW264.7 cells occurs via clathrin-coated vesicles and its localization overlaps with the signal coming from a Cu-selective probe CS-1, which was further enhanced after lipopolysaccharides -induced activation [246]. During THP-1 infection, DAB-10 also promoted an *E_MSH_* shift toward more positive values. Finally, the toxic effect was significantly diminished when membrane-permeable Cu-chelators were added to the system, supporting the idea that the phagosomal metal pool is the source of Cu for ATCUN [246].

Above, we discussed that an acidic medium induces reductive stress in Mtb. In resting macrophages, Mtb affects phagosome maturation, including acidification; however, stimulation with interferon-γ allows this blockade to be overcome and pH values of 4.5–5.4 to be achieved [545]. AC2P36 was identified in high-throughput screenings for inhibitors of acidic pH-inducible signaling pathways [255]. In growth inhibition assays this drug demonstrated ~10-fold selectivity at pH 5.7 compared to 7.0 and its redox mode of action was confirmed by several observations [255]. First, it promoted reductive stress in the cytosol as revealed by roGFP2 imaging. Second, ROS levels, reported by the fluorescent dye CellROX green, increased under AC2P36 treatment and this effect was approximately 2-fold higher in acidic conditions. Finally, it demonstrated synergistic interactions with diamide and clofazimine, which was in line with AC2P36-mediated upregulation of thiol homeostasis and oxidative stress confronting genes in the transcriptome assays [255]. Since AC2P36 depleted the total intracellular thiol pool (more pronounced at pH 5.7) and was shown to covalently bind GSH in vitro, the authors hypothesized its mechanism of action. According to their hypothesis, external acidification promotes reductive stress which in turn increases the concentration of thiols that are capable of reacting with AC2P36. As a result, bacteria face dysregulation of redox homeostasis which in some way enhances oxidative stress [255]. 

**Figure 10:**
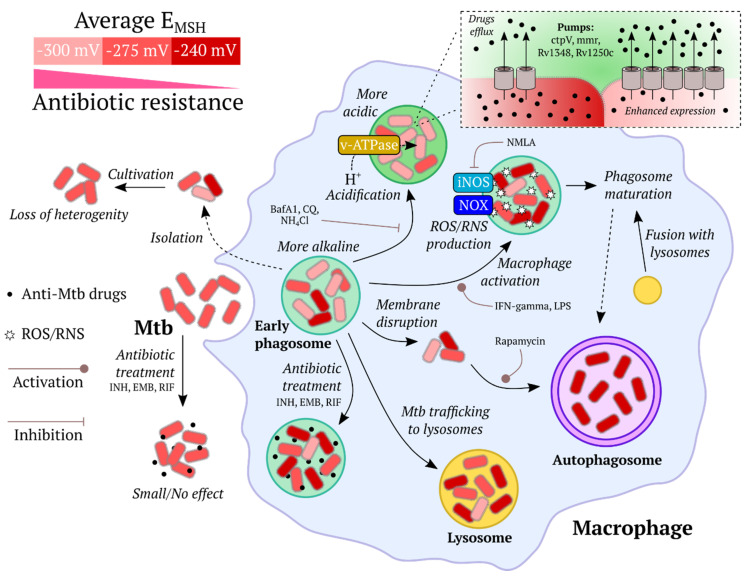
Internalization of *Mycobacterium tuberculosis* (Mtb) cells into macrophages leads to the emergence of subpopulations with different mycothiol redox potential (*E_MSH_*). Subsequent phagosome maturation is followed by an increase of the “oxidized” fraction size. Phagosomal acidification and antibiotic treatment also affect the average *E_MSH_* value. It is important to note, that the latter correlates with antibiotic resistance which is partially attributed to the differences in drug-efflux pumps expression [49,52]. Abbreviations: CQ—chloroquine; EMB—ethambutol; IFN—interferon; INH—isoniazid; iNOS—inducible NO synthase; LPS—lipopolysaccharides; NOX—NADPH oxidase; RIF—rifampicin; RNS—reactive nitrogen species; ROS—reactive oxygen species.

The lengthy treatment regimens required for the achievement of stable therapeutic effects in some bacterial diseases, including tuberculosis, are at least partially explained by the emergence of persister cells, a subpopulation of bacteria in which antibiotic tolerance results from metabolic reprogramming [546]. However, when the active compound is eliminated, these cells give rise to an antibiotic-sensitive population. Mtb persisters were detected in various animal and in vitro models as well as in tuberculosis patients [547,548]. Experimental evidence exists showing that the pathogen–host interaction includes metabolic cues that trigger bacteria to enter the persistence state. Isoniazid, ethambutol, rifampicin, and clofazimine shifted *E_MSH_* of sensitive Mtb cells residing in THP-1 cells to higher values in a time-dependent manner while only the last one reproduced that effect in 7H9 medium, suggesting that the remaining compounds did not induce oxidation *per se* but modulated the host responses (Figure 10) [52]. Interestingly, when Mtb strains resistant to isoniazid were tested, the signal of the sensor was not altered. Implementation of the cell viability marker propidium iodide revealed that the *E_MSH_*-reduced subpopulation was almost completely unaffected by antibiotics. Therefore, bacteria with a more reducing MSH pool seem to be capable of maintaining membrane integrity in the tested conditions (Figure 10). Given that Mtb *E_MSH_* was shown to be highest in phagosomes, the combinations of isoniazid or clofazimine with the autophagy inducer rapamycin significantly potentiated both redox shifts and bactericide power [52]. The described phenomenon was recently connected with the acid-base equilibrium in the phagosomal compartment (Figure 10) [49]. Thus, macrophages enriched in *E_MSH_*-reduced bacteria are more acidic than those enriched in the *E_MSH_*-oxidized subpopulation (the corresponding pH values are approximately 5.8 and 6.7). In line with that observation, implementation of phagosomal acidification inhibitors (bafilomycin, ammonium chloride, and chloroquine) was shown to lower the “reduced” fraction occupancy. Moreover, the toxic effect of isoniazid was enhanced up to 5-fold in the presence of bafilomycin and chloroquine [49]. Notably, presorted bacteria from different redox populations, which were grown in 7H9 medium, lost *E_MSH_* heterogeneity within 2 h, supporting the concept of phagosomal pH being a potent modulator of drug-resistance in Mtb [49]. Additional experiments with the U1 monocytic cell line model of HIV-Mtb coinfection confirmed the importance of pH-dependent redox homeostasis readjustment in this case. In particular, the “reduced” bacterial fraction was more widely represented in this system [49]. In contrast to a common point of view that persisters constitute a metabolically inactive fraction, this study registered a higher rate of replication clock plasmid (pBP10) loss in the subpopulation with lower *E_MSH_* compared to other groups. Finally, the transcripts of the drug-efflux pumps *ctpV*, *mmr*, *Rv1348*, and *Rv1250c* were elevated in the “reduced” fraction as along with their enzymatic activity that was indirectly measured using radiolabeled isoniazid (Figure 10) [49]. 

**Figure 11:**
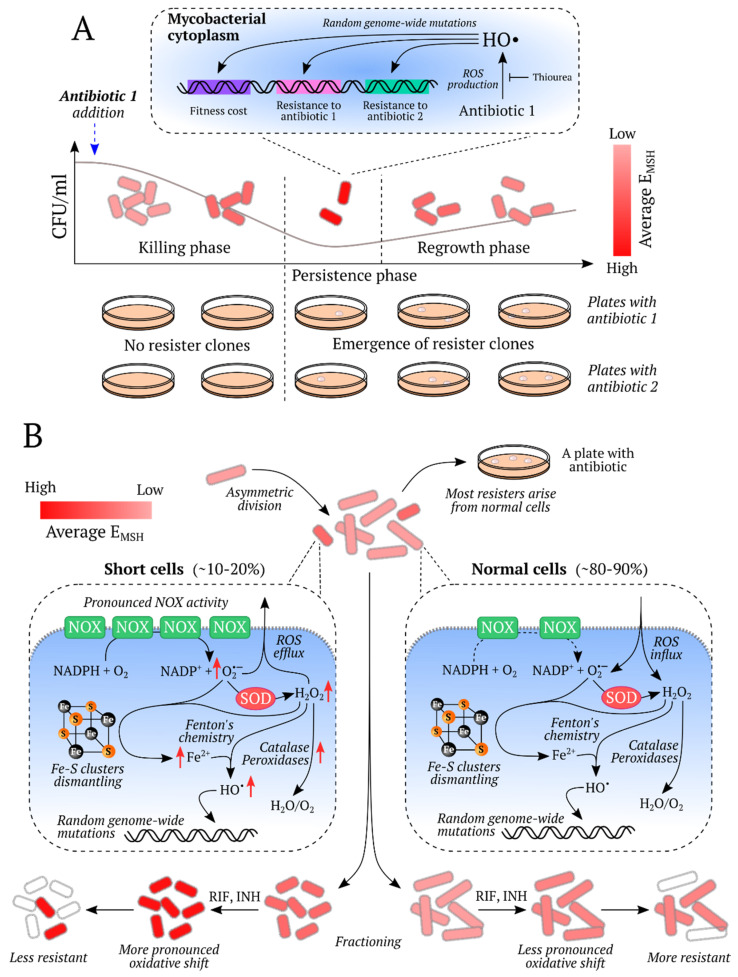
Redox processes that lead to the emergence of antibiotic resisters in mycobacteria. (**A**) Prolonged exposure of mycobacterial cells to antibiotic is characterized by three phases (“killing”, “persistence” and “regrowth”). The second phase is accompanied by elevated reactive oxygen species (ROS) production which stimulates genome-wide mutagenesis and consequent emergence of resisters. However, it should be mentioned that since DNA modification by ROS is random, the surviving cells experience a fitness cost due to undesired mutations [249,253]. (**B**) In mycobacterial populations, two fractions of cells can be found in regard to the cellular length, which result from asymmetric divisions. Short cells are characterized by elevated ROS production, which on the one hand lowers their resistance to environmental stresses, but on the other allows them to act as ROS-producing “factories” that stimulate the emergence of antibiotic resisters among normal cells [247,248]. Abbreviations: CFU—colony forming unit; *E_MSH_*—mycothiol redox potential; INH—isoniazid; NOX— NADPH oxidase; RIF—rifampicin; SOD—superoxide dismutase.

The development of antibiotic persistence by bacteria not only leads to the need for long-term treatment, which is an economical and a psychological burden for patients, but represents a medico-ecological threat, because this metabolically drug-tolerant fraction of pathogens might serve as a reservoir for the emergence of cells with genetic resistance. Sebastian et al. subjected Mtb cells to extended exposure to lethal doses of rifampicin or moxifloxacin for approximately three weeks, wherein samples were taken from the cultures daily and plated on antibiotic-free or –containing medium for colony-forming unit (CFU) counting [253]. The registered temporal dynamics of the CFU value on antibiotic-free plates was characterized by three phases: a killing phase with pronounced gradual decrease in the number of clones registered (~11 days), a persistence phase during which Mtb survival was not affected (~4 days) and a regrowth phase, where the CFU value began to recover (Figure 11A). During the second phase, a significant increase in the number of colonies on the antibiotic-containing medium was detected [253]. As confirmed by the modified Luria-Delbruck fluctuation test, the resisters resulted from *de novo* mutations rather than existed in the system from the beginning. Moreover, addition of thiourea to the experimental samples markedly reduced the mutation frequency suggesting the involvement of hydroxyl radicals in the generation of antibiotic resisters [253]. Indeed, electron paramagnetic resonance and imaging with hydroxyl radical-specific dye 3′-(phydroxyphenyl) fluorescein detected elevated production of ROS in Mtb during the persistence phase, while Mrx1-roGFP2 reported an oxidative shift in *E_MSH_*. Since hydroxyl radicals damage DNA in a sequence-independent manner, it was not a surprise that whole-genome sequencing of rifampicin resisters identified extensive mutations throughout the genome. In line with that, the authors demonstrated that moxifloxacin resisters could be generated from persister cells during rifampicin exposure [253]. Recently, similar observations were reproduced for Msm cells under moxifloxacin treatment, which indicates that the described three-phase dynamics with elevated oxidative stress in the persister cells is not unique for Mtb and might constitute a common trend not only for mycobacterial species, but for even wider groups of prokaryotes [249]. Interestingly, Mrx1-roGFP2 revealed that even in the lack of antibiotic, culture senescence led to higher *E_MSH_* values; however, these shifts were too modest to stimulate effective resisters emergence [249]. In general, both studies highlight that sub-optimal therapeutic strategies aimed to stimulate oxidative stress in bacteria might contribute to the development of antibiotic-resistant strains as well as lengthy antibiotic courses since they are not capable of eliminating the persisters, and also create evolutionary pressure for the selection of drug-resistant clones (Figure 11A). 

Besides factors introduced by interaction with the host, internal cues stimulate mycobacteria to divide intro various phenotypically and metabolically distinct subpopulations. Vijay et al. found that the actively growing mid-log phase mycobacteria were represented by two classes with different morphology: a minor subpopulation of low buoyant density short-sized cells (SCs), and a major subpopulation of high buoyant density normal/longsized cells (NCs) with a corresponding abundance ratio of ~1:9 [549,550]. Similar phenotypical groups could be distinguished among bacteria that were isolated from the sputum of pulmonary tuberculosis patients [550]. The observation that SCs are more sensitive to anti-tuberculosis compounds and H_2_O_2_ [551] suggested the possibility that these subpopulations differ by adjustment of their redox homeostasis. In contrast to NCs, both rifampicin and isoniazid triggered pronounced Mrx1-roGFP2 oxidation in SCs that could be diminished by thiourea administration [247]. The 3′-(phydroxyphenyl) fluorescein, Amplex Red, and dihydroethidium probes clearly showed that SCs respond to antibiotics by enhanced production of hydroxyl radicals, H_2_O_2_, and superoxide anions [247,248]. Interestingly, as described above, with the use of Mrx1-roGFP2, Bhaskar et al. did not observe shifts in mycobacterial *E_MSH_* under treatment with the same compounds [52]. It seems, that the natural fraction of SCs is too small to significantly influence the ratio of the sensor when the whole population is imaged. The specific NADH:flavin oxidoreductase/NADH oxidase, MSMEG_6603, was overexpressed in Msm SCs which correlated with increased NADH oxidase activity in cell lysates as well as with a decline in ROS production when DPI was introduced to the system [248]. Labile Fe^2+^, an important participant of Fenton reactions, was also elevated in SCs, most likely due to the metal leaching from [4Fe-4S] clusters induced by H_2_O_2_ and superoxide exposure [248]. Finally, the authors established that in the presence of rifampicin or moxifloxacin the frequency of antibiotic resisters emergence was ~2–5-fold higher in SCs; however, when mycobacteria were mixed in a natural-like proportion, most resisters originated from NCs. Moreover, any deviation from the natural ratio led to a decrease in the resistance to rifampicin for the whole population [248]. Since asymmetric division is the source of SCs, it seems that their existence makes adaptationistic sense. Thus, SCs might act as “ROS-factories” that produce reasonable levels of mutagens during stress conditions to the detriment of their own resistance to redox alterations (Figure 11B). Such logic is further supported by the fact that, despite facing oxidative burden, SCs demonstrate only moderate induction of antioxidant systems [248]. 

One possible option to minimize the development of antibiotic-resistance in microorganisms is to implement active compounds that alter the physiology of the host, not the pathogen. It is known that mycobacteria utilize virulence factors that stimulate mitochondrial superoxide-mediated necroptosis and subsequent release of intracellular bacteria, promoting infection [552]. On the contrary, apoptosis is an important regulator of mycobacterial proliferation [553,554,555]. Given that, implementation of antioxidants that lower mitochondrial ROS production is considered a potential therapeutic approach towards tuberculosis. Black et al. investigated the effects of 4-Methoxy-2,2,6,6-tetramethylpiperidine 1-oxyl (MetT), a stable cyclic nitroxide derivative based on TEMPO, on the *D. rario-Mycobacterium marinum* infection model [244]. Staining with the Cellrox, Mitosox and flavin-rhodamine redox sensor 2 probes revealed that mycobacteria-containing granulomas exhibited elevated ROS production, which was mitigated by MetT administration. Moreover, MetT demonstrated therapeutic effects, namely drug-treated embryos had significantly less bacterial burden after 5 days post infection. Correspondingly, at the same time point the number of TUNEL stained host cells per granuloma was markedly reduced in the experimental group [244]. However, it should be noticed that with TUNEL staining it is not possible to distinguish apoptotic cells from necroptotic cells. Peredox-mCherry did not reveal any direct impact of MetT on isolated *M. marinum*, but it did show NADH/NAD^+^ ratio growth when the compound was given to infected fish. The authors, therefore, hypothesize that MetT is capable of modulating the antibacterial response of the host, in particular, promoting the disruption of the mycobacterial ETC [244]. 

Finally, in another work with Peredox-mCherry, the application of different antibiotics (bedaquiline, ofloxacin, rifampicin, and isoniazid) to Msm cells internalized into RAW264.7 cells was also shown to increase the NADH/NAD^+^ ratio, most likely due to their ability to inhibit bacterial metabolism. However, the effect was significant only in the case of the latter. Notably, clofazimine elicited the opposite response [250]. The authors conclude that the Peredox sensor might be utilized in the search for small-molecule compounds that decrease the NADH/NAD^+^ ratio and, therefore, might improve the performance of antitubercular agents such as isoniazid and ethionamide [250].

### 5.8. Biotechnology

Some GEFIs can be used in biotechnological research. For instance, the real-time monitoring of the intracellular NADH/NAD^+^ ratio during methane to methanol bioconversion by the methanotrophic bacteria *Methylococcus capsulatus* was carried out by Ishikawa et al. [243]. It was shown that Peredox fluorescence reliably represented redox changes when certain chemicals were added. Reliable redox monitoring using the biosensor may facilitate the bioconversion process. Tejwani et al. applied the same probe to assess the NADH/NAD^+^ ratio under different metabolic conditions in the hydrogen-oxidizing bacterium *Ralstonia eutropha* [108]. The use of fluorescence lifetime data was shown to be equally or even more sensitive than the standard ratiometric readout. However, the authors found that in aerobically grown bacteria cells Peredox operated close to its saturation level, and this fact might complicate probe usage in such conditions. The cytoplasmic NADH concentrations were also measured in *R. eutropha* with Frex under different gas supply conditions [257]. Compared to Peredox, Frex exhibits lower affinity which prevents its saturation under typical bacterial NADH levels. Frex has also been used to monitor L-lactic acid production by *Lactobacillus paracasei* [241]. Using Frex, Tian et al. found values of extracellular oxidative and reductive potential, under which L-lactic acid production during the cell growth and stationary phases could increase significantly. RoGFP1-R12 has been used in another work by Liu et al., in which the modulation of respiration was suggested for maintenance of redox balance in *L. lactis* used for the production of certain chemicals [556].

## 6. Conclusions

Studying the processes based on the redox transformation of molecules is one of the most difficult tasks in the field of biology and medicine. As a rule, redox processes are characterized by high reaction rates. Development of the approaches for the registration of short-living compounds with high reactivity in the context of physiological processes is an important challenge. GEFIs have enhanced the methods of redox biology and brought biomedical research to a new level.

Initially, biosensors found wide application in cell models allowing detection of redox events in real time in the compartments of various cell types. However, the research of the mechanisms of complex biological processes, such as embryogenesis and aging, inflammation and regeneration, the functioning of tissues and organs in normal conditions and during the development of pathology, symbiotic interactions of organisms, the interplay between the host and the pathogen, and many others, requires in vivo models. Redox GEFIs are a powerful tool for in vivo studies in organisms of various levels of complexity: from prokaryotes to unicellular and multicellular eukaryotes. The in vivo approach allows valuable information about complex biological processes to be obtained since measurements can be taken non-invasively, preserving the integrity of cells and tissues, as well as the interactions between different organ systems. In this work, we reviewed and tried to systematize the latest advances in in vivo research using redox GEFIs.

However, in vivo studies make more and more demands on the properties of both existing biosensors and those under development. Testing the specificity of a biosensor is the most important task at the stage of its engineering. For example, sensors for *E_GSH_*, Grx1-roCherry and roGFP2-based probes, turned out to be oxidized by hypohalous acids and their derivatives [94,97,239]. It is, therefore, important to consider the effects of possible artifacts when interpreting the results. In particular, sensitivity to changes in pH is a pervasive problem for most cpFP-based biosensors. For example, it is necessary to monitor the pH dynamics when working with biosensors, such as HyPer-1,2,3 [66,100,101] and HyPerRed [103], for registration of H_2_O_2_; Frex probes [109], RexYFP [110], and SoNar [111] for registration of the NADH/NAD^+^ ratio. Not so long ago, the use of cpYFP as a biosensor for O_2_^•−^ became the topic of a heated discussion. Some researchers interpreted the change in the fluorescent signal of cpYFP as a consequence of O_2_^•−^ generation (“superoxide flashes”) [557,558], while others explained these changes by pH fluctuations [559]. However, later it was shown that cpYFP is unresponsive to O_2_^•−^ and demonstrates signal changes due to pH fluctuations in vitro [560]. It should be noted that some biosensors do not respond to physiological changes in pH. This refers to some ratiometric GEFIs, such as HyPer7 [102] and roGFP2-based probes [88], for which the pH-dependent changes of a signal are proportional for both excitation wavelengths and can therefore be normalized. However, there may be other reasons for the artifacts, and it is necessary to select the controls very carefully when using biosensors in vivo.

Low fluorescence intensity or small response amplitudes of biosensors can be another practical limitation for in vivo use. Registration of subtle physiological changes in tissues with such instruments can become difficult. This is especially true for in vivo studies on mammalian models. As a rule, in these systems the signal must be recorded in deep tissue structures, which requires special optical equipment. This explains the relatively small amount of work devoted to in vivo imaging of redox processes in mammals. The most convenient vertebral object for microscopy is *D. rerio*. The main advantage of this organism is its optical transparency at the embryonic and early juvenile stages. In addition, some mutant strains provide transparent adult fish [561]. 

Regardless of the biosensor used, it is important to remember that the introduction of a foreign protein into the organism can lead to unexpected effects that have an impact on physiological processes. Although it is generally accepted that fluorescent proteins are inert reporters, there are examples of their negative influence on intracellular processes in the literature [562,563,564]. Therefore, the effects of biosensors on the model organisms should be studied carefully.

Despite the above-mentioned possible limitations, biosensors have indisputable advantages over many other methods; they are gaining widespread popularity and are actively used for solving various questions. Development of biosensors for detection of new compounds is an important line of the future research that will open new perspectives for GEFIs application in experimental models. To date there are no sensors for (pseudo)hypohalous acids, singlet oxygen, HO^•^, O_2_^•−^. For measuring some compounds, namely ONOO^−^ [565,566] and H_2_S [567,568], there have been GEFIs published. However, their implementation is followed by pronounced drawbacks since their functioning is dependent on the interaction between the analyte and unnatural amino acid introduced into the structure of the protein. Improved understanding of the biosensors structure will allow obtaining probes with desired characteristics: bright, specific, with high response amplitude and fast kinetics. Obtaining the crystal structures of several Ca^2+^ probes allowed to identify key amino acid residues responsible for various properties of biosensors [569] and to perform rational design of new GEFIs with improved characteristics. For example, jRCaMP1a, b and jRGECO1a [570], GCaMP3 [341], GCaMP5 family sensors [571], GCaMP6 [339] and jGCaMP7 [572] were designed on the basis of the crystal structure of the previous versions of the Ca^2+^ indicators. It is important to note, that currently most of the probes are based on green or yellow FPs which limits the use of two instruments inside the organism simultaneously. Development of spectrally different biosensors will allow multiparameter imaging when several parameters are monitored at the same time [77]. 

A separate issue in the context of imaging with the use of GEFIs arises from the fact that shifts in the protein concentration or the thickness of biological sample can result in signal alterations that might be taken for changes of the specific parameters. The described problem is especially relevant when imaging with single FP-based sensors; however, FRET indicators are also prone to a number of artifacts. Common fluorescent proteins demonstrate relatively broad spectra which results in tangible bleedthrough making interpretation of ratiometric signal difficult. Moreover, ratiometric readout faces challenges in case of confocal microscopy. Depending on the depth of sample, patterns of light scattering for emission channels can differ notably leading to measurement artifacts, especially in case of in vivo imaging. A solution might be found in implementation of fluorescence lifetime imaging (FLIM) readout [573]. The main advantage of this approach is that fluorescence lifetime is a pure physical parameter independent of chromophore concentration, photobleaching and the settings of equipment (intensity of excitation light and optical path). Some papers are published that describe sensors that were specifically developed for FLIM measurements [574,575]. This readout was also implemented for imaging of cellular pH [576,577] and Ca^2+^ [578] fluctuations. HyPer family probes were also tested for their compatibility with this technology [66,579]. Generally, little is known whether single FP-based indicators might be used with FLIM. A recent review discusses this issue in regard to sensors based on G protein-coupled receptors [580]. 

Imaging of multicellular organisms is hampered by insufficient transparency of their tissues for visible light due to various factors including melanin and hemoglobin absorbance as well as relatively pronounced scattering. Moreover, long microscopic series lead to decrease in GEFIs brightness by the reason of photobleaching; this effect is more enhanced during widefield microscopy. These problems can be partially overcome by multiphoton microscopy methods. This approach is based on simultaneous excitation of a chromophore by several photons with wavelengths that are longer than that for the emission maximum. Multiphoton microscopy allows to shift the source of excitation to the infrared region which facilitates imaging of deep tissue regions. Since multiphoton absorption is characterized by low efficiency, this approach requires focusing the laser at a small sample volume which reduces photobleaching and improves signal-to-noise ratio. In recent years, this technique has been gaining popularity in the field of redox biology [62,321,579]. We are sure that improvement of biosensors, as well as the approaches for their visualization inside living organisms, will provide further progress for in vivo biomedical studies.

## Figures and Tables

**Table 1:** Some important characteristics of redox-sensitive GEFIs applied in vivo.

Analyte	Name	λex	λem	Response Amplitude	Midpoint Potential/EC_50_/Kd/Ks	Reference
general redox state	Oba-Qc	430	480	~2	MP = −249 mV	[95]
NADH/NAD^+^	Peredox	400/587	510/610	2.5	Kd to NADH <5 nM for initial P0 construct	[107]
H_2_O_2_	NeonOxIrr	508	520	2.8 (in vitro)	NM	[105]
NADH	Frex	420/500	518	9 (fl. increase)	Kd~3.7 μM at pH 7.4	[109]
NADH	FrexH	420/500	518	3 (fl. decrease)	Kd~40 nM	[109]
NADH/NAD^+^	RexYFP	490	516	~2 (in vitro)	K’(NADH) = 180 nM, K’(NADPH) = 6.2 μM	[110]
NADH/NAD^+^	SoNar	420/485	528	15	Kd(NADH) ~0.2 μM, Kd(NAD^+^) ~5.0 μM at pH 7.4	[111]
NAD^+^/AXP (AXP = ATP + ADP)	FiNad	485	~520	~7	Kd(NAD^+^) shifts from ~14 μM to ~1.3 mM in the presence of ATP or ADP	[112]
NADPH	iNaps	420/500	515	10	Kd = 2.0 ÷ 120 μM	[75]
*E_GSH_*	rxYFP	512	523	2.2 (in vitro)	MP = −261 mV	[93]
*E_GSH_*	roGFP1	400/475	508	~6 (in vitro)	MP = −294 mV	[85,117]
*E_GSH_*	roGFP1-Rx family	395/475	NM	5.4–7.5	MP = −263 mV to −284 mV	[86]
*E_GSH_*	roGFP1-iX	395/465	505	2.4–7.2	MP = −229 mV to −246 mV	[87]
*E_GSH_*	roGFP2	400/490	511	~6 (in vitro)	MP = −287 mV	[85,117]
general redox state	roUnaG	498	527	~9 (in vitro)	MP = −275 mV	[99]
*E_GSH_*	Grx1-roGFP2	400/490 *	511	~4.4 (in living cells)	MP = −280 mV	[88]
*E_MSH_*	Mrx1-roGFP2	390/490	510	~8 (in vitro)	MP = −280 mV	[52]
*E_BSH_*	Brx-roGFP2	405/488	NM	~4 (in vitro)	NM	[89]
*E_T(SH)2_*	Tpx-roGFP2	400/490 *	511	NM	NM	[90]
H_2_O_2_	TriPer	405/488	NM	NM	NM	[104]
H_2_O_2_	HyPer	420/500	516	3.3 (in vitro)	Ks = 5 × 10^5^ M^−1^ s^−1^	[66,100]
H_2_O_2_	HyPer-2	420/500	516	~6 (HeLa cells)	Ks = 1.2 × 10^5^ M^−1^ s^−1^	[66,101]
H_2_O_2_	HyPer-3	420/500	516	~6 (HeLa cells)	Ks = 2.5 × 10^5^ M^−1^ s^−1^	[66]
H_2_O_2_	HyPer7	400/499	516	~10 (in vitro)	i.v. = 26.9 ± 0.28 a.u./s (for HyPer3 it is 0.315 ± 0.007 a.u./s)	[102]
H_2_O_2_	roGFP2-Orp1	400/490 *	511	4.8 (HeLa cells)	responds to low micromolar concentrations of exogenously applied H_2_O_2_ (HeLa cells)	[91]
H_2_O_2_	roGFP2-Tsa2ΔC_R_	400/490 *	511	~6 (in vitro)	low-nanomolar or high-picomolar endogenous H_2_O_2_ concentrations	[92]
*S*-MetO	MetSOx	425/505	510–516	~6	K_0.5_ = 0.5 μM (in vitro)	[113]
*R*-MetO	MetROx	410/500	510–516	~6	K_0.5_ = 177 μM (in vitro)	[113]
ROOH	OHSer	519	526	2	NM	[114]
general redox state	rxRFP	576	600	4	MP = −290 mV	[96]
*E_GSH_*	Grx1-roCherry	589	610	1.5	MP = −311 mV (pH = 7.0)	[97]
H_2_O_2_	HyPerRed	575	605	~2	Ks = 3 × 10^5^ M^−1^ s^−1^	[103]
general redox state	Redoxfluor	NM	476/527	~1.5 (H_2_O_2_ in vitro)	MP = −213 mV (pH = 7.0)	[44]
Trx redox state	CROST	NM	480/530	~3	MP (CROST1) = −266 mV (pH = 7.5) MP(CROST2) = −296 mV (pH = 7.5)	[116]
NADP^+^	NADPsor	NM	478/526	~1.4	Kd = 2 mM, detection limit = 1 μM (for NADPsor-K1-K72W version)	[115]

* The spectral properties of biosensors derived from roGFP2 are very similar to those of roGFP2 itself, therefore, for these samples, the value determined for roGFP2 is given. Background colors correspond to the colors of fluorescent proteins (FPs) used as reporter domains. Abbreviations: *E_BSH_*—bacillithiol redox potential; *E_GSH_*—glutathione redox potential; *E_MSH_*—mycothiol redox potential; *E_T(SH)2_*—trypanothione redox potential; i.v.—initial velocity (see [102]); K_0.5_—the half-maximal concentration constant; K’—affinity constant; Kd—dissociation constant; Ks—the pseudo-first order reaction rate constant; MetO—methionine sulfoxide; MP—midpoint potential; NM—not measured. Background colors in this table correspond to the colors of fluorescent proteins (FPs) used as reporter domains.

**Table 2:** Model organisms in which redox GEFIs were applied.

*Model organism*	*Parameter*	*GEFI*	*References*
**Animals**			
*Caenorhabditis elegans*	*E_GSH_*	roGFP1	[22,23,34]
		roGFP2	[118]
		roGFP	[119]
		roGFP1-R12	[50]
		Grx1-roGFP2	[120,121,122]
	H_2_O_2_	HyPer	[34,70,120,123,124,125,126]
		HyPer-2	[127]
		roGFP2-Orp1	[128,129,130]
		roGFP2-Tsa2ΔC_R_	[129]
	NADH/NAD^+^	Peredox	[56,131]
*Drosophila melanogaster*	*E_GSH_*	roGFP2	[61]
		Grx1-roGFP2	[18]
	H_2_O_2_	roGFP2-Orp1	[18,132]
*Danio rerio*	*E_GSH_*	roGFP2	[133]
		Grx1-roGFP2	[134,135,136,137,138]
		Grx1-roCherry	[97]
	NADH/NAD^+^	SoNar	[139]
		RexYFP	[64]
	NAD^+^/AXP, AXP = ATP + ADP	FiNad	[112]
	NADPH	iNap	[75,139]
	H_2_O_2_	HyPer	[57,58,63,65,67,68,69,140,141,142]
		HyPer-3	[66]
		HyPerRed	[75,139]
		HyPer7	[102]
		roGFP2-Orp1	[137,138]
*Mus musculus*	*E_GSH_*	roGFP1	[53,54,143,144]
		roGFP2	[60]
		roGFP	[21,145,146,147]
		Grx1-roGFP2	[55,62]
	NADH/NAD^+^	Peredox	[76]
		SoNar	[111,148,149]
	NAD^+^/AXP	FiNad	[112]
	H_2_O_2_	roGFP2-Orp1	[150]
		NeonOxIrr	[105]
*Xenopus laevis*	H_2_O_2_	HyPer	[71,72,151]
**Plants**			
*Arabidopsis thaliana*	*E_GSH_*	roGFP	[152]
		roGFP1	[153,154,155,156,157,158]
		roGFP2	[153,159,160,161,162,163,164,165,166,167]
		Grx1-roGFP2	[168,169,170,171,172,173,174,175,176,177]
		roGFP2-iL	[178]
		GRX1-roGFP2-iL	[178]
	Trx redox state	CROST	[116]
	H_2_O_2_	roGFP2-Orp1	[174,179]
		HyPer	[180,181,182,183,184,185]
*Medicago truncatula*	H_2_O_2_	HyPer	[186]
*Nicotiana benthamiana*	H_2_O_2_	HyPer	[187]
		HyPer-2	[188]
*Nicotiana tabacum*	*E_GSH_*	roGFP1	[153]
		roGFP2	[153,189]
*Solanum lycopersicum*	*E_GSH_*	roGFP1	[190]
**Fungi**			
*Botrytis cinerea*	*E_GSH_*	roGFP2	[191,192]
		Grx1-roGFP2	[191]
*Cochliobolus heterostrophus*	H_2_O_2_	HyPer	[193]
*Fusarium graminearum*	H_2_O_2_	HyPer-2	[194]
*Magnaporthe oryzae*	*E_GSH_*	Grx1-roGFP2	[195]
	H_2_O_2_	MoHyPer	[196]
*Pichia pastoris*	*E_GSH_*	roGFP1	[37,197]
		roGFP1-iE	[37,197,198]
		roGFP1-iL	[198]
	general redox state	Redoxfluor	[44]
*Saccharomyces cerevisiae*	*E_GSH_*	rxYFP	[31,43,46,199,200,201]
		roGFP1	[25,26,27,28,29,202,203]
		roGFP2	[12,19,33,204,205,206]
		roGFP	[207]
		eroGFP	[35,36,38,39,40,41,42,208]
		Grx1-roGFP2	[15,24,32,47,48,209,210,211,212,213,214]
	general redox state	rxRFP	[30]
		Redoxfluor	[44,215]
	H_2_O_2_	HyPer	[92]
		HyPerRed	[216]
		roGFP2-Orp1	[13,214,217]
		roGFP2-Tsa2ΔC_R_	[14,15,92,206]
		roGFP2-Tsa2ΔC_R_ΔC_P_	[92]
*Schizosaccharomyces pombe*	H_2_O_2_	HyPer	[218]
		roGFP2-Tpx1.C169S *	[218]
*Ustilago maydis*	NADH/NAD^+^	Peredox	[219]
**Eukaryotic unicellular organisms**			
*Chlamydomonas reinhardtii*	*E_GSH_*	ObaQc	[220]
*Phaeodactylum tricornutum*	*E_GSH_*	roGFP	[221,222]
*Plasmosium falciparum*	*E_GSH_*	Grx1-roGFP2	[16,73,223,224,225]
		sfroGFP2	[73]
	H_2_O_2_	HyPer-3	[226]
		roGFP2-Orp1	[17,226]
*Toxoplasma gondii*	*E_GSH_*	roGFP1, roGFP-iL	[45]
*Trypanosoma brucei brucei*	*E_GSH_/E_T(SH)2_*	Grx1-roGFP2	[90,227,228,229,230,231]
		roGFP2	[90]
	*E_T(SH)2_*	Tpx-roGFP2	[90,232]
**Bacteria**			
*Caulobacter crescentus*	*E_GSH_*	roGFP2	[233]
*Chlamydia trachomatis*	*E_GSH_*	roGFP2	[234]
*Citrobacter rodentium*	*E_GSH_*	roGFP2	[235]
*Corynebacterium glutamicum*	*E_MSH_*	Mrx1-roGFP2	[74]
*Escherichia coli*	*E_GSH_*	rxYFP	[93]
		roGFP1	[236]
		roGFP2	[235,237,238,239]
		Grx1-roGFP2	[239,240]
	general redox state	roUnaG	[99]
	H_2_O_2_	roGFP2-Orp1	[239,240]
	*S*- and *R*-MetO	MetSOx, MetROx	[113]
*Lactobacillus paracasei*	NADH	Frex	[241]
*Lactococcus lactis*	*E_GSH_*	roGFP1-R12	[242]
*Methylococcus capsulatus*	NADH/NAD^+^	Peredox	[243]
*Mycobacterium marinum*	NADH/NAD^+^	Peredox	[244]
*Mycobacterium smegmatis*	*E_MSH_*	Mrx1-roGFP2	[52,245,246,247,248,249]
	NADH/NAD^+^	Peredox	[250]
*Mycobacterium tuberculosis*	*E_MSH_*	Mrx1-roGFP2	[49,52,245,246,251,252,253]
		roGFP1-R12	[254,255]
	NADH/NAD^+^	Peredox	[250]
*Pantoe eucalypti*	*E_GSH_*	roGFP2	[256]
*Ralstonia eutropha*	NADH/NAD^+^	Peredox	[108]
	NADH	Frex	[257]
*Salmonella* Typhi	*E_GSH_*	roGFP2	[235]
*Salmonella* Typhimurium	*E_GSH_*	roGFP2	[235,258,259]
*Staphylococcus aureus*	*E_BSH_*	Brx-roGFP2	[89,260,261,262]
	H_2_O_2_	Tpx-roGFP2 *	[260]
*Streptococcus oligofermentans*	H_2_O_2_	HyPer	[263]
*Synechococcus elongatus*	*E_GSH_*	roGFP1	[264]
*Yersinia pseudotuberculosis*	*E_GSH_*	roGFP2	[235]

* Tpx-roGFP2 means here the peroxiredoxin-based probe that is specific to H_2_O_2_. Abbreviations: AXP—pool of ATP and ADP; *E_BSH_*—bacillithiol redox potential; *E_GSH_*—glutathione redox potential; *E_MSH_*—mycothiol redox potential; *E_T(SH)2_*—trypanothione redox potential; MetO—methionine sulfoxide; Trx—thioredoxin.

**Table 3:** Transgenic organisms with redox sensors targeted to nervous cells.

Transgene	Species	Redox Sensor	Target	References
*Thy1-mito-Grx1-roGFP2*	*Mus musculus*	Grx1-roGFP2	mitochondria of neurons in the CNS and the PNS	[62]
*Thy1-roGFP1c*	*Mus musculus*	roGFP1	cytosol of neurons in the CNS and the PNS	[59]
*Thy1-roGFP1m*	*Mus musculus*	roGFP1	mitochondria of neurons in the CNS and the PNS	[59]
*TH-mito-roGFP2*	*Mus musculus*	roGFP2	mitochondria of dopaminergic neurons	[60]
*elav-Gal4; UAS-MTSroGFP2*	*Drosophila melanogaster*	roGFP2	mitochondria of neurons	[61]
*ubi-HyPer*	*Danio rerio*	HyPer	ubiquitous expression	[63]
*myo6b-REX-YFP*	*Danio rerio*	REX-YFP	hair cells of lateral-line system	[64]

Abbreviations: CNS—central nervous system, PNS—peripheral nervous system.

## Abbreviations

2CP	2-Cys peroxiredoxin
AA	arachidonic acid
ATCUN	Amino Terminal Copper and Nickel binding motif
AXP	total pool of ATP and ADP
benzylIMD	3-[substituted-benzyl]-menadiones
BSH	reduced bacillithiol
BSSB	oxidized bacillithiol
CFU	colony-forming unit
CNS	central nervous system
cp	circularly permuted
CQ	chloroquine
CuOOH	cumene hydroperoxide
DBMIB	2,5-dibromo-6-isopropyl-3-methyl-1,4-benzoquinone
DCMU	3-(3,4-dichlorophenyl)-1,1-dimethylurea
DPI	diphenyleneiodonium
dppf	1,1′-bis(diphenylphosphino) ferrocene
DPS	4,4′-dipyridyl disulfide
DR	diet-restricted
EB	elementary bodies
*E_BSH_*	bacillithiol redox potential
*E_GSH_*	glutathione redox potential
EMB	ethambutol
*E_MSH_*	mycothiol redox potential
*E_T(SH)__2_*	trypanothione redox potential
ER	endoplasmic reticulum
ETC	electron transport chain
FD	ferredoxin
FF	fully fed
FLIM	fluorescence lifetime imaging
FNR	ferredoxin-NADP^+^ reductase
FP	fluorescent protein
FRET	Förster resonance energy transfer
FTR	(Fd)-dependent Trx reductase
G6PD	glucose-6-phosphate dehydrogenase
GEFIs	genetically encoded fluorescent indicators
GFP	green fluorescent protein
GR	glutathione reductase
Grx1	glutaredoxin 1
GSH	reduced glutathione
GSSG	oxidized glutathione
hpi	hours post infection
IFN	interferon
IMS	intermembrane space of mitochondria
INH	isoniazid
iNOS	inducible NO synthase
L	lineage
LPS	lipopolysaccharides
MetT	4-Methoxy-2,2,6,6-tetramethylpiperidine 1-oxyl
*mlt*	*meltdown*
MPO	myeloperoxidase
MSH	reduced mycothiol
Msm	*Mycobacterium smegmatis*
MSSM	oxidized mycothiol
Mtb	*Mycobacterium tuberculosis*
MTH1	mutT homologue
Myo	myosin
NCs	normal/long-sized cells
NOX	NADPH-oxidase
NTRC	NADPH-dependent thioredoxin reductase
OxyR-RD	regulatory domain of OxyR transcription factor
PI3K	phosphoinositide 3-kinase
PKC	protein kinase C
PLB	plumbagin
PNS	peripheral nervous system
PMA	phorbol 12-myristate 13-acetate
RACF	retrograde actin cable flow
RB	reticulate bodies
RBCs	red blood cells
RIF	rifampicin
RNS	reactive nitrogen species
roGFP	redox-sensitive green fluorescent protein
ROS	reactive oxygen species
rxRFP	redox sensitive red fluorescent protein
rxYFP	redox-sensitive yellow fluorescent protein
SCs	short-sized cells
SCV	*Salmonella*-containing vacuole
SEM	standard error of the mean
SHAM	salicylhydroxamic acid
sigH	SigmaH factor
SPI2	*Salmonella* pathogenicity island
SOD	superoxide dismutase
T3SS	type 3 secretion system
T-REX	Rex protein from *Thermus aquaticus*
T(SH)_2_	reduced trypanothione
Tpx	tryparedoxin
Trx	thioredoxin
TS_2_	oxidized trypanothione
UPR	unfolded protein response
WT	wild type
YFP	yellow fluorescent protein
